# Spatial Orientation‐Dependent Double Hydrogen Bond/Photoredox Cooperative Catalysis in Metal–Organic Frameworks

**DOI:** 10.1002/anie.5181076

**Published:** 2026-06-07

**Authors:** Jing Ouyang, Sze Lam Lam, Chao Li, Peiqi Zhang, Le Yang, Zhiyi Yang, Teng‐Teng Chen, Xueliang Xiao, Yangjian Quan

**Affiliations:** ^1^ Department of Chemistry The Hong Kong University of Science and Technology (HKUST) Hong Kong Hong Kong SAR China; ^2^ Key Lab of Special Protective Textiles Ministry of Education Jiangnan University Wuxi China

**Keywords:** bifunctional materials, metal–organic framework, synergistic catalysis

## Abstract

Catalyst activity is typically the primary priority in designing a catalytic system. The higher catalyst activity is envisioned to confer more powerful and effective catalytic performance. Here, we report a heterogeneous double hydrogen bond/photoredox synergistic catalysis, wherein the flexibility and spatial orientation of catalytic centers, rather than catalyst activity, play a dominant role in catalytic performance. We synthesized six metal–organic frameworks (MOFs) with photocatalytic and double hydrogen bond donor (DHBD) centers. **MOF‐F‐TU** featuring the relatively flexible (F) thiourea (TU) center has proved most effective in promoting dehalogenation and reductive coupling reactions. In contrast, **MOF‐R‐SQA1** with a stronger but rigid (R) squaramide (SQA1) site exhibits no catalytic activity. Mechanistic studies rationalize this unique phenomenon by implying a cooperative activation of the substrate via its double hydrogen bonding with DHBD and π–π overlapping with photocatalytic center, which is closely associated with the spatial arrangement of synergistic catalytic centers. **MOF‐F‐TU** also outperforms homogeneous counterparts, displaying turnover numbers of up to 9500.

## Graphical abstract

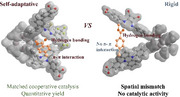

## 1 Introduction

The intrinsic activity of catalytic centers has long been regarded as the primary determinant of catalytic performance. However, this paradigm appears ambiguous in nature‐evolved catalysis, where relatively less active catalytic centers often facilitate transformations under mild reaction conditions [1, 2, 3, 4]. More interestingly, nature‐evolved catalysis has proven highly effective in activating inert molecules (e.g., N_2_). To pursue comparable catalytic performance and understand the function mechanisms behind, researchers have devoted substantial efforts to exploring bio‐inspired catalysis [5, 6, 7]. Albeit significant advancements, challenges persist in the precise construction of a self‐adaptive pocket involving multiple catalytic centers and in comprehending the catalytic behaviors.

Metal–organic frameworks (MOFs) represent a class of porous materials [8, 9, 10, 11, 12, 13, 14, 15, 16, 17, 18, 19, 20, 21, 22] with diverse applications in gas adsorption/separation [23, 24, 25, 26], drug delivery [27, 28, 29], anticancer therapies [30, 31], and catalysis [32, 33, 34, 35, 36, 37, 38, 39, 40, 41, 42, 43, 44, 45, 46, 47, 48, 49, 50, 51, 52, 53, 54, 55, 56, 57, 58]. Their porous structure and versatile capability to hold multiple catalytic centers in an organized and controllable manner significantly expand the platform for engineering bio‐inspired catalysis [59, 60, 61, 62, 63, 64, 65, 66, 67, 68, 69, 70, 71]. However, in the pursuit of synthesizing highly crystalline MOFs, research attention typically focuses on relatively rigid ligands, sacrificing the flexibility, and adaptivity of the catalytic system. These properties are indeed crucial in natural‐evolved catalysis [72, 73, 74, 75, 76, 77]. Although several seminal and pioneering examples have demonstrated the importance of flexibility in MOF catalysis [78, 79, 80, 81, 82, 83, 84], more contributions are desirable to correlate catalysts’ criteria (activity, proximity, orientation, and adaptivity) with catalytic performance, reflecting the catalytic mode biocatalysis adopts.

**FIGURE 1:**
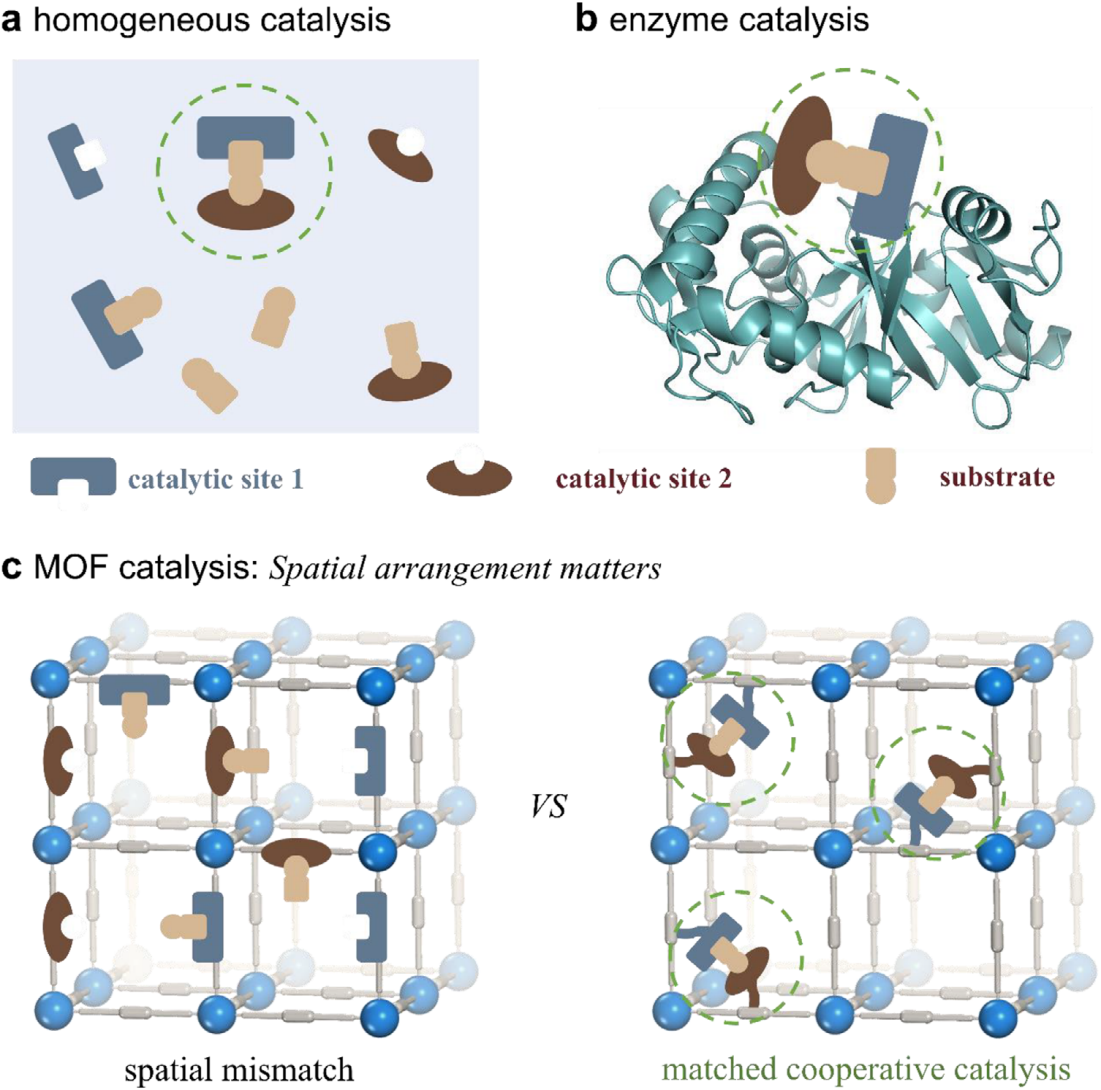
Cooperative catalysis. (a) Flexibility in homogeneous catalysis. (b) Adaptivity in enzyme catalysis. (c) This work: The role of spatial arrangement in MOF catalysis.

In homogeneous systems, catalysts engage with substrates in a relatively flexible manner, allowing for adjustable spatial orientation and distance between catalysts and substrates (Figure 1a). This situation changes in heterogeneous catalysis, where the position and conformation of catalytic centers are often relatively fixed (Figure 1c). For accessing superior catalytic performance, a self‐adaptive catalytic environment with proper spatial arrangement of active sites gains more weight, which however remains difficult to realize. Here, we report the design of **MOF‐DHBD** (DHBD = double hydrogen bond donor), possessing PZDB (PZDB = 4,4’‐(phenazine‐5,10‐diyl) dibenzoate) photosensitizer and DHBD centers, for double hydrogen bond/photoredox synergistic catalysis. These **MOF‐DHBDs** have rigid framework structures, but the flexibility of their DHBD centers is different [85, 86]. Catalytic performance evaluation highlights the importance of DHBD's flexibility over activity. The spatial mismatch between PZDB and a strong yet rigid DHBD site even led to no catalytic activity. Conversely, the optimized **MOF‐F‐TU** featuring a flexible thiourea center effectively promoted dehalogenation, dehalogenative arylation, remote C─H functionalization, and decarboxylative alkylation, achieving turnover numbers of up to 9500.

## 2 Results and Discussion

**FIGURE 2:**
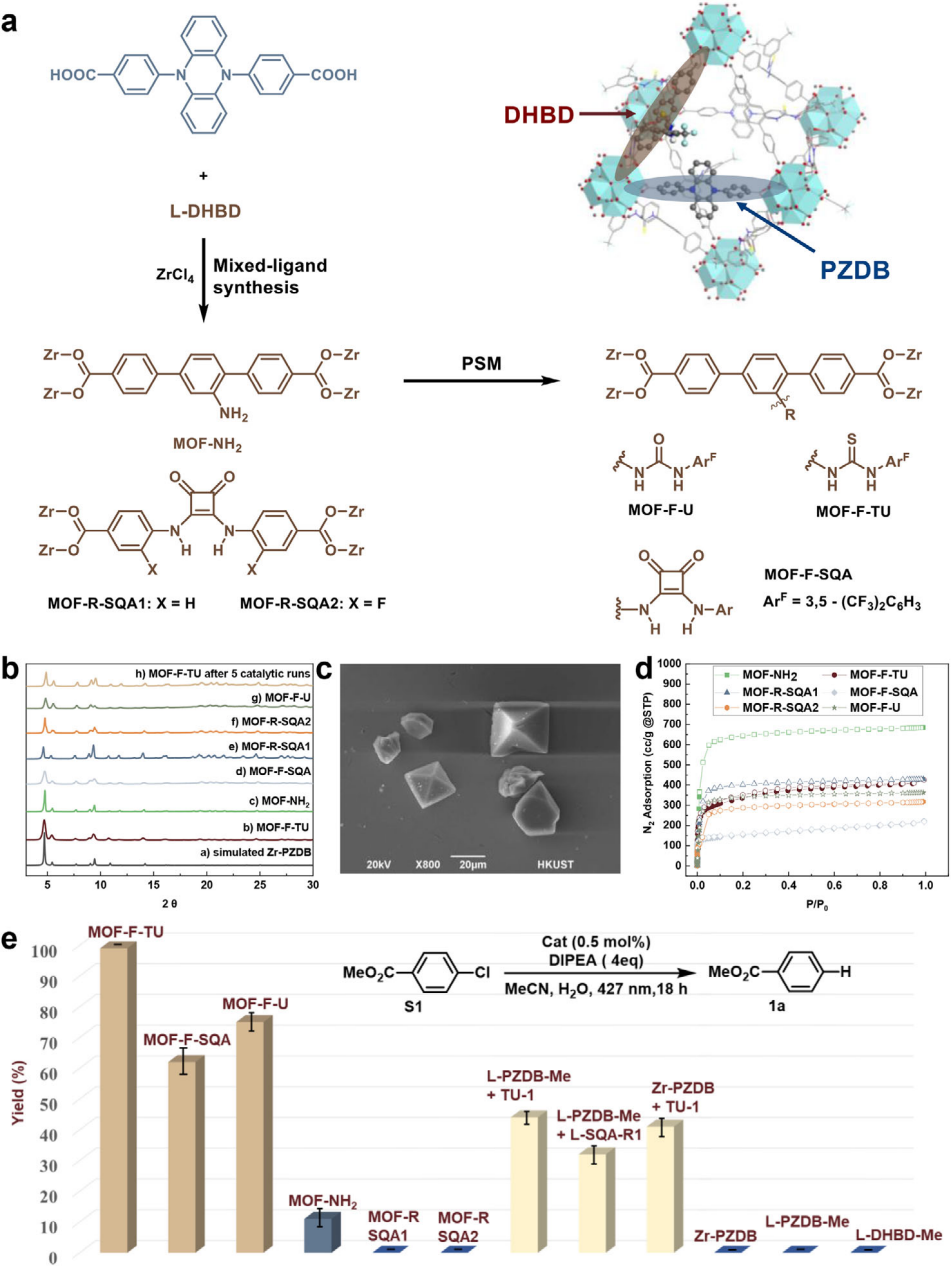
Synthesis, characterization, and catalytic performance of **MOF‐DHBDs**. (a) Synthetic procedure outline. (b) PXRD patterns of **MOF‐DHBDs**. (c) SEM of **MOF‐F‐TU**. (d) N_2_ (77 K) adsorption and desorption isotherms for the activated **MOF‐DHBDs**. (e) Yields (with error bar) of the model reaction catalyzed by **MOF‐DHBDs** and the homogeneous counterparts.

We synthesized six model **MOF‐DHBD** catalysts to systematically investigate their cooperative catalytic behaviors (see Figure 2 for MOFs preparation and characterization, *vide infra*). The DHBD centers integrated involve decorated urea (U) [87], thiourea (TU) [88, 89, 90], squaramides (SQA) [91, 92, 93], and aniline (NH_2_) units [94]. The mixed‐ligand synthetic approach was successfully applied to yield **MOF‐NH_2_**, **MOF‐R‐SQA1**, and **MOF‐R‐SQA2** as orange octahedral‐shaped crystals. Post‐synthetic modifications (PSM) of **MOF‐NH_2_** sequentially produced **MOF‐F‐U**, **MOF‐F‐TU**, and **MOF‐F‐SQA** (Figure 2a) [95, 96, 97]. The high crystalline purities of the prepared MOFs were evidenced by their powder XRD patterns (Figure 2b), matching the simulated patterns derived from the single crystal structure of **Zr‐PZDB** [98]. The porosities of **MOF‐DHBDs** were assessed through nitrogen adsorption–desorption analyses at 77 K (Figures 2d and S29–S34). Type I isotherms were observed, indicating Brunauer–Emmett–Teller (BET) surface areas of 2241, 1275, 1070, 1473, 965, 587 m^2^/g for **MOF‐NH_2_**, **MOF‐F‐TU**, **MOF‐F‐U**, **MOF‐R‐SQA1**, **MOF‐R‐SQA2**, and **MOF‐F‐SQA**, respectively. Pore‐size distribution was also studied based on QSDFT analysis. **MOF‐NH_2_**, **MOF‐F‐TU**, **MOF‐F‐U**, **MOF‐R‐SQA1**, and **MOF‐R‐SQA2** exhibited a similar major pore size of 12–14 Å, while **MOF‐F‐SQA** showed a markedly reduced pore size of 6 Å probably due to its structure damage during PSM. Scanning electron microscopy (SEM) imaging revealed the octahedral morphology of **MOF‐DHBDs** with an average size of approximately 20 µm (Figures 2c and S35). The UV‐Vis spectra of **MOF‐DHBDs** exhibited similar characteristic peaks to those of PZDB and DHBD ligands (Figures S36–S38). Solid‐state UV–vis diffuse reflectance spectra of **MOF‐DHBDs** were also collected, with calculated band gaps of 2.05–2.36 eV from Tauc plots (Figure S39). All MOFs showed broad IR peaks assigned to active protons of N─H and O─H units, meanwhile the signals of ν(COO–Zr) were also assignable (Figure S40). The IR signal assignable to the C═O bond in squaramide (1795 cm^−1^) was observed for **MOF‐F‐SQA**, **MOF‐R‐SQA1**, and **MOF‐R‐SQA2**. However, the characteristic signals of urea and thiourea were not clearly distinguishable, due to their overlap with other signals. ^1^H NMR analyses of digested MOFs were used to identify the ratios between PZDB and DHBD centers in **MOF‐DHBDs** (Figures S16–S22). The resulting chemical formulas of MOFs were further verified by the TGA results (Figures S23–S28).

Dechlorination was initially selected as a model reaction to evaluate the catalytic capability of **MOF‐DHBDs**. Among MOFs we synthesized, relatively flexible **MOF‐F‐TU**, **MOF‐F‐SQA**, and **MOF‐F‐U** provided good yields (62% ‒ quant., Figure 2e) in the photoreduction of 4‐carbomethoxyphenyl chloride (**S1**, *E* ^red^ ≈ −2.0 eV), while **MOF‐R‐SQA1** and **MOF‐R‐SQA2** featuring an even stronger DHBD catalytic center were proved ineffective [99]. To gain insight into this unique catalytic phenomenon, homogeneous controls were sequentially performed. A combination of L‐PZDB‐Me and L‐TU‐Me in CH_3_CN only gave a yield of 8%, due to the low solubility of L‐TU‐Me. Therefore, TU‐1 (1,3‐bis[3,5‐bis(trifluoromethyl)phenyl]thiourea) with a higher solubility was synthesized as an analogue to L‐TU‐Me. Its combination with L‐PZDB‐Me resulted in a yield of 44%. Meanwhile, the homogeneous mixture of L‐PZDB‐Me and L‐SQA‐R1 was catalytically active to deliver methyl benzoate in 32% yield. These results indicate that (1) both TU and SQA centers are catalytically effective in a homogeneous system because of their adjustable engagement with substrates; and (2) the dramatic difference (quant. vs. none) in catalytic performance between **MOF‐F‐TU** and **MOF‐R‐SQA1** is probably due to their distinct spatial arrangement of catalytic centers, that leads to disparate interaction modes with substrates.

Individual **Zr‐PZDB**, L‐PZDB‐Me, or TU‐1 did not catalyze the model reaction, suggesting the synergistic catalysis nature (Table S2). The PZDB/TU ratio of the optimized **MOF‐F‐TU** was measured as ∼ 1:2 (Figure S17). Increasing the proportion of PZDB photosensitizer resulted in reduced catalytic activity, further supporting the nature of cooperative catalysis. **MOF‐F‐U** with a similar structure to **MOF‐F‐TU** gave a yield of 72%, owing to its relatively weak hydrogen bond donor ability. **MOF‐F‐SQA** exhibited a PZDB/SQA ratio of 2.4/1 due to the incomplete conversion of the ─NH_2_ moiety to SQA, which might account for its relatively low catalytic efficiency (62%) compared with **MOF‐F‐TU** (quant.). As predicted, **MOF‐NH_2_** with a weak hydrogen bond donor only gave a yield of 11% (Figure 2e).

**FIGURE 3:**
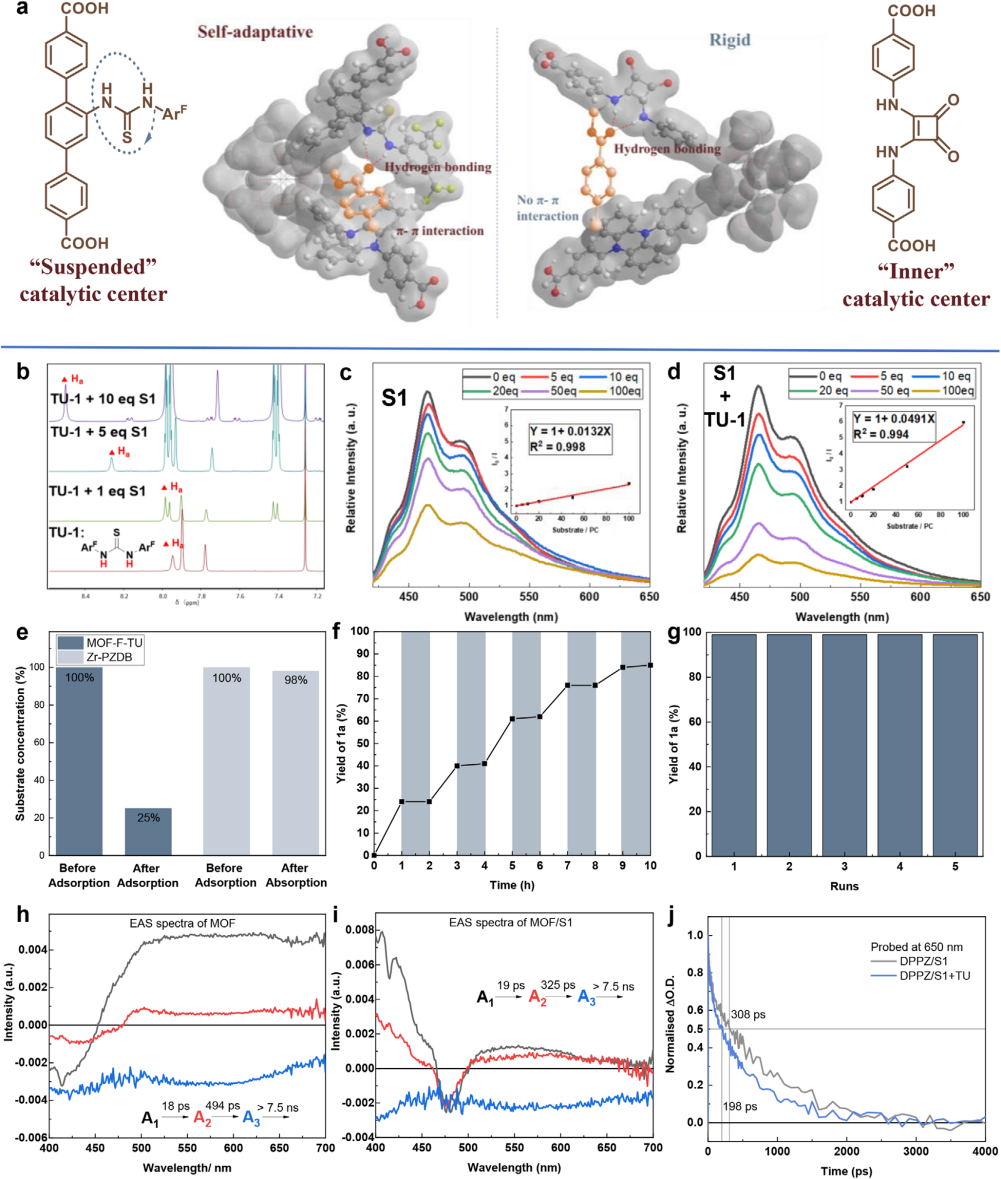
Mechanistic studies. (a) Computed activating models of self‐adaptive (left) and rigid (right) pockets in MOFs. (b) ^1^H NMR titration of TU‐1 with **S1** (25°C, 0.02 M TU‐1 in chloroform‐*d*). (c and d) Fluorescence quenching experiments of DPPZ by **S1** (c) or by a mixture of **S1** and TU‐1 (1:1) (d) inset: the corresponding Stern–Völmer curve. (e) Adsorption tests of **S1** by **MOF‐F‐TU** and **Zr‐PZDB**. (f) Yields of **1a** for light on/off experiments, period of light or darkness: 1 h. (g) Recycle test, yields of **1a** in 5 runs. (h and i) Three compartment sequential pathway global lifetime analysis (GLA) of (h) **MOF‐F‐TU** and (i) **MOF‐F‐TU**/**S1**. (j) Kinetic traces probed at 650 nm assigned to singlet excited state of DPPZ in the presence of **S1** or a mixture of **S1** and **TU**.

Mechanistically, we hypothesized that the DHBD center could engage with **S1** via hydrogen bonding, making the latter easier to be reduced. To verify this hypothesis, several experiments were designed and conducted. First, ^1^H NMR titration experiments revealed a continuing and downfield shift of the N─H signal in TU‐1 upon the addition of **S1** (Figure 3b). Second, adsorption experiments further confirmed that the interaction between **S1** and **MOF‐F‐TU** mainly originated from the TU sites, because **MOF‐F‐TU** effectively adsorbed 75% of **S1** while **Zr‐PZDB** without TU centers displayed little adsorption (Figure 3e). Finally, fluorescence quenching experiments were performed to demonstrate the influence of double hydrogen bonding on the photocatalytic process. Given the weak emission intensity of L‐PZDB‐Me, 5,10‐diphenyl‐5,10‐dihydrophenazine (DPPZ) was prepared as a model photosensitizer. Substrate **S1** quenched the excited DPPZ with a *K*_sv_ of 0.0132 (Figure 3c), while the addition of TU‐1 significantly increased the *K*_sv_ to 0.0491 (Figure 3d), marking a 3.7‐fold higher quenching rate. Control experiments using TU‐1 and DIPEA as quenchers showed no effect on excited DPPZ (Figure S57). These observations matched well with the catalytic performance, that a combination of TU‐1 and DPPZ as the catalyst resulted in a higher efficiency (56%) than DPPZ alone (22%). To clarify the excited‐state dynamics and relaxation pathways, we performed femtosecond transient absorption (fs TA) spectroscopy of **MOF‐F‐TU** both in the absence and presence of **S1** (Figure S58). Global analysis using a three‐component sequential model resolved an initially populated singlet excited state A_1_ (*τ*_1_ = 18 ps), followed by vibrational/structural relaxation A_2_ (*τ*_2_ = 494 ps) and a longer‐lived component A_3_ with a lifetime beyond our detection window. At longer delay times, the spectra evolved into a broad negative band across the visible region, consistent with the slowly recovering ground‐state bleach (Figure 3h). This long‐lived component reflects the slow decay of the excited states, mimicking the behavior observed in molecular photosensitizer DPPZ (Figure S59). Their overall relaxation pathways are similar, along with slightly different kinetics due probably to the confinement within the framework. Upon **S1** addition, the spectral evolution changed markedly (Figure 3i). A new negative peak centered at ∼470 nm appeared at initial timescale, which was also observed in the TA spectra of DPPZ/**S1** system (Figure S59). Meanwhile, the lifetime of A_2_ (relaxed singlet excited state) shortened from 494 to 325 ps (Figure 3h vs. i), indicating efficient quenching of A_2_ by **S1**. These results suggest ultrafast photoinduced charge transfer between the MOF's PZDB center and **S1**. On the other hand, the addition of **S1** reduced the excited‐state lifetime of DPPZ from 10 ns to 444 ps, while using a mixture of **S1** and TU‐1 further decreased the lifetime to 286 ps (Figure 3j). These results indicate the cooperative interaction between **S1** and TU‐1, that promotes the quench of excited DPPZ.

DFT/semi‐empirical computation was then carried out to simulate noncovalent interactions in MOF pockets (Figures 3a and S43–S48). As expected, **S1** was computed to form double hydrogen bonds with the DHBD sites in MOFs. Upon bonding, the relatively flexible DHBDs of **MOF‐F‐TU**, **MOF‐F‐U** and **MOF‐F‐SQA** can further adjust their conformations to create a suitable position for the π–π interaction between activated **S1** and PZDB, that effectively facilitates their single electron transfer after light irradiation. Conversely, the relatively rigid DHBD sites in **MOF‐R‐SQA1** and **MOF‐R‐SQA2** result in a longer distance and orientation mismatch between activated **S1** and PZDB, therefore prohibiting the above dual activation. These results underscore the importance of spatial arrangement in heterogeneous cooperative catalysis. The proper arrangement of catalytic centers can markedly improve catalytic performance, while the mismatched setup may even cause losing catalytic activity.

**FIGURE 4:**
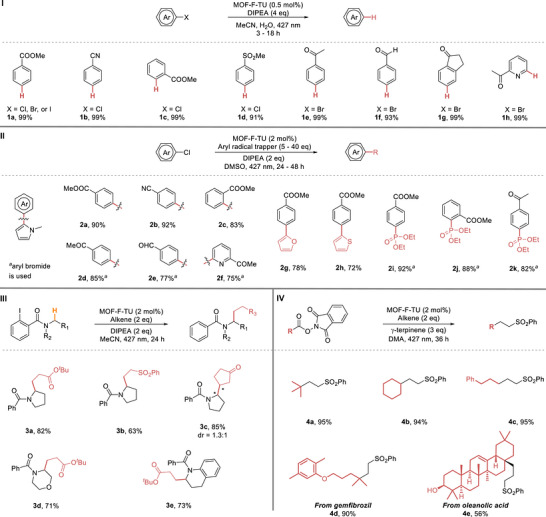
**MOF‐F‐TU** catalyzed organic transformations.

We selected **MOF‐F‐TU** as the optimal catalyst to investigate the scope of dehalogenation (Figure 4). By employing *N*, *N*‐diisopropylethylamine (DIPEA) as an electron sacrificial reagent, dechlorination of **S1** was completed within 18 h. Various substituents including ester, cyano, sulfonyl, acyl, and aldehyde on the phenyl ring were tolerated (**1a–1h**). Among them, the unsaturated functional groups remained untouched during the photoreduction. Pyridine‐containing substrate worked well to give **1h** quantitatively. We also explored the dehalogenative cross‐coupling by using *N*‐methyl pyrrole, furan, thiophene and triethyl phosphite as the aryl radical trapper. A series of aryl halides were compatible, yielding the target cross‐coupling products **2a–2k** in good to excellent isolated yields. Homogeneous control using a mixture of L‐PZDB‐Me and TU‐1 only afforded trace amounts of coupling products. Further analysis of the crude mixture revealed that L‐PZDB‐Me and TU‐1 decomposed under reaction conditions (Figures S61–S62), which might account for the deactivation of the homogeneous catalytic system.

We envisioned that the aryl radical, originating from **MOF‐F‐TU** photoreduction, might undergo intramolecular hydrogen‐atom transfer (HAT) to activate a remote C─H bond for realizing nonlocal C─H functionalization [100, 101]. With DIPEA as an electron source, the proposed HAT occurred to convert the aryl radical to an alkyl radical, which subsequently reacted with alkenes to deliver **3a–3e**. **MOF‐F‐TU** also effectively catalyzed the reductive cross‐coupling between *N*‐hydroxyphthalimide (NHPI) ester and alkene [102]. Upon forming double hydrogen bonds with the TU site, NHPI esters were activated and subsequently subjected to photoreduction by the excited PZDB center. The resulting alkyl radical attacked olefins to afford **4a–4c** in excellent yields. Notably, substrates derived from complex bioactive molecules such as *gemfibrozil* and *oleanolic* acid were also compatible (**4d** and **4e**).

To demonstrate the practical application potential of **MOF‐F‐TU**, the gram‐scale experiment was conducted (Figure S41). Dechlorination of **S1** proceeded smoothly with an exceptionally low catalyst loading (0.01 mol%), delivering **1a** in 95% yield (1.29 g) with a high TON (9500) and TOF (132 h^−1^) (Figure S42). Moreover, **MOF‐F‐TU** could be conveniently recovered and reused as the catalyst in five successive runs of dechlorination without activity decline (Figure 3g). After five cycles of the catalytic reaction, **MOF‐F‐TU** maintained its crystalline integrity, as evidenced by the consistent PXRD pattern (Figure 2b). To validate the heterogeneous nature of **MOF‐F‐TU** catalysis, hot filtration experiments were conducted. No substantial yield improvement was observed after removing the MOF catalyst (Figure S63). Inductively coupled plasma mass spectrometry (ICP‐MS) analysis of the supernatant from the reaction mixture revealed a Zr leaching level of less than 0.4%, suggesting the stability of **MOF‐F‐TU** under the reaction conditions.

Several additional control experiments were performed to illustrate the reaction mechanisms. The results of light on–off experiments for **S1** dehalogenation revealed that almost no reaction happened in the dark (Figure 3f), negating the radical chain pathway. The addition of 2,2,6,6‐tetramethylpiperidinooxy (TEMPO), a commonly used radical capture, to the reaction mixture markedly inhibited the reactions. The radical‐capture species were also identified by high‐resolution mass spectrometry (HRMS). Electron paramagnetic resonance (EPR) experiments detected the signal of *C*‐phenyl‐*N*‐tert‐butylnitrone (PBN)‐trapped aryl radical species. However, no EPR signal was detected in the absence of light, catalyst, substrate, or PBN (radical trapper, Figure S53). These results suggesting the presence of aryl or alkyl radicals in the chemical transformations (Figures S50–S53).

Based on the experimental results and literature precedents, a general reaction mechanism was proposed (Figure S64). Upon light irradiation, single electron transfer occurs from the excited PZDB linker to the substrate activated by double hydrogen bonding with DHBD sites. The reduced substrates then undergo dehalogenation to form an aryl radical or decarboxylation to generate an alkyl radical. Subsequently, the aryl radical either abstracts a hydrogen atom from DIPEA or attacks aryl radical trapper to yield the corresponding products **1** or **2**, respectively. Alternatively, the aryl radical can undergo intramolecular 1,5‐HAT to activate a distant sp^3^ C─H bond, giving an alkyl radical. This alkyl radical, as well as the one generated from decarboxylation of NHPI ester, are then trapped by electron‐deficient alkenes to yield products **3** and **4**.

## 3 Conclusion

We have created self‐adaptive pockets equipped with both photocatalytic and double hydrogen bond donor centers in **MOF‐DHBDs**. Catalytic performance evaluation of these MOFs underscores the dominant role of flexibility and spatial orientation of synergistic catalytic centers, that is even more important than the activity of catalytic center. Mechanistic studies substantiate the double hydrogen‐bonding interaction between DHBD sites and substrates. Moreover, DFT/semi‐empirical computation indicates a conformational adjustment after hydrogen bonding, which allows for a π–π overlapping of activated substrates with the PZDB center. This cooperative activation significantly promotes the photoreduction of the substrate and thereby induces chemical transformations. The optimal **MOF‐F‐TU** displayed versatile catalytic capability in dehalogenation, dehalogenative arylation, remote C─H functionalization, and decarboxylative alkylation with turnover numbers of up to 9500. Its superior performance can be attributed to self‐adaptive catalytic environment, matched spatial arrangement of active centers, and site‐separation to avoid aggregation‐induced activity declines. The results of this study demonstrate the importance of spatial arrangement of cooperative catalytic centers in heterogeneous catalysis, which might inspire the design of other heterogeneous synergistic catalysis.

## 4 Experimental Section/Methods

### 4.1 Synthesis of MOF

ZrCl_4_ (42 mg) and HCl (16 µl, 37%) were mixed in 2 mL of dimethylformamide (DMF) and sonicated until being fully dissolved. Then, L‐PZDB‐H (25.3 mg, 0.06 mmol), L‐NH_2_‐H (40 mg, 0.12 mmol), and L‐proline (103 mg) were mixed in 2 mL of dimethylformamide (DMF) and sonicated for 10 min. The prepared two solutions were mixed and sonicated for 1 min. Later, the resulting yellow suspension was heated in an oven at 100^0^C for 48 h, followed by cooling to room temperature to afford yellow octahedron crystals.

### 4.2 General Procedure for Dehalogenation

In a N_2_‐filled glovebox, aryl halide (0.2 mmol), N,N‐diisopropylethylamine (DIPEA, 130.4 mg, 0.8 mmol), ***MOF‐DHBD*** (0.5 mol% based on the PZDB site), and MeCN (2 mL) were loaded into a 10‐mL reaction tube equipped with a magnetic stirring bar. The tube was then taken out from the glovebox, to which was added 0.2 mL degassed H_2_O. After being tightly screw‐caped, and the reaction mixture was subsequently stirred under irradiation (Kessil PR160L‐427) at room temperature for 16 h. GC‐MS analysis of the crude reaction mixture was performed to identify the yield of the product.

## Author Contributions

**Jing Ouyang**: writing – original draft, investigation, and conceptualization. **Sze Lam Lam**: investigation. **Chao Li**: investigation. **Peiqi Zhang**: investigation. **Le Yang**: investigation. **Zhiyi Yang**: investigation. **Teng‐Teng Chen**: investigation. **Xueliang Xiao**: writing – review and editing. **Yangjian Quan**: conceptualization and writing – review and editing.

## Conflicts of Interest

The authors declare no conflicts of interest.

## Supporting information




**Supporting File**: anie73086‐sup‐0001‐SuppMat.pdf

## Data Availability

The data that supports the findings of this study are available in the Supporting Information of this article.
