# Supplementary material for: Spatial Orientation‐Dependent Double Hydrogen Bond/Photoredox Cooperative Catalysis in Metal–Organic Frameworks
Source: Angew Chem Int Ed Engl. 2026 Jun 7;65(32):e5181076. doi: 10.1002/anie.5181076 (PMC13427173; doi:10.1002/anie.5181076)
Supplement: Supplementary file 1 — Supporting File: anie73086‐sup‐0001‐SuppMat.pdf [file ANIE-65-e5181076-s001.pdf]

Supporting Information

Spatial orientation-dependent double hydrogen bond/photoredox cooperative catalysis in metal-organic frameworks

Jing Ouyang,[1] Sze Lam Lam,[1] Chao Li,[1] Peiqi Zhang,[1] Le Yang,[1] Zhiyi Yang,[1] Teng-Teng Chen,[1] Xueliang Xiao[2] and Yangjian Quan[1]*

((Optional Dedication))

[1]Department of Chemistry, The Hong Kong University of Science and Technology (HKUST), Clear Water Bay, Kowloon, Hong Kong SAR (China)
[2]Key Lab of Special Protective Textiles, Minstry of Education, Jiangnan University, Wuxi, China
E-mail: chyjquan@ust.hk

1. General Procedure	S2
2. Synthesis and Characterization of MOF	S3
3. Catalytic Reactions	S40
4. Mechanistic Study	S48
5. GC-MS Spectra	S70
6. NMR Spectra	S73
7. Reference	S96

1. General Procedure

Staring materials, reagents and solvents were purchased from commercial sources and used without further purification. Infrared spectra in the range 400–4000 cm^{-1} were recorded on a Nicolet Avatar 360 FT-IR spectrophotometer. Solution ^{1}H NMR and ^{13}C NMR spectra were recorded at room temperature on Bruker superconducting-magnet high-field NMR spectrometers with working frequencies of 400 and 600 MHz. Chemical shifts (δ) are expressed in ppm relative to the residual solvent (e.g., chloroform ^{1}H: 7.26 ppm) reference. Coupling constants are expressed in hertz. Thermogravimetric analyses (TGA) were carried out in an air stream using PerkinElmer Thermal Analysis equipment (STA 6000) with a heating rate of 15 °C/min, with an empty Al_2O_3 crucible being used as the reference. The porosity and surface area analyses were performed using a Quantachrome Autosorb iQ gas sorption analyzer. Samples were outgassed at 0.03 torr with a 5 °C/min ramp to 40 °C and held at 40 °C for 2 hours. The samples were then held in vacuum until the analysis was run. Pore analysis was performed using N_2 at 77.35 K (P/P_0 range of 2×10^{-7} to 0.995). Powder X-ray diffraction data was collected in the reflection mode at room temperature on a PANalytical X'Pert Pro powder X-ray diffractometer with a Cu source (λ = 1.54056 Å). The X-ray tube operated at a voltage of 40 kV and a current of 40 mA. The indexing of powder patterns was conducted using software of CELREF (Version 3). SEM images were taken on a TM3030 Tabletop Microscope. UV-vis absorption spectroscopic measurements were performed using a UH5700 spectrophotometer equipped with a sample tray filled with $BaSO_4$ (solid) or 60 mm diameter integrating sphere (liquid). Fluorescence measurements were performed using an EI-FS5 Fluoresce Spectrometer. Mass spectra were collected on an Agilent GC/MS 5975C system, a MALDI Micro MX mass spectrometer, or an API QSTAR XL System. Photocurrent results were collected on a VMP3 BioLogic electrochemical workstation. Kessil PR160L-427 LED lamps were used for reactions. The fs-ns transient absorption (TA) spectra were collected on a Helios Spectrometer (Ultrafast System). The pump light was generated using 90% laser source (Coherent, FWHM ~34 fs, 1 kHz, 800 nm) coupled to an optical

parametric amplifier (Ultrafast System) and an internal chopper lowers the pump frequency to 0.5 kHz to generate tunable wavelength pump light. A white light probe (10% laser source) beam was generated by focusing 800 nm output onto a CaF_2 crystal within the Helios spectrometer. Samples were excited with a 390 nm with a power of 200 μW. The pump and probe beam were overlapped on the sample position. Data were initially collected using the Helios TA spectrometer and analysed using Surface Xplorer software (Ultrafast System). The EPR spectra was collected on Bruker BioSpin GmbH (EMXplus).

2. Synthesis and Characterization of MOFs

2.1 Synthesis of Ligands

2.1.1 Synthesis of L-PZDB-H

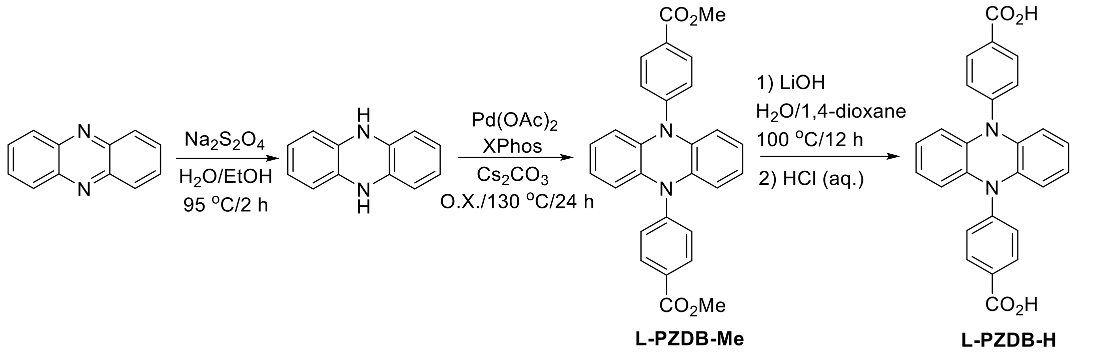

A solution of sodium dithionite (14.63 g, 84.0 mmol) in water (130 mL) was slowly added to a boiling solution of phenazine (1.08 g, 6.0 mmol) in EtOH (30 mL). The mixture was refluxed at 95 ℃ for 2 h. The resulting greenish gray precipitate was filtered off, washed by water, and dried under vacuum to give the **5,10-dihydrophenazine** (0.98 g) in 90% yield.

A mixture of **5,10-dihydrophenazine** (0.73 g, 4.0 mmol), methyl 4-bromobenzoate (1.89 g, 8.8 mmol), $Pd(OAc)_2$ (44.9 mg, 0.2 mmol, 5 mol%), Xphos (191 mg, 0.4 mmol, 10 mol%) and Cs_2CO_3 (2.61 g, 8.0 mmol) was added to a Schlenk flask under N_2 atmosphere. Then anhydrous *o*-xylene (50 mL) was added. The resultant mixture was stirred at 130 °C for 24 h. After cooling to room temperature, water (60 mL) was added,

and the organic phase was extracted with dichloromethane (DCM) (90 mL ×2). After the removal of organic solvent under vacuum, the residue was subjected to column chromatography to afford **L-PZDB-Me** (1.49 g) in 85% yield. ^{1}H NMR (600 MHz, Chloroform-d) δ 8.28 (s, 4H), 7.48 (s, 4H), 6.34 (s, 4H), 5.70 (s, 4H). ^{13}C NMR (151 MHz, Chloroform-*d*) δ 166.47, 144.63, 136.06, 132.78, 131.04, 129.89, 121.51, 113.26, 52.45. HRMS (ESI): m/z Calculated for $C_{28}H_{22}N_2O_4$ [M^+]: 450.1580, found 450.1582.

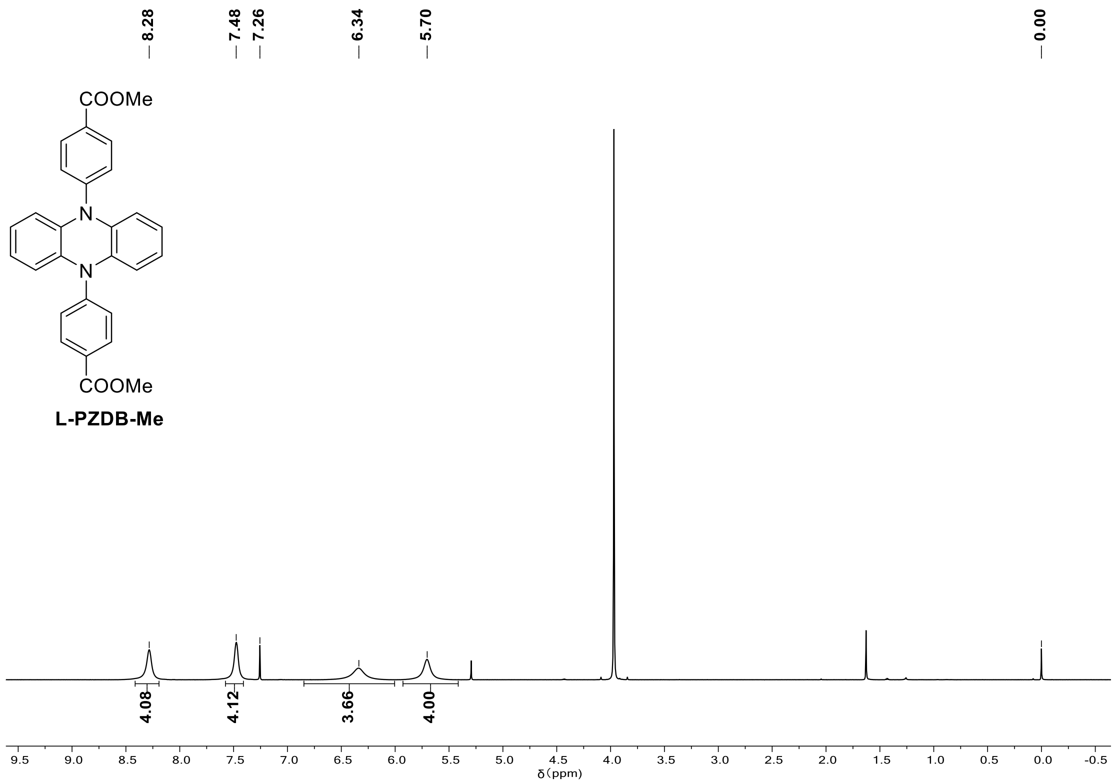

Figure S1. ^{1}H NMR spectrum (600 MHz) of **L-PZDB-Me** in $CDCl_3$.

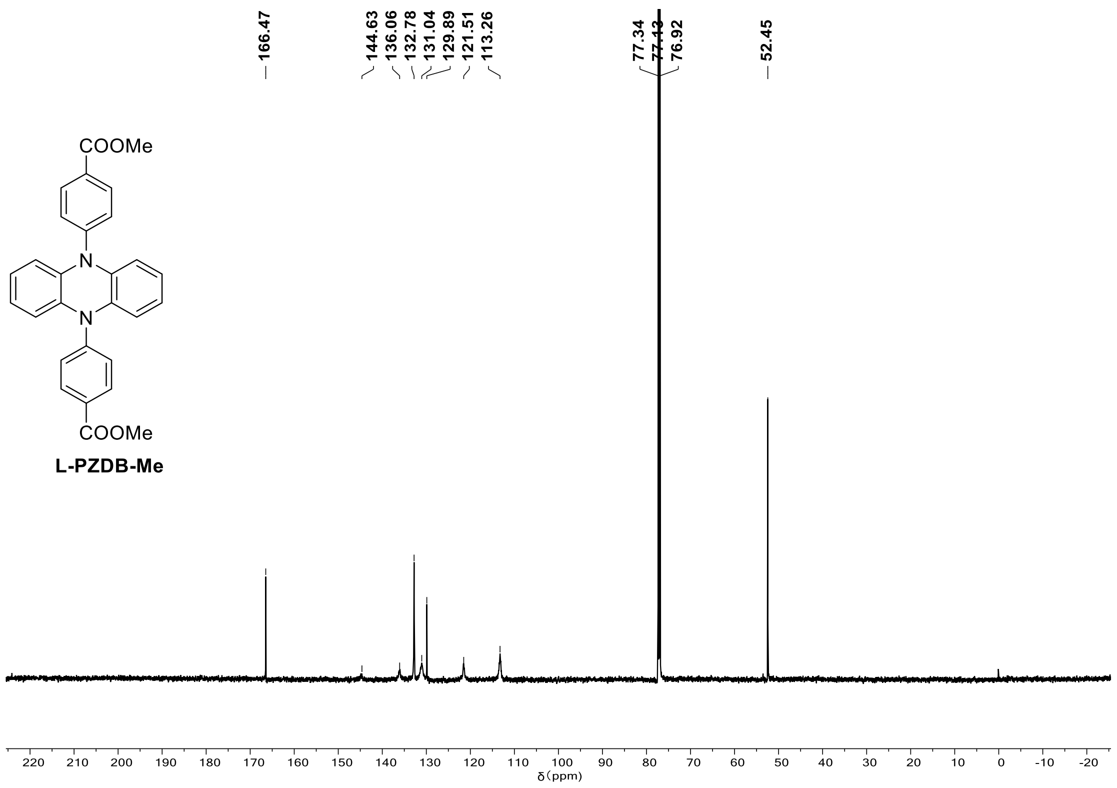

Figure S2. ^{13}C NMR spectrum (153 MHz) of **L-PZDB-Me** in $CDCl_3$.

A suspension of **L-PZDB-Me** (1.49 g, 3.0 mmol) and LiOH (0.72 g, 30.0 mmol) in dioxane (30 mL) and water (15 mL) was heated at 100°C for 12 h under dry N_2. After cooling down to room temperature, HCl aqueous solution was added to the reaction mixture until pH = 2. The yellow precipitate was filtered and washed by water, hexane, and dichloromethane to give **L-PZDB-H** (1.13 g) in 92 % yield. ^{1}H NMR (400 MHz, DMSO-d_6) δ 8.14 (d, J = 8.1 Hz, 4H), 7.36 (d, J = 8.2 Hz, 4H), 6.35 (m, 4H), 5.63 (dd, *m*, 4H). Due to the low solubility of **L-PZDB-H**, its ^{13}C NMR spectrum cannot be obtained. HRMS (ESI): m/z Calculated for $C_{26}H_{18}N_2O_4$ [M^+]: 422.1267, found 422.1267.

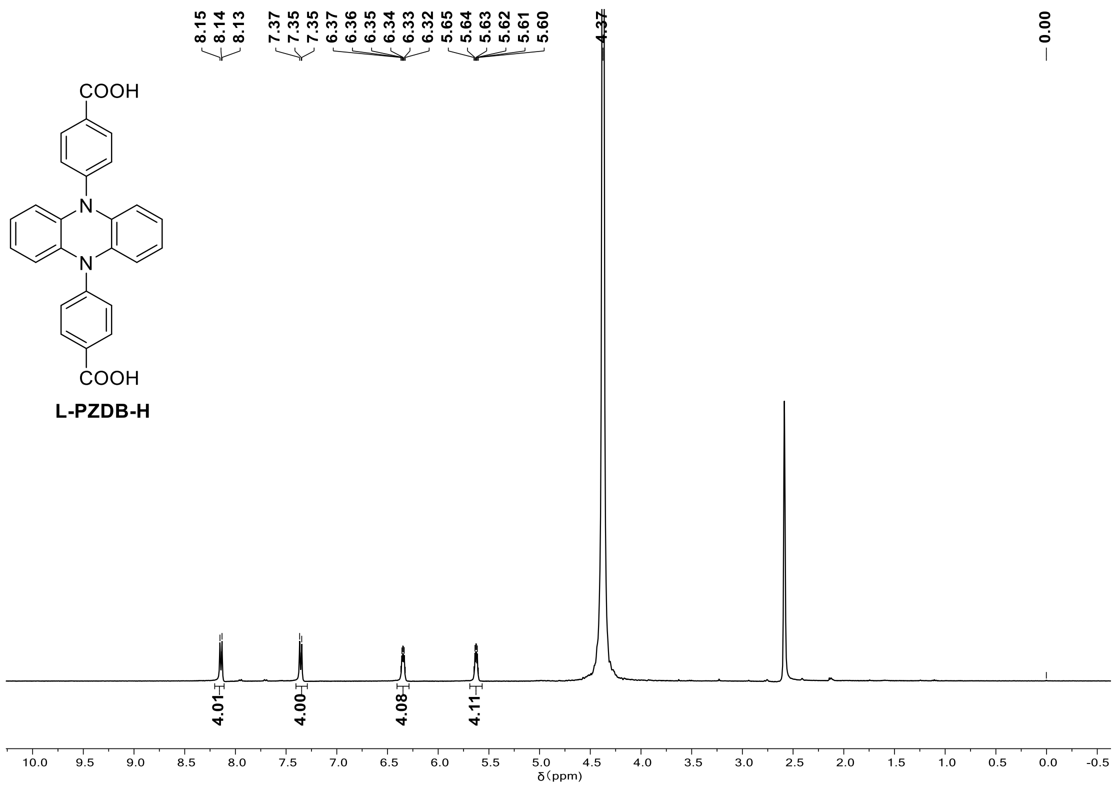

Figure S3. ^{1}H NMR spectrum (400 MHz) of **L-PZDB-H** in K_3PO_4/D_2O/DMSO-d_6.

2.1.2 Synthesis of L-NH_2-H

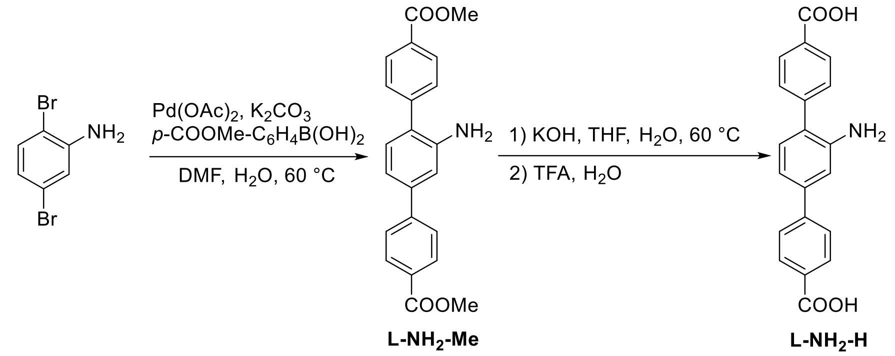

Ligand **L-NH_2-H** was prepared according to a reported procedure with minor modifications.[1] A solid mixture of 4-methoxycarbonylphenylboronic acid (11.52 g, 64.0 mmol), 2,5-dibromoaniline (3.00 g, 12.0 mmol), Na_2CO_3 (4.52 g, 42.6 mmol) and $Pd(OAc)_2$ (26.3 mg, 0.08 mmol) was added to a 100 mL round bottom flask. Then, a mixed solvent of 20 mL DMF and 10 mL H_2O was added. The resulting mixture was stirred and bubbled with nitrogen at room temperature for 30 minutes and then heated at 60°C for 24 hours. After reaction, the mixture was cooled to room temperature, to which was added water (100 mL). The organic portions were extracted with dichloromethane (100 mL * 3), combined, and dried under vacuum. The residue was purified by column chromatography (pure dichloromethane as the elute) to give a yellow solid **L-NH_2-Me** (3.37 g, 78% yield based on 2,5-dibromoaniline).

L-NH_2-Me (500 mg, 1.38 mmol), KOH (15.0 g), MeOH (50 mL), THF (100 mL) were sequentially added into a 250 mL round bottom flask. The mixture was stirred and bubbled with nitrogen at room temperature for 30 minutes and then heated at 60°C for 24 hours. After cooling to room temperature, the mixture was evaporated to 40 ml, to which was added 5 mL TFA. After stirring for 0.5 h, yellow precipitates were formed and then isolated by filtration. The solid was washed by H_2O (50 mL * 3) and DCM (20 mL * 3) and dried in vacuum to afford **L-NH_2-H** (410 mg, 90% yield based on **L-NH_2-Me**). ^{1}H NMR (400 MHz, DMSO-d_6) δ 8.05 (d, J = 8.2 Hz, 4H), 7.80 – 7.71 (m, 2H), 7.67 – 7.59 (m, 2H), 7.20 – 7.14 (m, 2H), 7.02 (m, 1H). ^{13}C NMR (101 MHz, DMSO-d_6) δ 167.76, 167.73, 146.28, 145.07, 144.32, 140.04, 131.39, 130.49, 130.37,

130.06, 129.62, 129.26, 127.04, 125.14, 116.07, 114.36. HRMS (ESI): m/z Calculated for $C_{20}H_{15}NO_4$ [M^+]: 333.1001, found 333.1007.

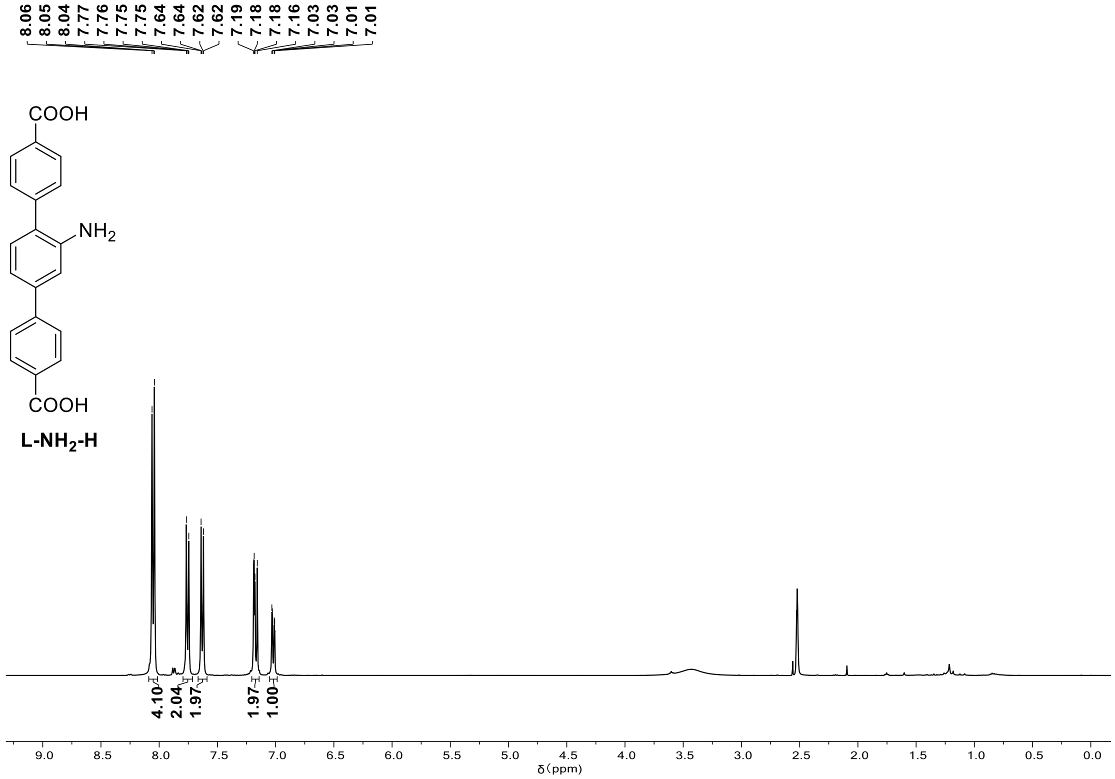

Figure S4. ^{1}H NMR spectrum (400 MHz) of **L-NH$_2$-H** in DMSO-d_6.

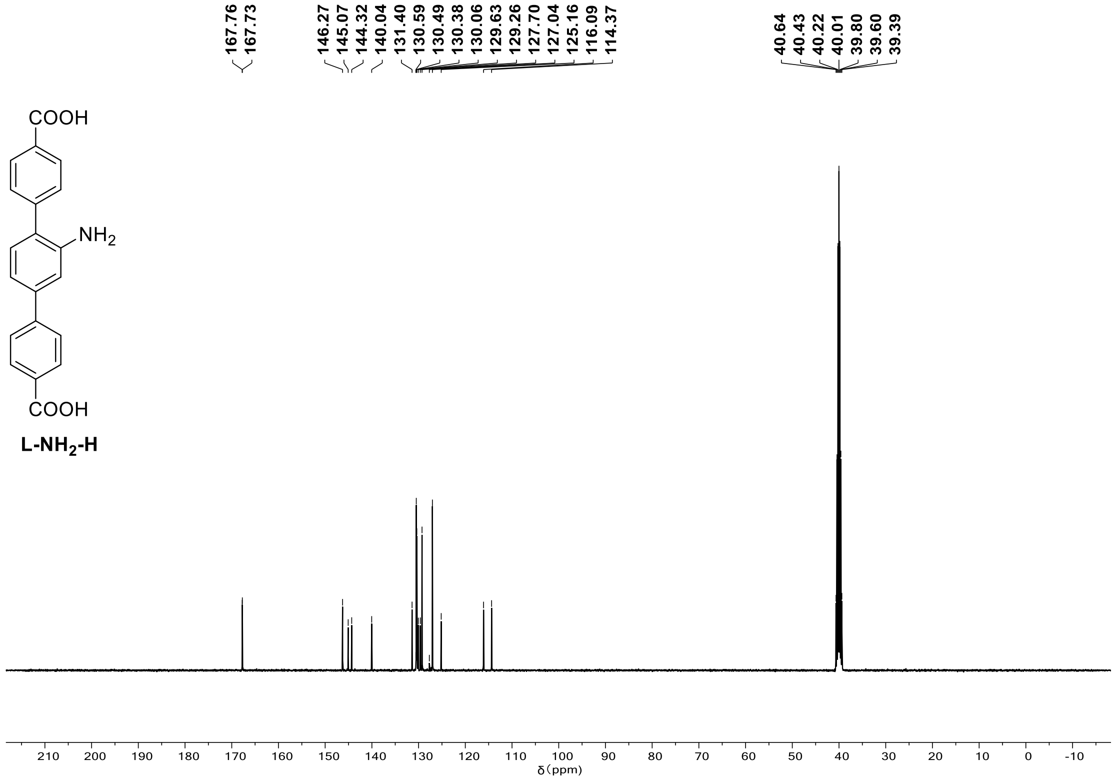

Figure S5. ^{13}C NMR spectrum (101 MHz) of **L-NH$_2$-H** in DMSO-d_6.

2.1.3 Synthesis of L-TU-H

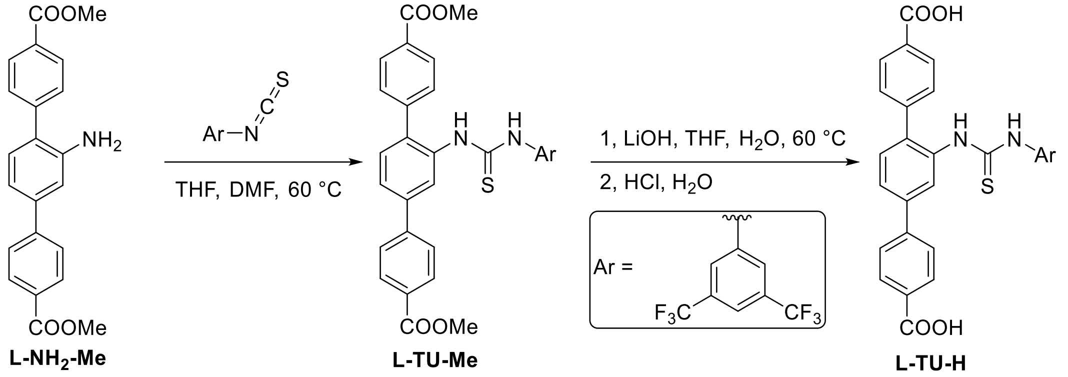

A mixture of **L-NH2-Me** (361 mg, 1.0 mmol), dry THF (10 mL) and dry DMF (2 mL) was stirred in a 50 mL round bottom flask for 0.5 h. Then, 3,5-trifluoromethylphenyl isothiocyanate (542 mg, 2.0 mmol) was slowly added to the mixture, followed by heating at 60°C for 24 h. The reaction mixture turned to a clear solution. After cooling down to room temperature and being evaporated on a rotary evaporator, the residue was then purified by column chromatography to give **L-TU-Me** (474 mg) in 75% yield. ^{1}H NMR (400 MHz, DMSO-d_6) δ 10.33 (s, 1H), 9.86 (s, 1H), 8.10 (m, 2H), 8.01 – 7.91 (m, 4H), 7.85 (m, 1H), 7.79 (m, 1H), 7.67 (d, J = 17.2 Hz, 2H), 7.59 (m, 4H), 3.88 (m, 6H). The low solubility of **L-TU-Me** prevented its characterization via ^{13}C NMR. HRMS (ESI): m/z Calculated for $C_{31}H_{22}F_6N_2O_4SNa$ [M+ Na^+]: 655.1097, found 655.1102.

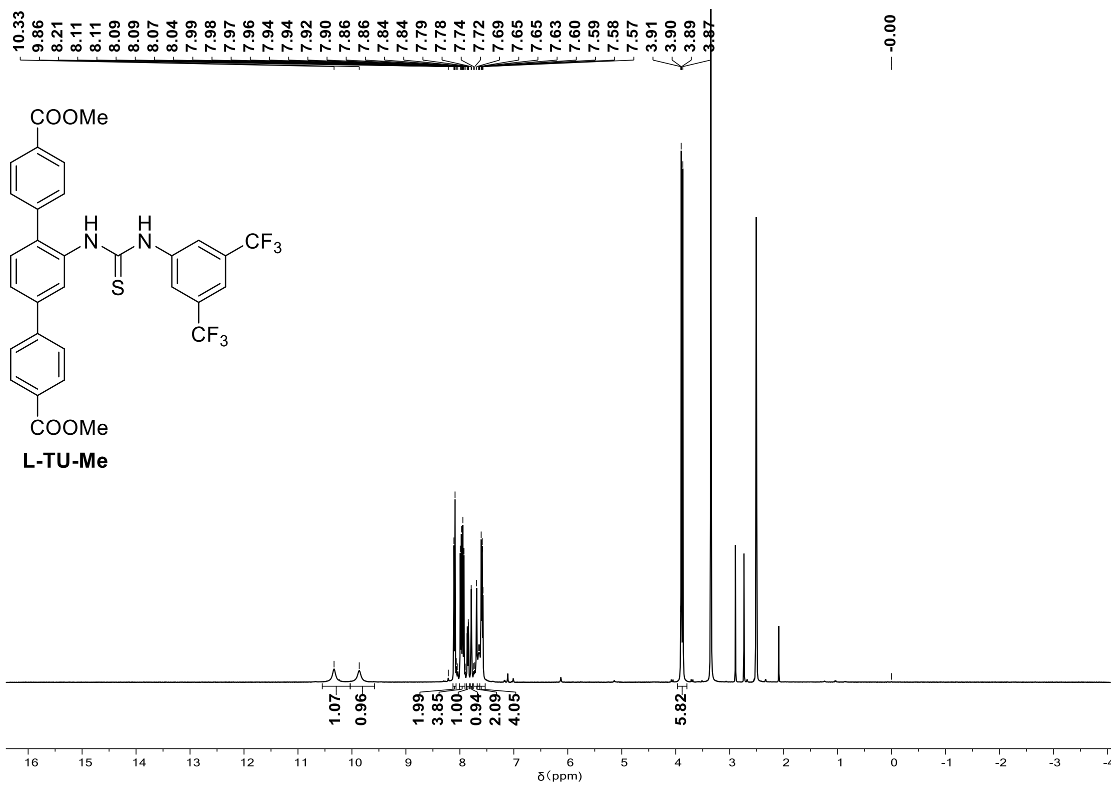

Figure S6. ^{1}H NMR spectrum (400 MHz) of **L-TU-Me** in DMSO-*d_6*.

A suspension of **L-TU-Me** (316 mg, 0.5 mmol) and LiOH (48 mg, 2 mmol) in THF (10 mL) and water (5 mL) was heated at 60°C for 12 h under dry N_2. After cooling down to room temperature, HCl aqueous solution was added to the reaction mixture until pH = 2. The white precipitate was filtered and washed by water, hexane, and dichloromethane to give **L-TU-H** (272 mg) in 90 % yield. ^{1}H NMR (600 MHz, DMSO-*d_6*) δ 10.23 (s, 1H), 9.81 (s, 1H), 8.03 (m, 3H), 7.92 (d, *J* = 7.9 Hz, 2H), 7.86 (d, *J* = 8.0 Hz, 2H), 7.80 (m, 1H), 7.73 (s, 1H), 7.68 – 7.62 (m, 2H), 7.53 (m, 3H). ^{13}C NMR (151 MHz, DMSO-*d_6*) δ 181.05, 167.78, 167.76, 146.14, 145.06, 144.28, 141.73, 140.08, 131.33, 130.93 (q, *J* = 33.9 Hz), 130.46, 130.36, 129.69, 129.20, 126.98, 125.25, 124.43, 123.66 (q, *J* = 277.2), 117.99, 116.15, 114.44. HRMS (ESI): m/z Calculated for $C_{29}H_{18}F_6N_2O_4SNa$ [M+ Na$^+$]: 627.0784, found 627.0788.

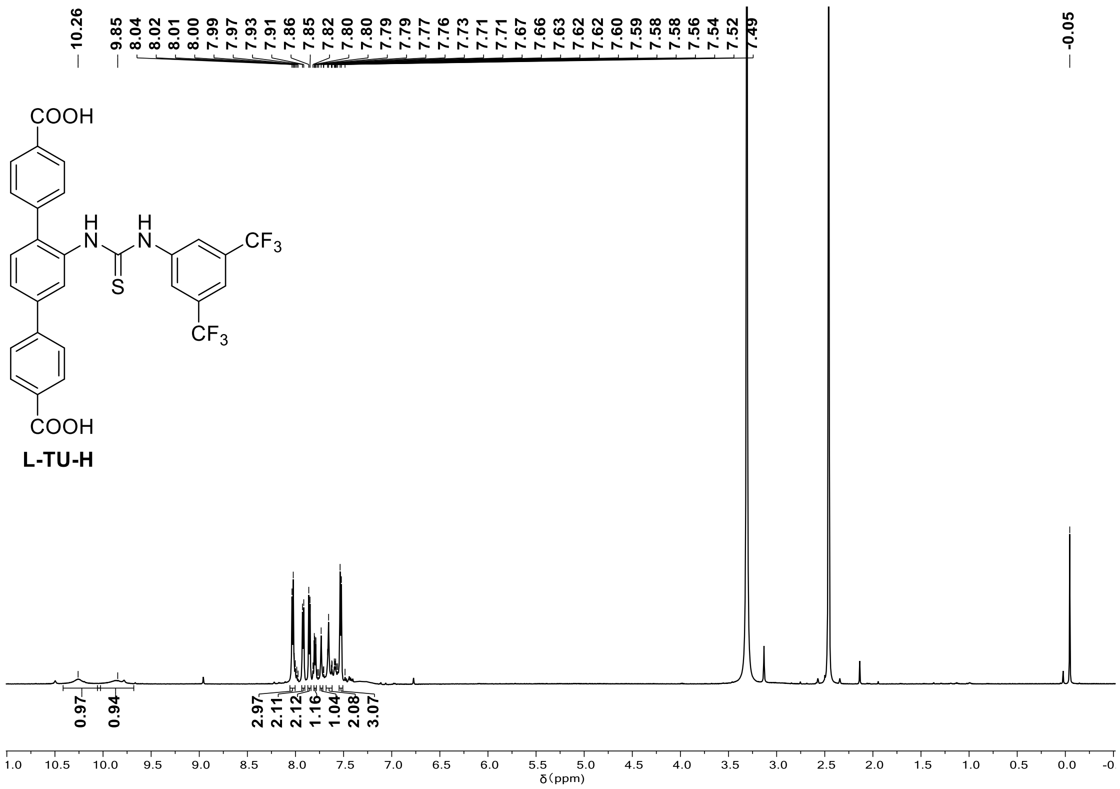

Figure S7. ^{1}H NMR spectrum (600 MHz) of **L-TU-H** in DMSO-d_6.

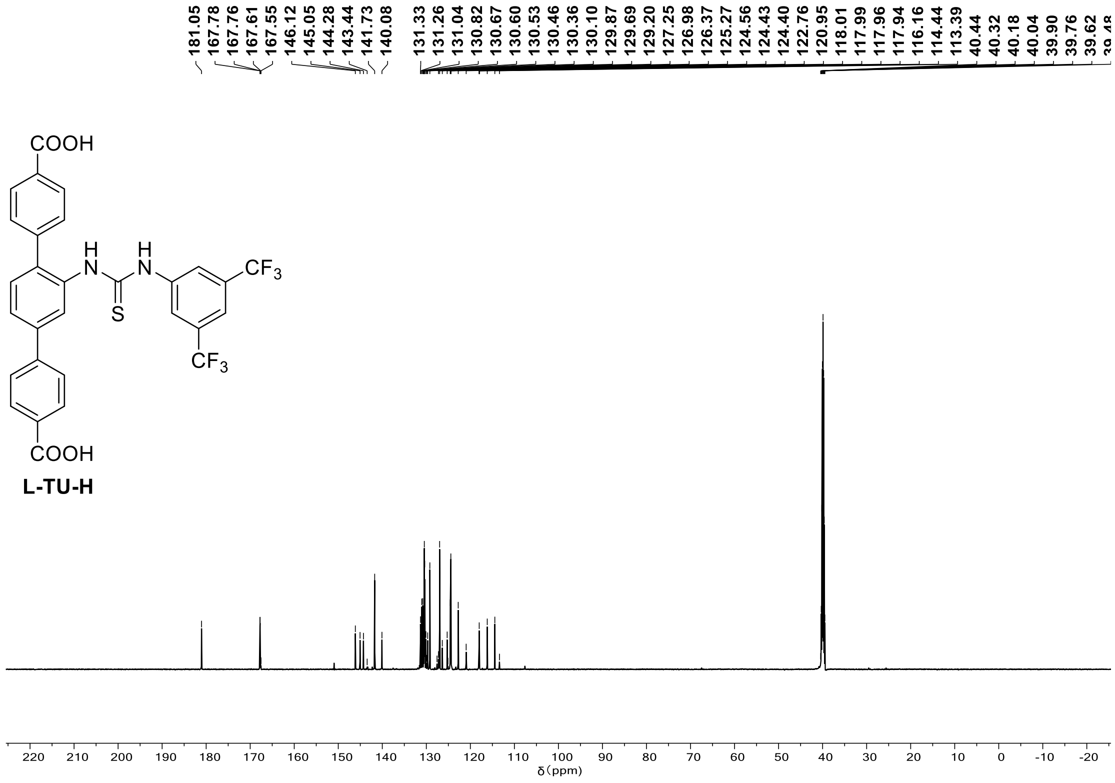

Figure S8. ^{13}C NMR spectrum (151 MHz) of **L-TU-H** in DMSO-d_6.

2.1.4 Synthesis of L-U-H

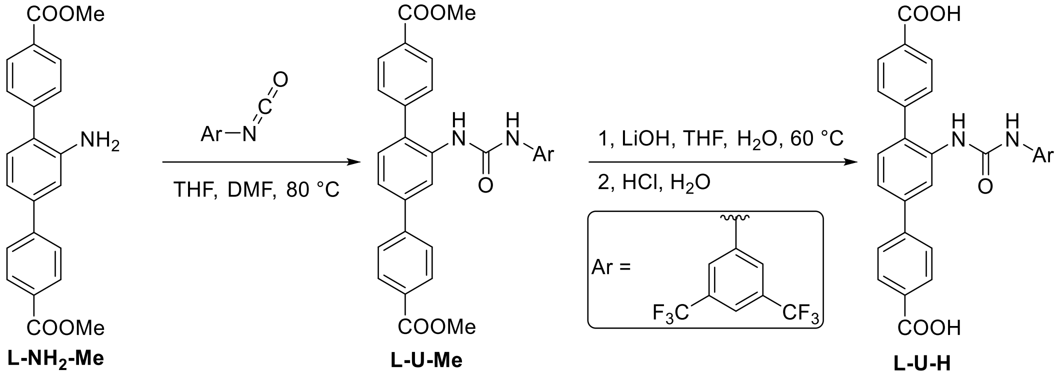

A mixture of **L-NH$_2$-Me** (361 mg, 1.0 mmol), dry THF (2 mL) and dry DMF (2 mL) was stirred in a 20 mL round bottom flask for 0.5 h. Then, 3,5-trifluoromethylphenyl isocyanate (510 mg, 2.0 mmol) was slowly added to the mixture, followed by heating at 80°C for 24 h. The reaction mixture turned to a clear solution. After cooling down to room temperature and being evaporated on a rotary evaporator, the residue was then purified by column chromatography to give **L-U-Me** (450 mg) in 74% yield. ^{1}H NMR (600 MHz, Chloroform-d) δ 8.16 – 8.10 (m, 4H), 7.97 (s, 2H), 7.83 (s, 1H), 7.68 (d, J = 8.2 Hz, 2H), 7.63 – 7.57 (m, 2H), 7.24 (d, J = 7.8 Hz, 1H), 7.11 (m, 1H), 7.03 (d, J = 1.8 Hz, 1H), 3.96 (s, 3H), 3.95 (s, 3H). Due to the low solubility of **L-U-Me**, its ^{13}C NMR spectrum cannot be obtained. HRMS (ESI): m/z Calculated for $C_{31}H_{22}F_6N_2O_5$ [M$^+$]: 616.1433, found 616.1432.

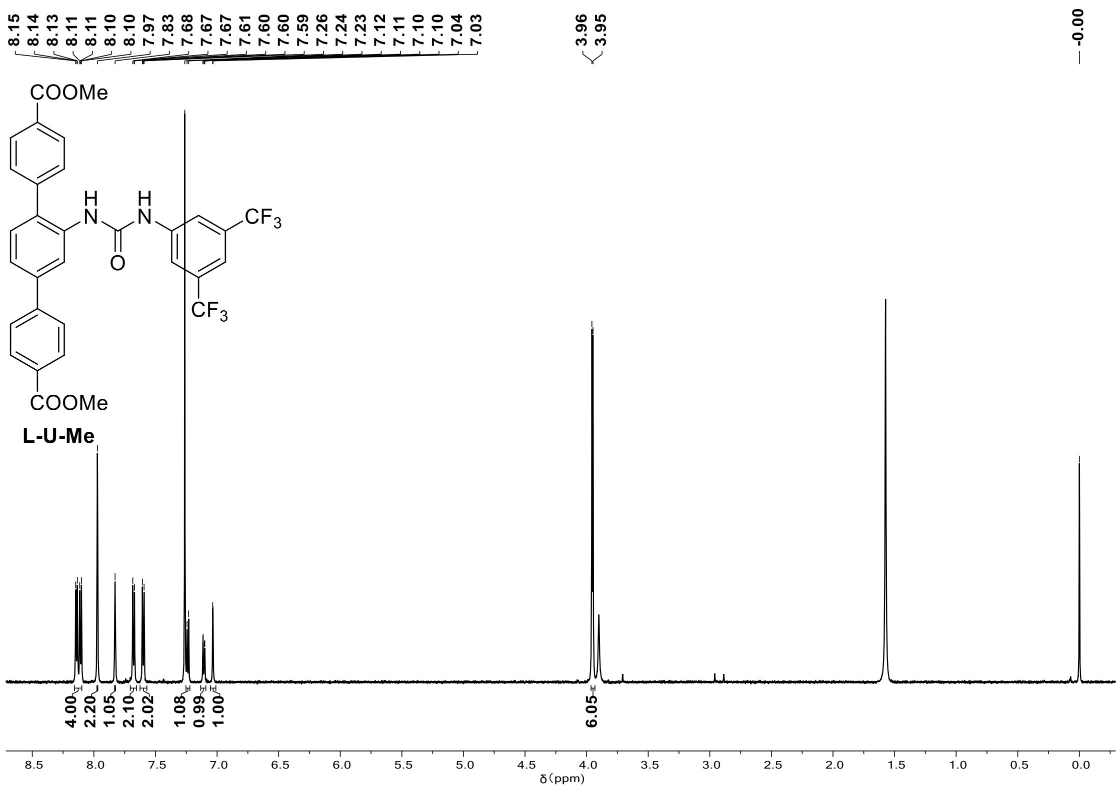

Figure S9. ^{1}H NMR spectrum (600 MHz) of **L-U-Me** in $CDCl_3$.

A suspension of **L-U-Me** (316 mg, 0.5 mmol) and LiOH (48 mg, 2 mmol) in THF (10 mL) and water (5 mL) was heated at 60°C for 12 h under dry N_2. After cooling down to room temperature, HCl aqueous solution was added to the reaction mixture until pH = 2. The white precipitate was filtered and washed by water, hexane, and dichloromethane to give **L-U-H** (272 mg) in 90 % yield. ^{1}H NMR (600 MHz, DMSO-*d_6*) δ 8.39 (s, 2H), 8.18 (s, 1H), 8.07 – 8.01 (m, 4H), 7.78 – 7.72 (m, 2H), 7.68 – 7.58 (m, 2H), 7.19 – 7.13 (m, 2H), 7.02 (m, 1H). Due to the low solubility of **L-U-H**, its ^{13}C NMR spectrum cannot be obtained. HRMS (ESI): m/z Calculated for $C_{29}H_{18}F_6N_2O_5$ [M^+]: 588.1120, found 588.1118.

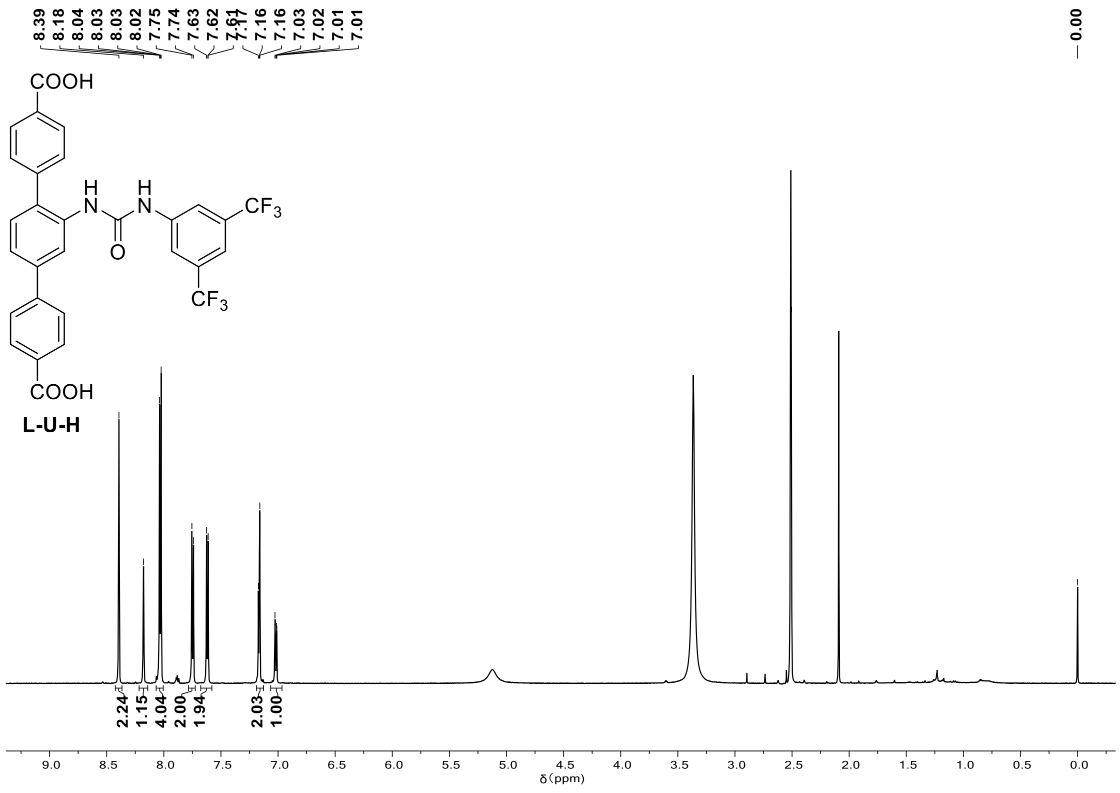

Figure S10. ^{1}H NMR spectrum (600 MHz) of **L-U-H** in DMSO-d_6.

2.1.5 Synthesis of L-SQA-R1-H

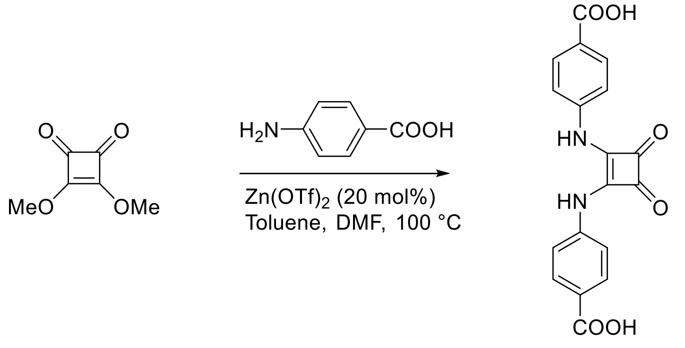

L-SQA-R1-H

Ligand **L-SQA-R1-H** was prepared according to a reported procedure with minor modifications.[2] A mixture of 4-aminobenzoic acid (4.11 g, 30.0 mmol), dimethyl squarate (1.42 g, 10.0 mmol) and $Zn(OTf)_2$ (0.72 g, 2.0 mmol) in 40 mL dry toluene and 2 mL dry DMF was heated to 100 °C for 24 hours. Precipitates were formed and isolated by filtration. The obtained solid was dispersed in boiled methanol (20 mL) for 5 minutes and then collected via filtration. Such a washing procedure was repeated two more times to afford **L-SQA1-R1-H** (2.74 g) in 78% yield. ^{1}H NMR (400 MHz,

DMSO-d_6) δ 10.16 (s, 2H), 7.90 – 7.86 (m, 4H), 7.50 (d, J = 8.6 Hz, 4H). ^{13}C NMR (101 MHz, DMSO-d_6) δ 182.57, 167.32, 166.41, 142.78, 131.42, 125.66, 118.49. HRMS (ESI): m/z Calculated for $C_{18}H_{12}N_2O_6Na$ [M+ Na$^+$]: 375.0588, found 375.0591.

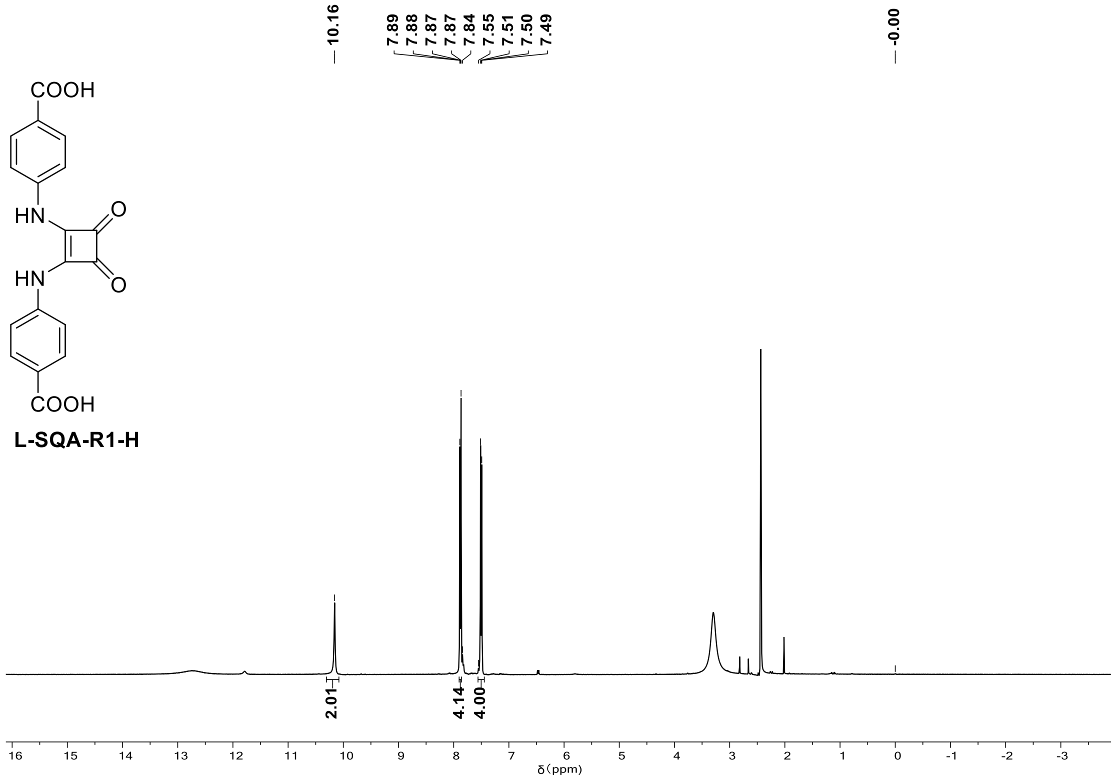

Figure S11. ^{1}H NMR spectrum (400 MHz) of **L-SQA-R1-H** in DMSO-d_6.

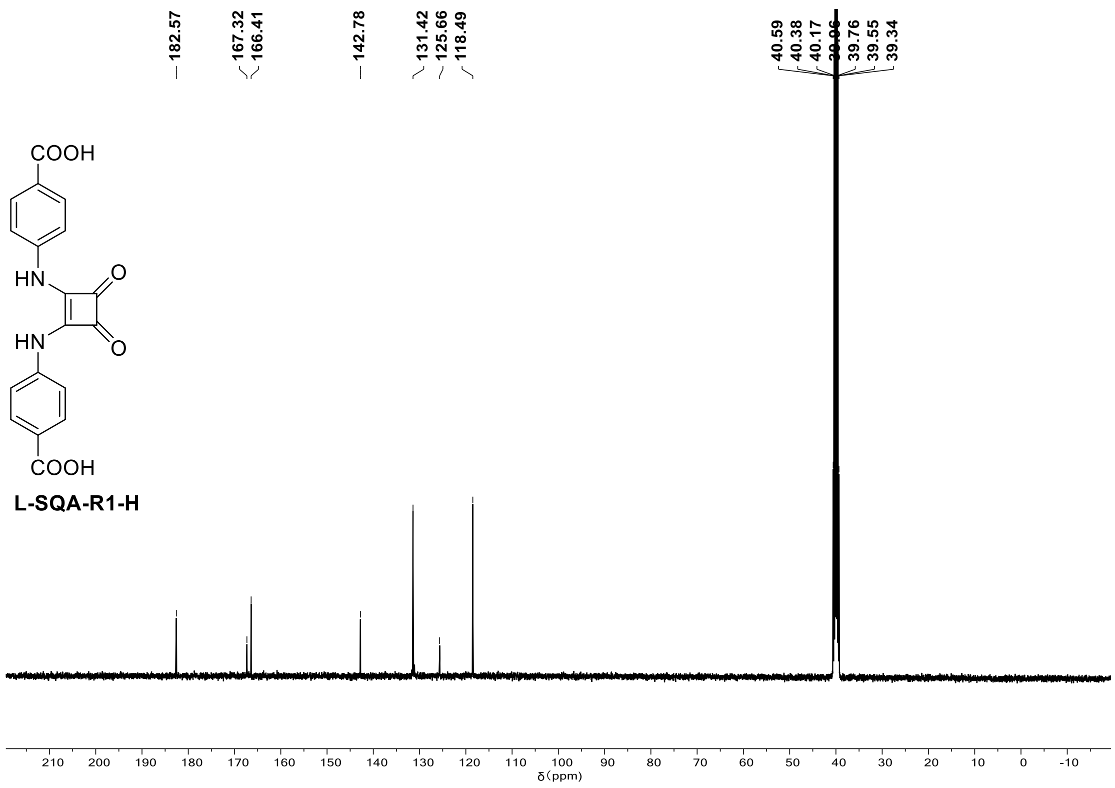

Figure S12. ^{13}C NMR spectrum (101 MHz) of **L-SQA-R1-H** in DMSO-d_6.

2.1.6 Synthesis of L-SQA-R2-H

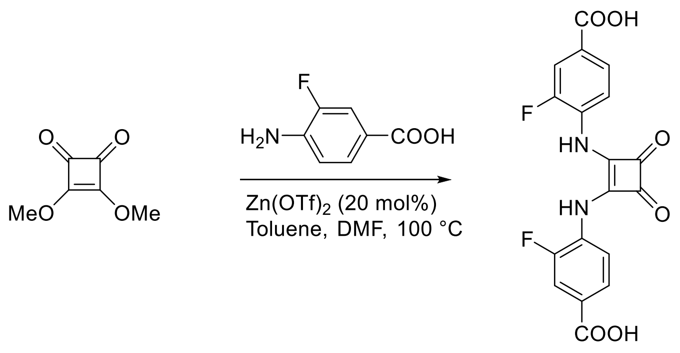

Ligand **L-SQA-R2-H** was prepared according to a reported procedure with minor modifications.[2] A mixture of 4-amino-3-fluorobenzoic acid (4.65 g, 30.0 mmol), dimethyl squarate (1.42 g, 10.0 mmol) and $Zn(OTf)_2$ (0.72 g, 2.0 mmol) in 40 mL dry toluene and 2 mL dry DMF was heated to 100 °C for 24 hours. Precipitates were formed and isolated by filtration. The obtained solid was dispersed in boiled methanol (20 mL) for 5 minutes and then collected via filtration. Such a washing procedure was repeated two more times to afford **L-SQA-R2-H** (2.94 g) in 80% yield. δ ^{1}H NMR (600 MHz,

DMSO-d_6) δ 10.47 (s, 2H), 8.02 (t, *J* = 8.6 Hz, 2H), 7.81 – 7.76 (m, 4H). Due to the low solubility of **L-SQA-R2-H**, its ^{13}C NMR spectrum cannot be obtained. HRMS (ESI): m/z Calculated for $C_{18}H_{10}F_2N_2O_6Na$ [M+Na$^+$]: 411.0399, found 411.0395.

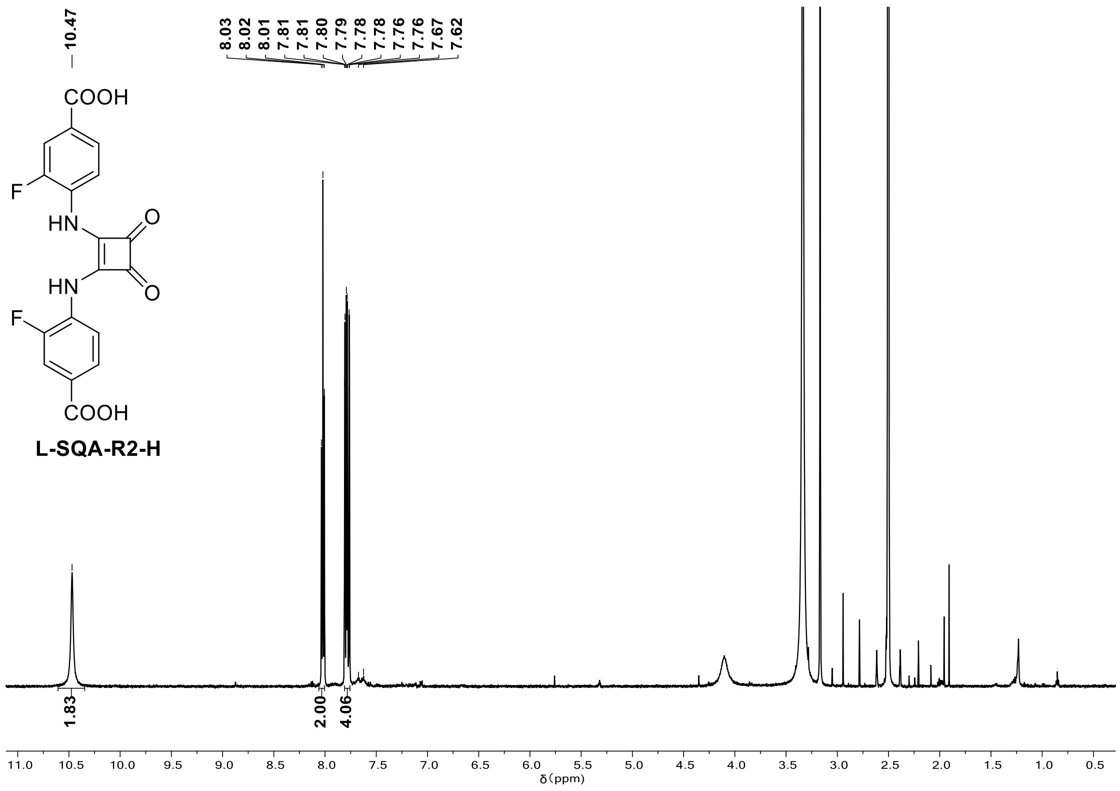

Figure S13. ^1H NMR spectrum (600 MHz) of **L-SQA-R2-H** in DMSO-d_6.

2.1.7 Synthesis of L-SQA-F-H

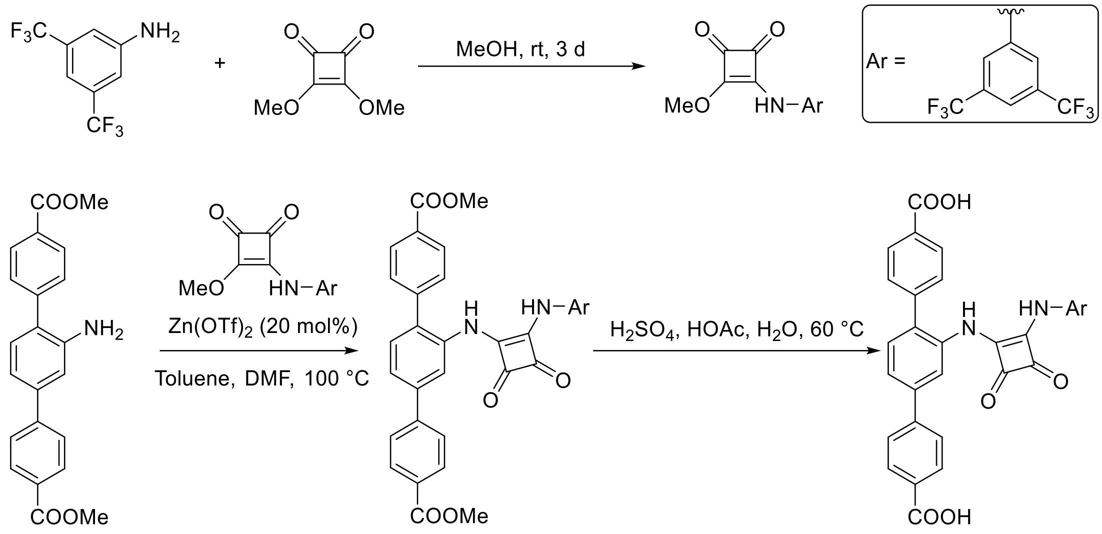

L-NH$_2$-Me **L-SQA-F-Me** **L-SQA-F-H**

Dimethyl squarate (1.42 g, 10.0 mmol) was dissolved in 20 mL of methanol. Then, 3,5-bis(trifluoromethyl)benzenamine (2.29g, 10.0 mmol) was added into the solution dropwise. The mixture was stirred for 48 hours at room temperature. The precipitate was filtered and washed with cold methanol to give the product **3-[[3,5-bis(trifluoromethyl) phenyl]amino]-4-methoxy-3-cyclobutene-1,2-dione** (2.78 g) in 82% yield.

A mixture of **L-NH$_2$-Me** (361 mg, 1.0 mmol), **3-[[3,5-bis(trifluoromethyl) phenyl]amino]-4-methoxy-3-cyclobutene-1,2-dione** (678 mg, 2.0 mmol) and $Zn(OTf)_2$ (72 mg, 0.2 mmol) in 40 mL dry toluene and 4 mL dry DMF was heated at 100 °C for 72 hours. The formed precipitate was isolated by filtration. The resulting solid was then washed with methanol three times to afford **L-SQA-F-Me** (434 mg) in 65% yield. ^{1}H NMR (600 MHz, DMSO-d_6) δ 10.25 (s, 1H), 9.62 (s, 1H), 8.07 (dd, J = 8.3, 2.0 Hz, 4H), 8.00 (s, 2H), 7.95 – 7.92 (m, 2H), 7.80 (s, 1H), 7.77 – 7.74 (m, 2H), 7.71 (s, 2H), 7.53 (d, J = 8.0 Hz, 1H), 3.89 (s, 6H). Due to the low solubility of **L-SQA-F-Me**, its ^{13}C NMR spectrum cannot be obtained. HRMS (ESI): m/z Calculated for $C_{34}H_{22}F_6N_2O_6Na$ [M+ Na$^+$]: 691.1274, found 691.1277.

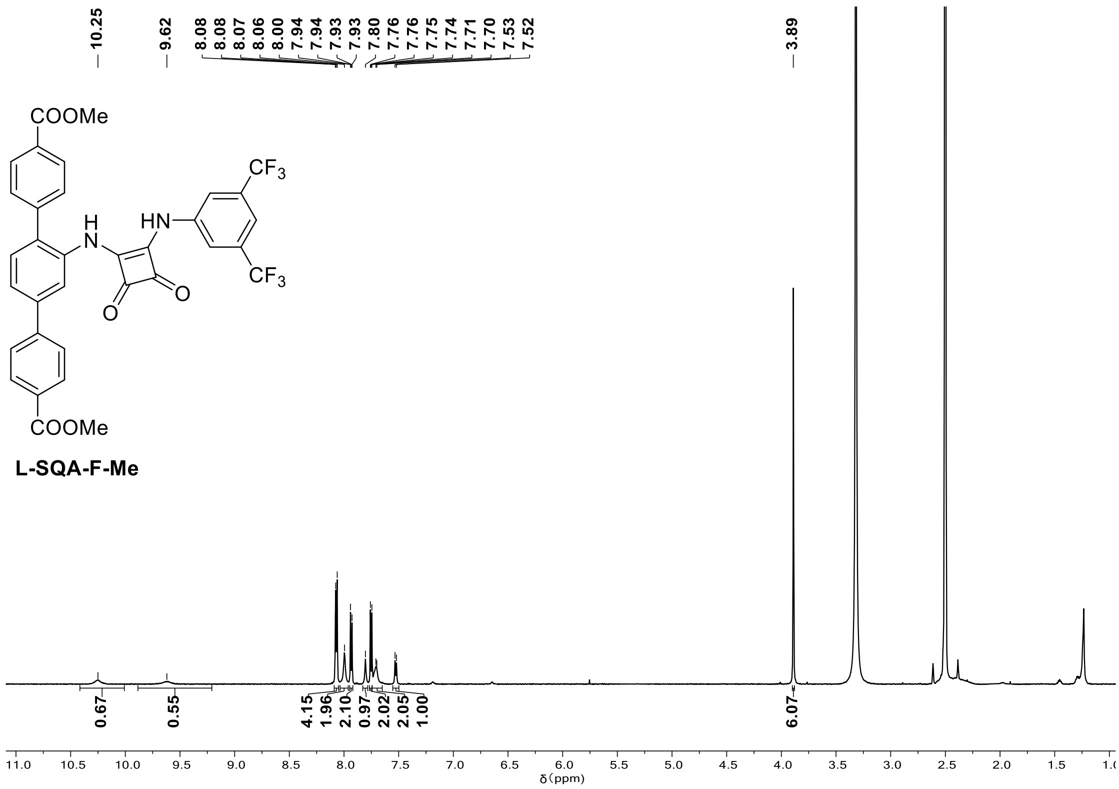

Figure S14. ^{1}H NMR spectrum (600 MHz) of **L-SQA-F-Me** in DMSO-d_6.

A suspension of **L-SQA-F-Me** (334 mg, 0.5 mmol) and HOAc (20 mL) was stirred at 0 °C, to which was slowly added H_2SO_4 (2 mL). The mixture was then heated at 100 °C for 72 hours. After cooling down to 0 °C, H_2O (200 mL) was added to the reaction mixture. The resulting yellow precipitate was filtered and washed by water and dichloromethane to give **L-SQA-F-H** (233 mg) in 73 % yield. ^{1}H NMR (600 MHz, DMSO-d_6) δ 10.24 (s, 1H), 9.54 (s, 1H), 8.03 – 7.99 (m, 4H), 7.95 (s, 2H), 7.89 – 7.84 (m, 2H), 7.75 (m, 1H), 7.67 (m, 4H), 7.48 (m, 1H). Due to the low solubility of **L-SQA-F-H**, its ^{13}C NMR spectrum cannot be obtained. HRMS (ESI): m/z Calculated for $C_{32}H_{18}F_6N_2O_6$ [M^+]: 640.1069, found 691.1055.

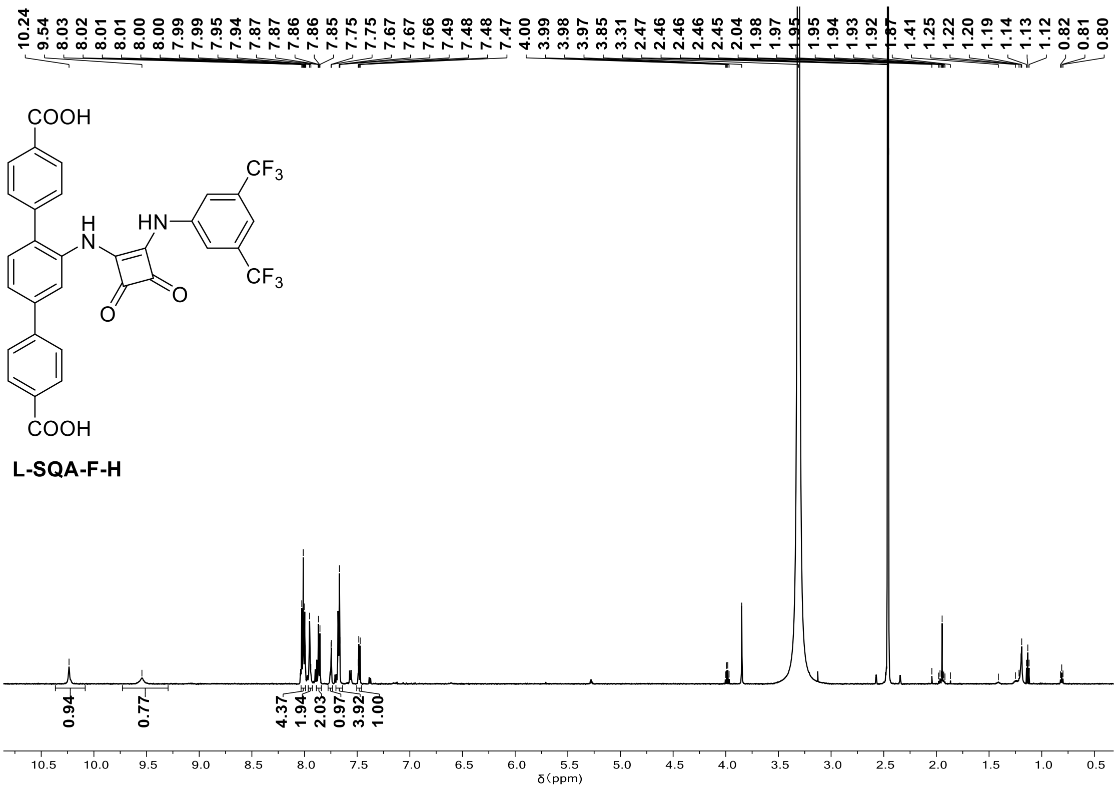

Figure S15. ^{1}H NMR spectrum (600 MHz) of **L-SQA-F-H** in DMSO-d_6.

2.2 Synthesis of MOF-DHBDs

2.2.1 Synthesis of MOF-NH2

$ZrCl_4$ (42 mg) and HCl (16 μl, 37%) were mixed in 2 mL of dimethylformamide (DMF) and sonicated until being fully dissolved. Then, L-PZDB-H (25.3 mg, 0.06 mmol), L-NH_2-H (40 mg, 0.12 mmol) and L-proline (103 mg) were mixed in 2 mL of dimethylformamide (DMF) and sonicated for 10 min. The prepared two solutions were mixed and sonicated for 1 min. Later, the resulting yellow suspension was heated in an oven at 100 ºC for 48 h, followed by cooling to room temperature to afford yellow octahedron crystals, **MOF-NH$_2$(1:2)**. Other **MOF-NH$_2$(1:1)** and **MOF-NH$_2$(2:1)** MOFs were synthesized by following the similar procedure with different ratios of L-PZDB-H and L-NH_2-H (0.18 mmol in total).

The activation of these MOFs followed the procedure below. As synthesized MOFs were first soaked in 10 mL DMF for 1 day (change the solvent with fresh DMF every

8 h). Afterwards, the MOFs were collected by centrifugation, washed by DCM (10 mL*6) and hexane (10 mL*6) and dried under vacuum overnight.

2.2.2 Synthesis of MOF-F-TU

Activated **MOF-NH_2(1:2)** (0.05 mmol based on L-NH_2) was soaked in 5 mL of dry tetrahydrofuran (THF) in a 20 mL vial. Then, 3,5-bis(trifuoromethyl)phenyl isothiocyanate (0.25 mmol, 5 eq) was added to the mixture slowly at room temperature. After being sealed, the vial was heated in an oven at 60 ^oC for 2 days to afford brown crystals **MOF-F-TU(1:2)**. After cooling down to room temperature, the MOFs were washed by DCM (10 mL*6) and hexane (10 mL*6) and dried under vacuum overnight. Other **MOF-F-TU(2:1)** and **MOF-F-TU(1:1)** MOFs were prepared from the corresponding **MOF-NH_2(2:1)** and **MOF-NH_2(1:1)**, respectively, following a similar procedure.

The activation of these MOFs followed the procedure below. As synthesized MOFs were first soaked in 10 mL DMF for 3 days (change the solvent with fresh DMF every 8 h). Afterwards, the MOFs were collected by centrifugation, washed by DCM (10 mL*6) and hexane (10 mL*6) and dried under vacuum overnight.

Note: Attempts to prepare **MOF-F-TU** through a mixed ligand approach failed (using L-PZDB-H and L-TU-H ligands).

2.2.3 Synthesis of MOF-F-U

Activated **MOF-NH_2(1:2)** (0.05 mmol based on L-NH_2) was soaked in 5 mL of dry tetrahydrofuran (THF) in a 20 mL vial. Then, 3,5-bis(trifuoromethyl)phenyl isocyanate (0.25 mmol, 5 eq) was added to the mixture slowly at room temperature. After being sealed, the vial was heated in an oven at 80 ^oC for 2 days to afford brown crystals **MOF-F-U(1:2)**. After cooling down to room temperature, the MOFs were washed by DCM (10 mL*6) and hexane (10 mL*6) and dried under vacuum overnight.

Note: Attempts to prepare **MOF-F-U** through a mixed ligand approach failed (using L-PZDB-H and L-TU-H ligands).

2.2.4.1 W13w333Synthesis of MOF-R-SQA1(1:2)

$ZrCl_4$ (42 mg) and HCl (16 µl, 37%) were mixed in 2 mL of dimethylformamide (DMF) and sonicated until being fully dissolved. Then, L-PZDB-H (25.3 mg, 0.06 mmol), L-SQA-R1-H (42.2 mg, 0.12 mmol) and L-proline (103 mg) were mixed in 2 mL of dimethylformamide (DMF) and sonicated for 10 min. The prepared two solutions were mixed and sonicated for 1 min. Later, the resulting yellow suspension was heated in an oven at 100 ^{o}C for 48 h, followed by cooling to room temperature to afford yellow octahedron crystals **MOF-R-SQA1(1:2)**. Other **MOF-R-SQA1(1:1)** and **MOF-R-SQA1(2:1)** MOFs were synthesized by following the similar procedure with different ratios of L-PZDB-H and L-SQA-R1-H (0.18 mmol in total).

The activation of these MOFs followed the procedure below. As synthesized MOFs were first soaked in 10 mL DMF for 3 days (change the solvent with fresh DMF every 8 h). Afterwards, the MOFs were collected by centrifugation, washed by DCM (10 mL*6) and hexane (10 mL*6) and dried under vacuum overnight.

2.2.5 Synthesis of MOF-R-SQA2(1:2)

$ZrCl_4$ (60 mg) and HCl (16 µl, 37%) were mixed in 2 mL of dimethylformamide (DMF) and sonicated until being fully dissolved. Then, L-PZDB-H (25.3 mg, 0.06 mmol), L-SQA-R2-H (46.5 mg, 0.12 mmol) and L-proline (103 mg) were mixed in 4 mL of dimethylformamide (DMF) and sonicated for 10 min. The prepared two solutions were mixed and sonicated for 1 min. Later, the resulting yellow suspension was heated in an oven at 100 ^{o}C for 48 h, followed by cooling to room temperature to afford yellow octahedron crystals **MOF-R-SQA2(1:2)**.

The activation of these MOFs followed the procedure below. As synthesized MOFs were first soaked in 10 mL DMF for 3 days (change the solvent with fresh DMF every 8 h). Afterwards, the MOFs were collected by centrifugation, washed by DCM (10 mL*6) and hexane (10 mL*6) and dried under vacuum overnight.

2.2.6 Synthesis of MOF-F-SQA(1:2)

Activated **MOF-NH$_2$(1:2)** (0.05 mmol based on L-NH$_2$) was soaked in 5 mL of dry toluene and 500 μl of dry DMF in a 10 mL vial in glovebox. Then, 3-(3,5-bis(trifluoromethyl)phenylamino)-4-methoxycyclobut-3-ene-1,2-dione (0.25 mmol, 5 eq) was added to the mixture, followed by the addition of Zn(OTf)$_2$ (0.01mmol, 20 mol%). After being sealed, the vial was sonicated for 10 min and heated in an oven at 100 ^{o}C for 3 days to afford yellow crystals **MOF-F-SQA(1:2)**. After cooling down to room temperature, the MOFs were washed by DMF (10 mL*6), DCM (10 mL*6), and hexane (10 mL*6) and dried under vacuum overnight.

Note: The conversion of NH$_2$ groups in **MOF-NH$_2$(1:2)** to the target SQA2 was not completely achieved (see Fig. S21 *vide infra*). Attempts to prepare **MOF-F-SQA** MOFs through a mixed ligand approach failed (using L-PZDB-H and L-SQA-F-H ligands).

2.3 Characterization of MOF-DHBDs

2.3.1 Composition determination of MOF-DHBDs

To identify the composition, activated **MOF-DHBD** (2 mg), D_3PO_4 (0.1 mL) or K_3PO_4 (10 mg), D_2O (0.1 mL), and DMSO-d_6 (1 mL) were mixed in a glass vial. After complete sonication, the limpid solution was subjected to ^{1}H NMR analyses, whose results showed the ratios between L-PZDB-H and L-DHBD linkers.

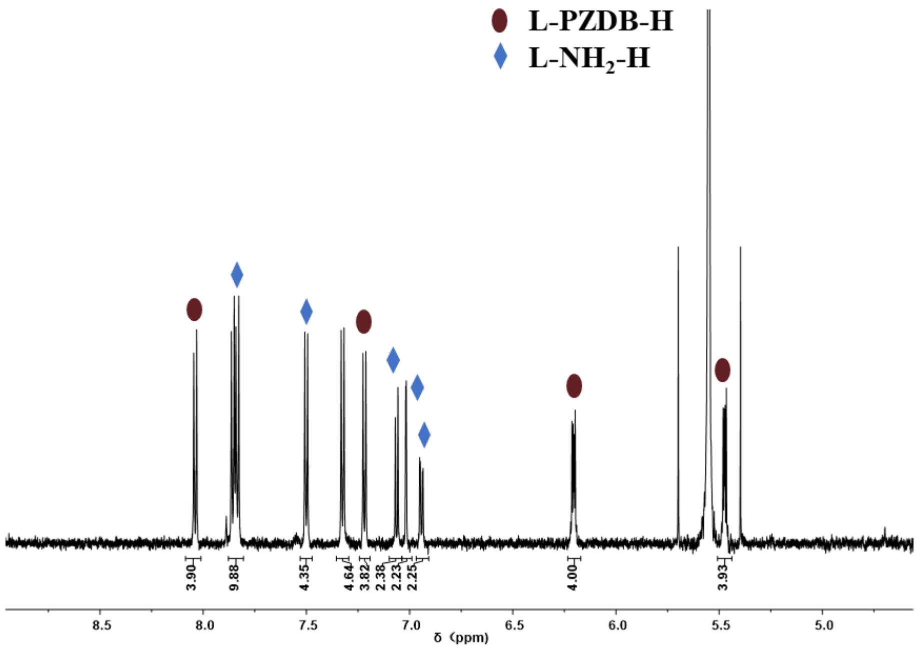

Figure S16. ^{1}H NMR spectrum of **MOF-NH$_2$(1:2)** in K_3PO_4/D_2O/DMSO-*d*$_6$; the integration ratio indicated a formula of $Zr_6(\mu_3\text{-}O)_4(\mu_3\text{-}OH)_4(PZDB)_{1.8}(NH_2)_{4.2}$ for **MOF-NH$_2$(1:2)**.

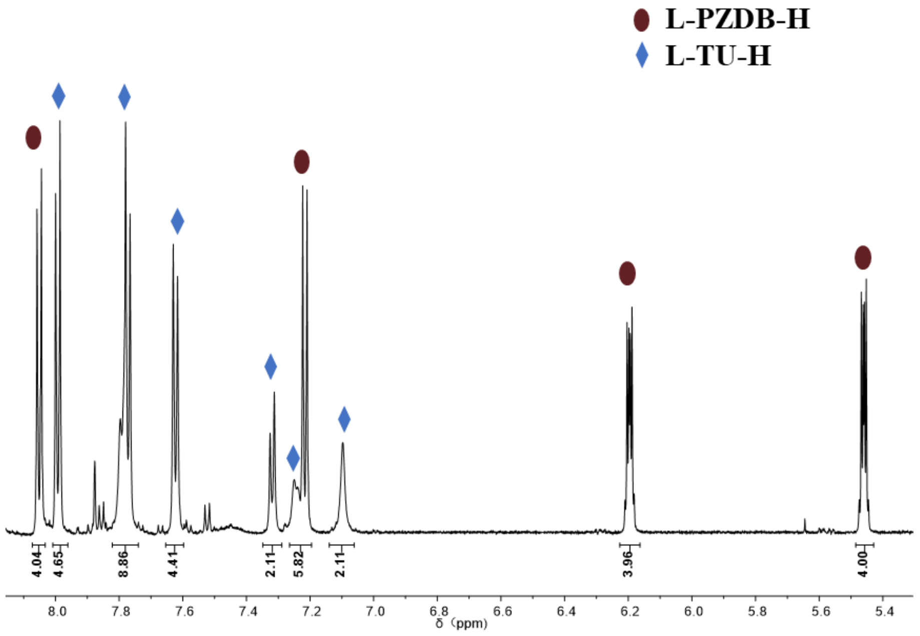

Figure S17. ^{1}H NMR spectrum of **MOF-F-TU(1:2)** in K_3PO_4/D_2O/DMSO-*d*$_6$; the integration ratio indicated a formula of $Zr_6(\mu_3\text{-}O)_4(\mu_3\text{-}OH)_4(PZDB)_{1.8}(TU)_{4.2}$ for **MOF-F-TU(1:2)**.

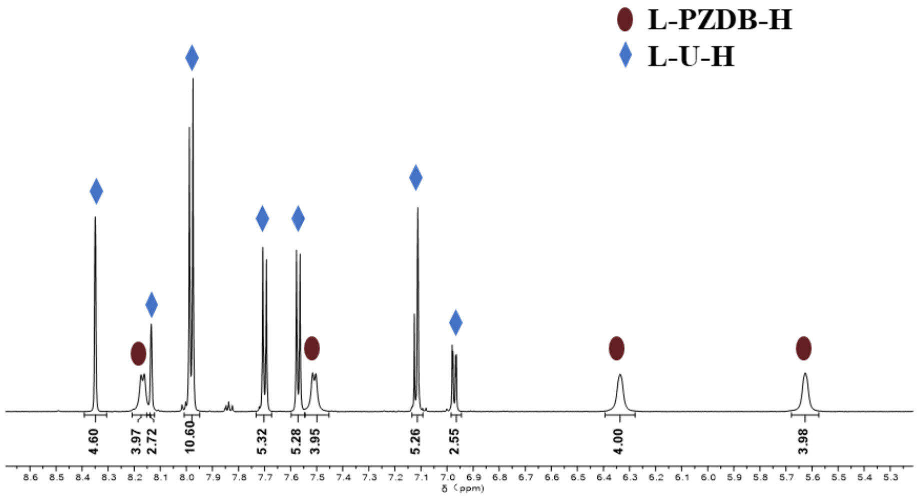

Figure S18. ^{1}H NMR spectrum of **MOF-F-U(1:2)** in D_3PO_4/D_2O/DMSO-*d*$_6$; the integration ratio indicated a formula of $Zr_6(\mu_3\text{-}O)_4(\mu_3\text{-}OH)_4(PZDB)_{1.7}(U)_{4.3}$ for **MOF-F-U(1:2)**.

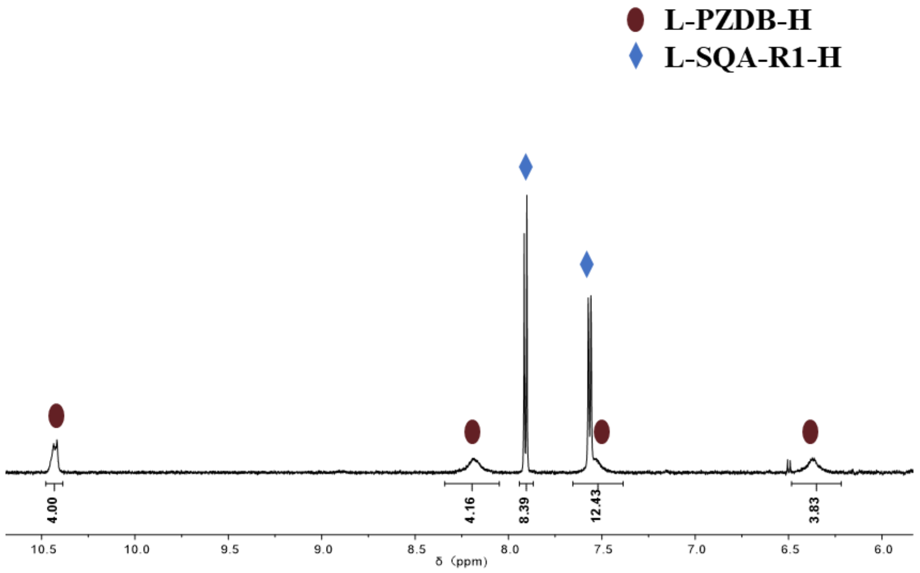

Figure S19. ^{1}H NMR spectrum of **MOF-R-SQA1(1:2)** in D_3PO_4/D_2O/DMSO-*d*$_6$; the integration ratio indicated a formula of $Zr_6(\mu_3\text{-}O)_4(\mu_3\text{-}OH)_4(PZDB)_{1.9}(SQA\text{-}R1)_{4.1}$ for **MOF-R-SQA1(1:2)**.

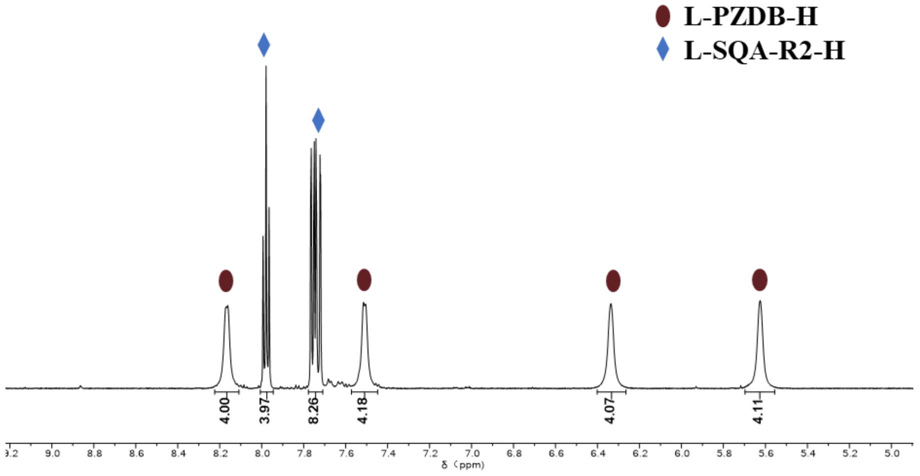

Figure S20. ^{1}H NMR spectrum of **MOF-R-SQA2(1:2)** in D_3PO_4/D_2O/DMSO-d_6; the integration ratio indicated a formula of $Zr_6(\mu_3\text{-}O)_4(\mu_3\text{-}OH)_4(PZDB)_{2.0}(SQA\text{-}R2)_{4.0}$ for **MOF-R-SQA2(1:2)**.

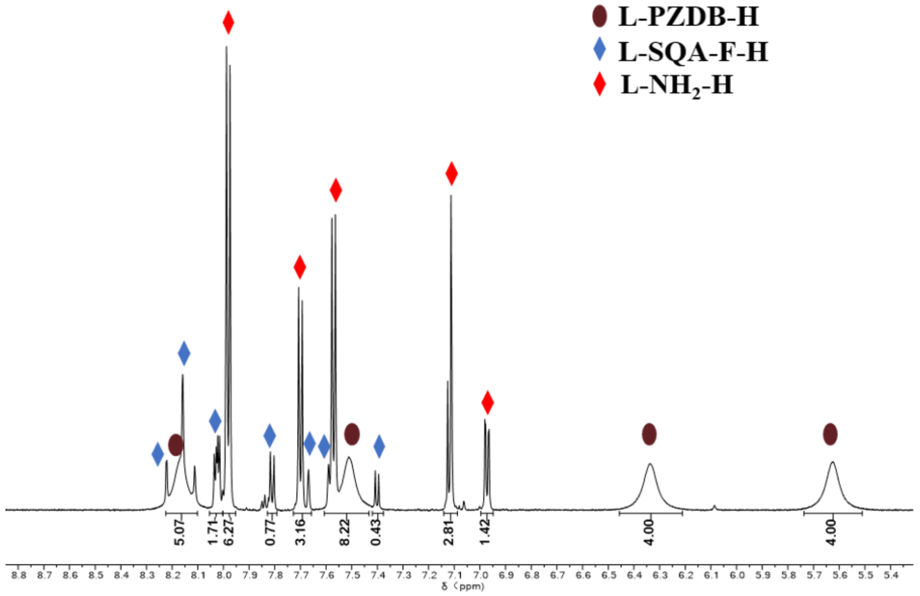

Figure S21. ^{1}H NMR spectrum of **MOF-F-SQA(1:2)** in D_3PO_4/D_2O/DMSO-d_6; the conversion of L-NH_2 to L-SQA is not completely obtained. The integration ratio indicated a formula of $Zr_6(\mu_3\text{-}O)_4(\mu_3\text{-}OH)_4(PZDB)_{2.2}(SQA)_{0.9}(NH_2)_{2.9}$ for **MOF-F-SQA(1:2)**.

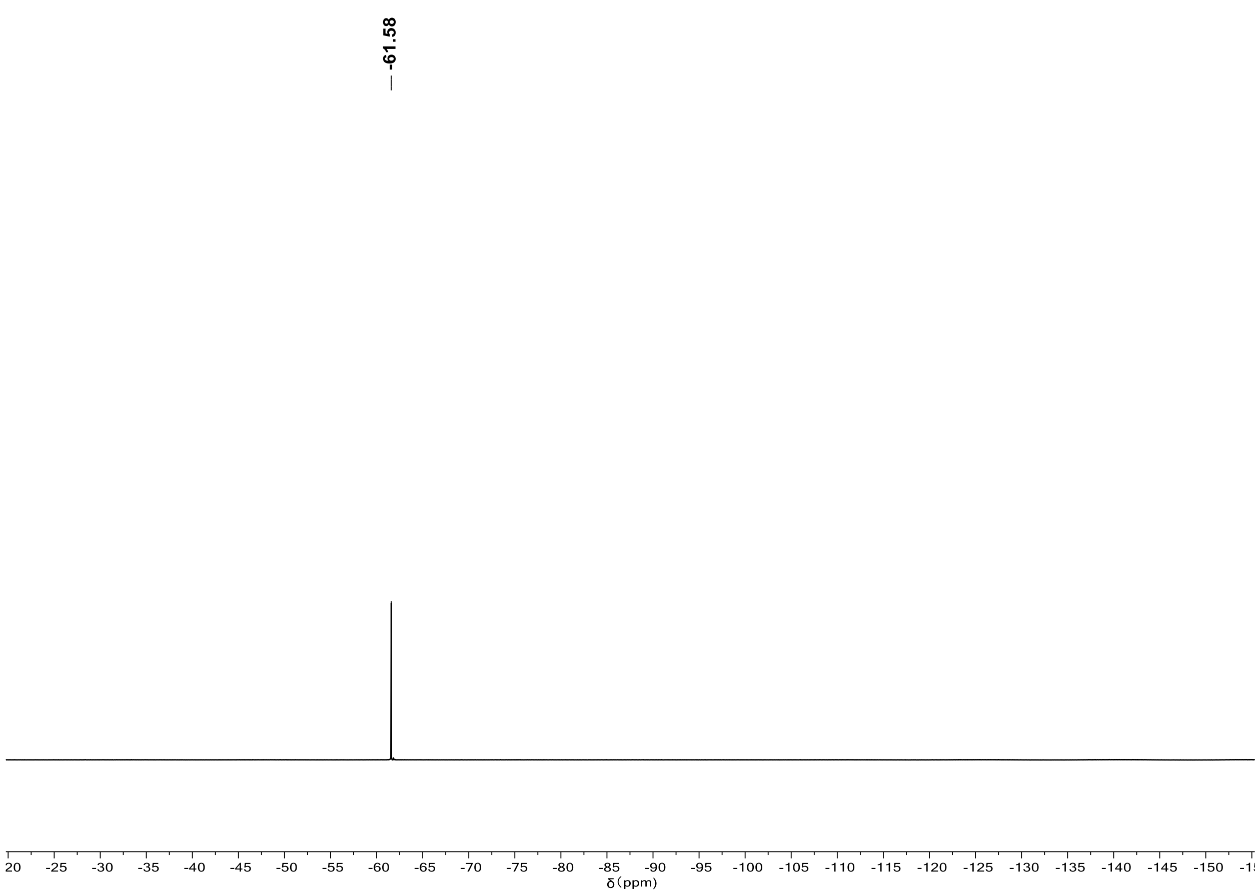

Figure S22. ^{19}F NMR spectrum of **MOF-F-SQA(1:2)** in D_3PO_4/D_2O/DMSO-d_6, matching with the ^{19}F NMR spectrum of L-SQA-F-H.

2.3.2 Thermogravimetric analysis (TGA) of MOF-DHBDs

TGA analyses were conducted to further verify the proposed formulas for **MOF-DHBD** MOFs.

MOF-NH_2(1:2): The first weight loss (6.4%) in the 25 to 250 °C temperature range corresponds to the removal of adsorbed solvents in MOF. The second weight loss (69.7%) in the 250 to 800 °C temperature range corresponds to the decomposition of MOF to metal oxides $(ZrO_2)_6$. The ratio of 74.1% (69.7/94.0) is consistent with a calculated weight loss of 73.9% according to the conversion of $Zr_6(\mu_3\text{-}O)_4(\mu_3\text{-}OH)_4(C_{26}H_{16}N_2O_4)_{1.8}(C_{20}H_{13}NO_4)_{4.2}$ to $(ZrO_2)_6$.

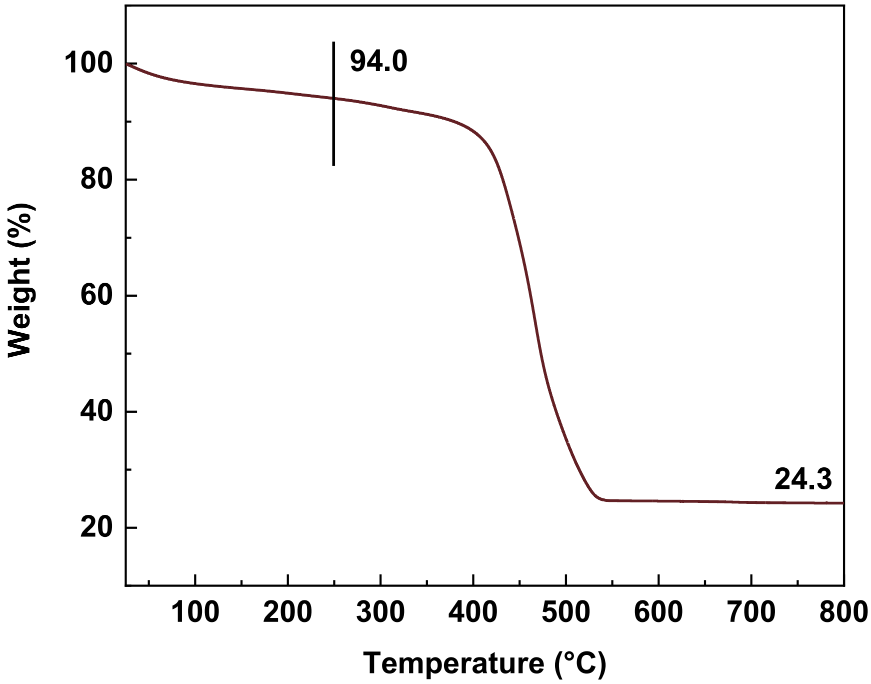

Figure S23. TGA plot of **MOF-NH$_2$** (in air, 15 °C/min).

MOF-F-TU(1:2): The first weight loss (9.9%) in the 25 to 100 °C temperature range corresponds to the removal of adsorbed solvents in MOF. The second weight loss (71.6%) in the 200 to 800 °C temperature range corresponds to the decomposition of MOF to metal oxides $(ZrO_2)_6$. The ratio of 79.4% (71.6/90.1) is consistent with a calculated weight loss of 81.3% according to the conversion of $Zr_6(\mu_3\text{-}O)_4(\mu_3\text{-}OH)_4(C_{26}H_{16}N_2O_4)_{1.8}$ $(C_{29}H_{16}F_6N_2O_4S)_{4.2}$ to $(ZrO_2)_6$.

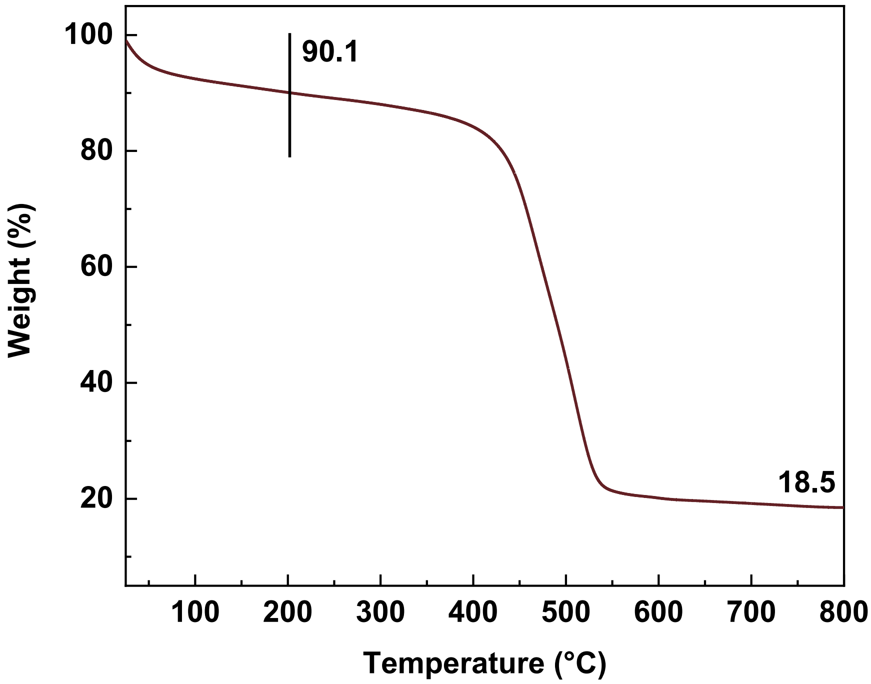

Figure S24. TGA plot of **MOF-F-TU** (in air, 15 °C/min).

MOF-F-U(1:2): The first weight loss (10.1 %) in the 25 to 100 °C temperature range corresponds to the removal of adsorbed solvents in MOF. The second weight loss (71.5%) in the 200 to 800 °C temperature range corresponds to the decomposition of MOF to metal oxides $(ZrO_2)_6$. The ratio of 79.5% (71.5/89.9) is consistent with a calculated weight loss of 81.1% according to the conversion of $Zr_6(\mu_3\text{-}O)_4(\mu_3\text{-}OH)_4(C_{26}H_{16}N_2O_4)_{1.7}(C_{29}H_{16}F_6N_2O_5)_{4.3}$ to $(ZrO_2)_6$.

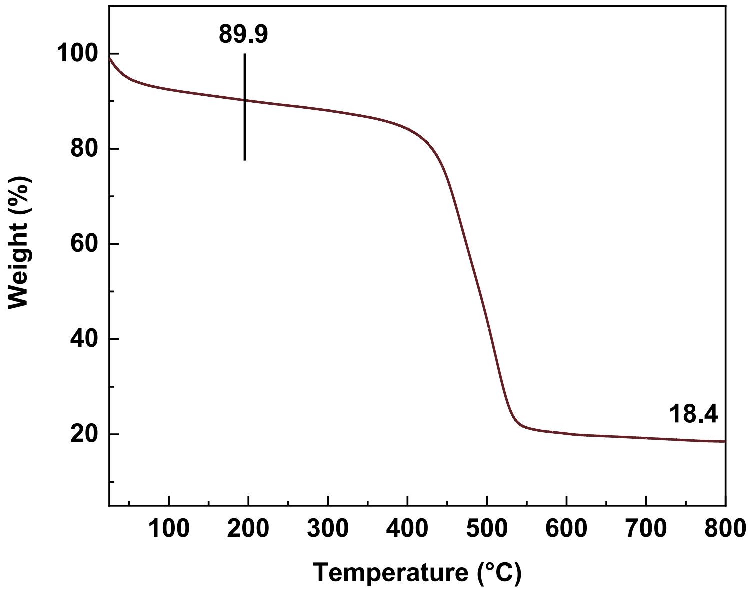

Figure S25. TGA plot of **MOF-F-U** (in air, 15 °C/min).

MOF-R-SQA1(1:2): The first weight loss (16.1%) in the 25 to 300 °C temperature range corresponds to the removal of adsorbed solvents in MOF. The second weight loss (65.9%) in the 300 to 800 °C temperature range corresponds to the decomposition of MOF to metal oxides $(ZrO_2)_6$. The ratio of 78.5% (65.9/83.9) is consistent with a calculated weight loss of 75.6% according to the conversion of $Zr_6(\mu_3\text{-}O)_4(\mu_3\text{-}OH)_4(C_{26}H_{16}N_2O_4)_{1.9}(C_{18}H_{10}N_2O_6)_{4.1}$ to $(ZrO_2)_6$.

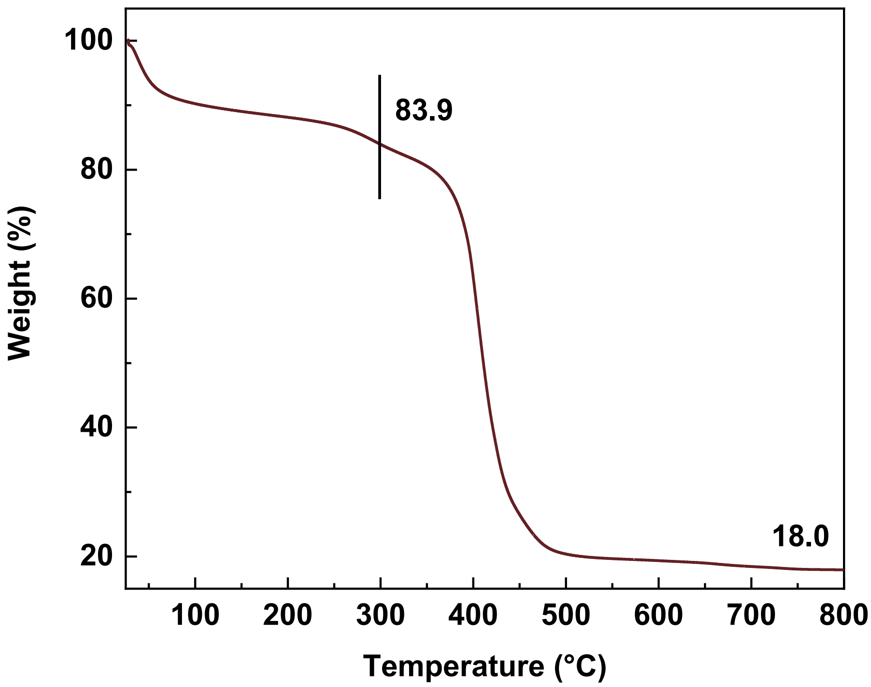

Figure S26. TGA plot of **MOF-R-SQA1** (in air, 15 °C/min).

MOF-R-SQA2(1:2): The first weight loss (7.6%) in the 25 to 200 °C temperature range corresponds to the removal of adsorbed solvents in MOF. The second weight loss (71.0%) in the 200 to 800 °C temperature range corresponds to the decomposition of MOF to metal oxides $(ZrO_2)_6$. The ratio of 76.8% (71.0/92.4) is consistent with a calculated weight loss of 76.9% according to the conversion of $Zr_6(\mu_3\text{-}O)_4(\mu_3\text{-}OH)_4(C_{26}H_{16}N_2O_4)_{1.9}(C_{18}H_8N_2O_6F_2)_{4.1}$ to $(ZrO_2)_6$.

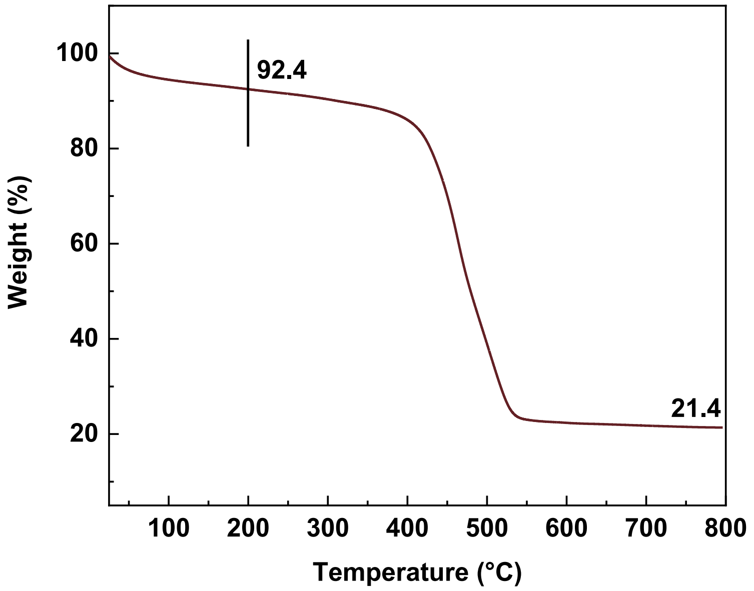

Figure S27. TGA plot of **MOF-R-SQA2** (in air, 15 °C/min).

MOF-F-SQA (1:2): The first weight loss (8.4%) in the 25 to 200 °C temperature range corresponds to the removal of adsorbed solvents in MOF. The second weight loss (72.0%) in the 200 to 800 °C temperature range corresponds to the decomposition of MOF to metal oxides $(ZrO_2)_6$. The ratio of 78.6% (72.0/91.6) is consistent with a calculated weight loss of 76.5% according to the conversion of $Zr_6(\mu_3\text{-}O)_4(\mu_3\text{-}OH)_4(C_{26}H_{16}N_2O_4)_{2.2}(C_{20}H_{13}NO_4)_{2.9}(C_{32}H_{16}F_2N_2O_6)_{0.9}$ to $(ZrO_2)_6$.

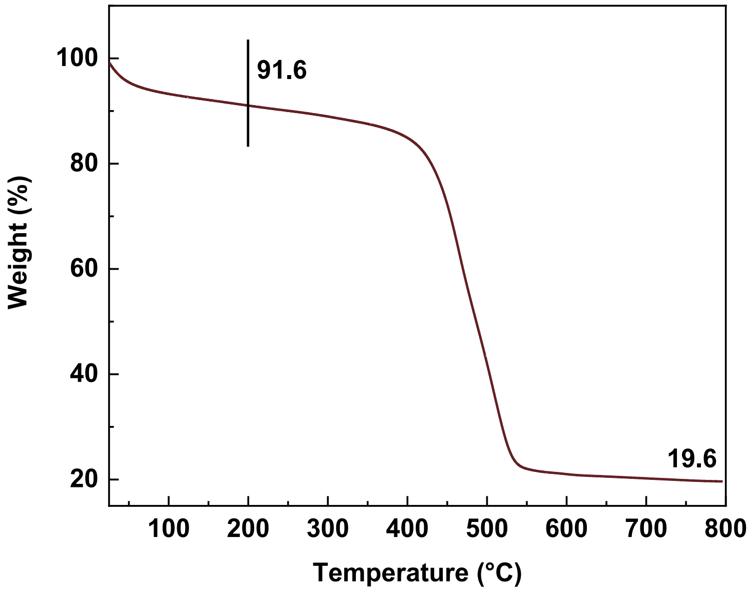

Figure S28. TGA plot of **MOF-F-SQA** (in air, 15 °C/min).

2.3.3 N_2 adsorption analyses of MOF-DHBDs

The activated **MOF-DHBD** (40 mg) was loaded in an adsorption tube and activated at 60 °C for 12 hours before the adsorption measurement.

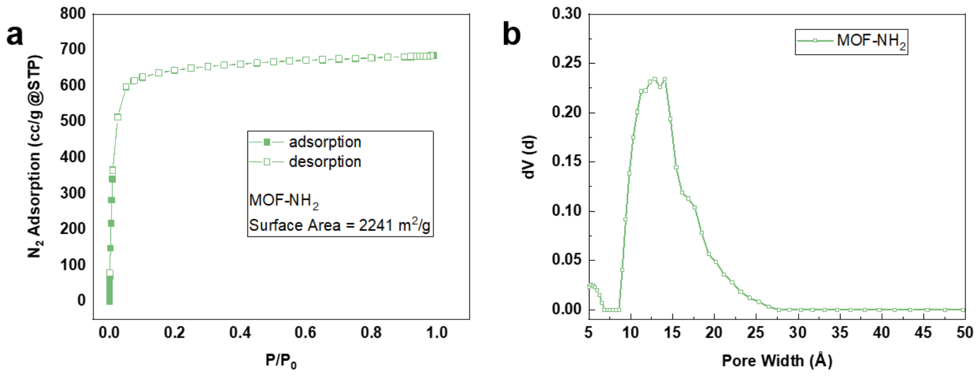

Figure S29. a) Adsorption and desorption isotherm of **MOF-NH2(1:2)**, illustrating type-I adsorption behavior; the BET surface area being calculated as 2241 $m^2 \cdot g^{-1}$. b) Pore-size distribution of **MOF-NH2(1:2)** based on QSDFT analysis, disclosing a major pore size of 14 Å.

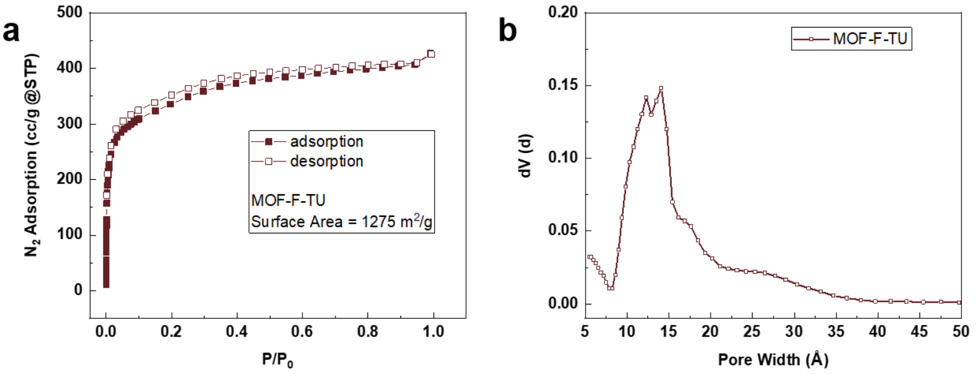

Figure S30. a) Adsorption and desorption isotherm of **MOF-F-TU(1:2)**, illustrating type-I adsorption behavior; the BET surface area being calculated as 1275 $m^2 \cdot g^{-1}$. b) Pore-size distribution of **MOF-F-TU(1:2)** based on QSDFT analysis, disclosing a major pore size of 14 Å.

Note: The reduced BET surface of **MOF-F-TU(1:2)** compared to **MOF-NH$_2$(1:2)** also indicates the successful incorporation of TU motifs in MOF pores or channels.

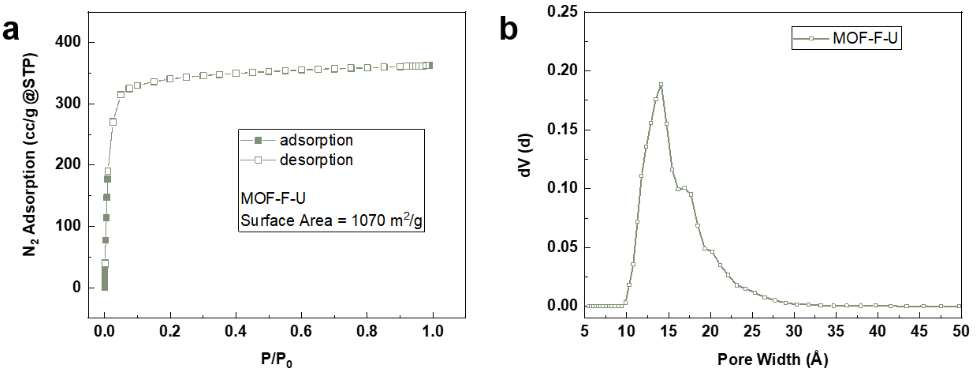

Figure S31. a) Adsorption and desorption isotherm of **MOF-F-U(1:2)**, illustrating type-I adsorption behavior; the BET surface area being calculated as 1070 $m^2 \cdot g^{-1}$. b) Pore-size distribution of **MOF-F-U(1:2)** based on QSDFT analysis, disclosing a major pore size of 14 Å.

Note: The reduced BET surface of **MOF-F-U(1:2)** compared to **MOF-NH$_2$(1:2)** also indicates the successful incorporation of U motifs in MOF pores or channels.

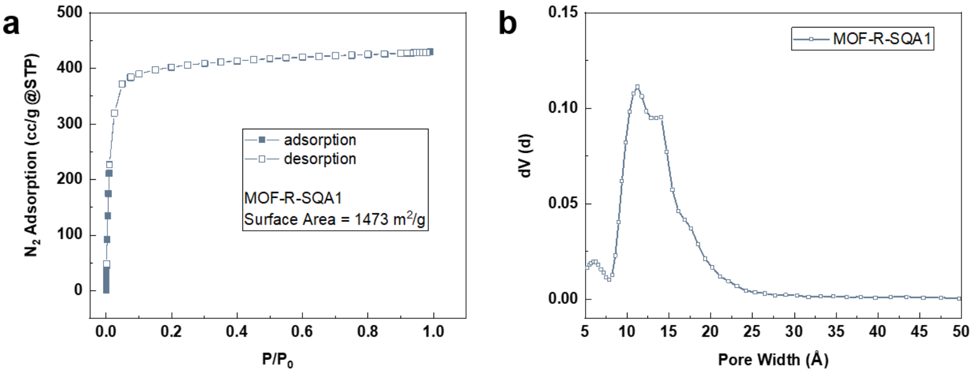

Figure S32. a) Adsorption and desorption isotherm of **MOF-R-SQA1(1:2)**, illustrating type-I adsorption behavior; the BET surface area being calculated as 1473 $m^2 \cdot g^{-1}$. b) Pore-size distribution of **MOF-R-SQA1(1:2)** based on QSDFT analysis, disclosing a major pore size of 12 Å.

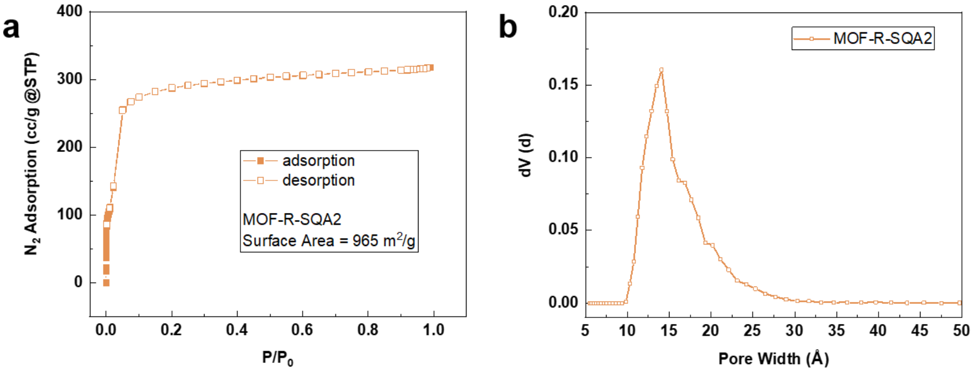

Figure S33. a) Adsorption and desorption isotherm of **MOF-R-SQA2(1:2)**, illustrating type-I adsorption behavior; the BET surface area being calculated as 965 $m^2 \cdot g^{-1}$. b) Pore-size distribution of **MOF-R-SQA2(1:2)** based on QSDFT analysis, disclosing a major pore size of 14 Å.

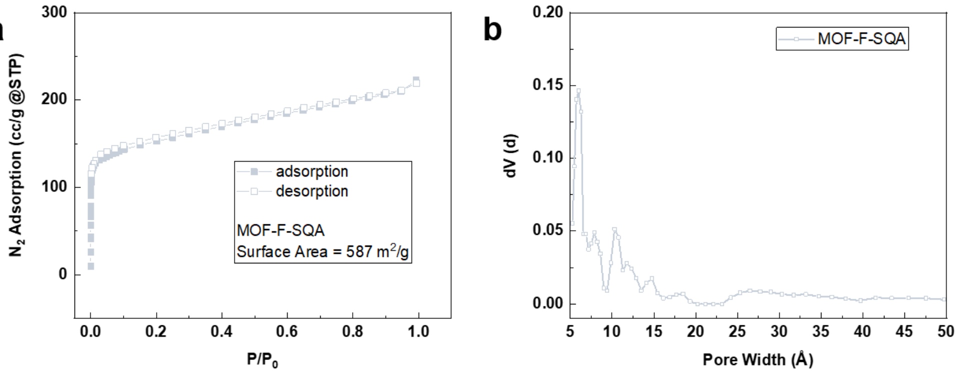

Figure S34. a) Adsorption and desorption isotherm of **MOF-F-SQA(1:2)**, illustrating type-I adsorption behavior; the BET surface area being calculated as 587 $m^2 \cdot g^{-1}$. b) Pore-size distribution of **MOF-F-SQA(1:2)** based on QSDFT analysis, disclosing a major pore size of 6 Å.

Note: The reduced BET surface and pore windows of **MOF-F-SQA(1:2)** compared to **MOF-NH_2(1:2)** may be due to some structural damage of MOFs caused by the relatively harsh conditions of the PSM procedure.

2.3.4 SEM imaging of MOF-DHBDs

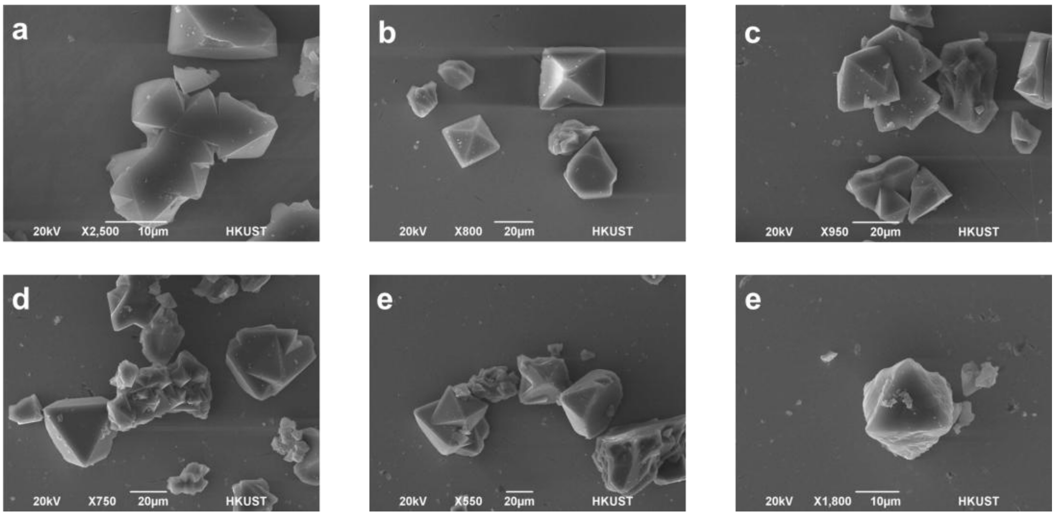

Figure S35. SEM imaging of **MOF-NH_2** a), **MOF-F-TU** b), **MOF-F-U** c), **MOF-R-SQA1** d), **MOF-R-SQA2** e), **MOF-F-SQA** f).

2.3.5 UV-Vis and IR spectra of MOF-DHBDs

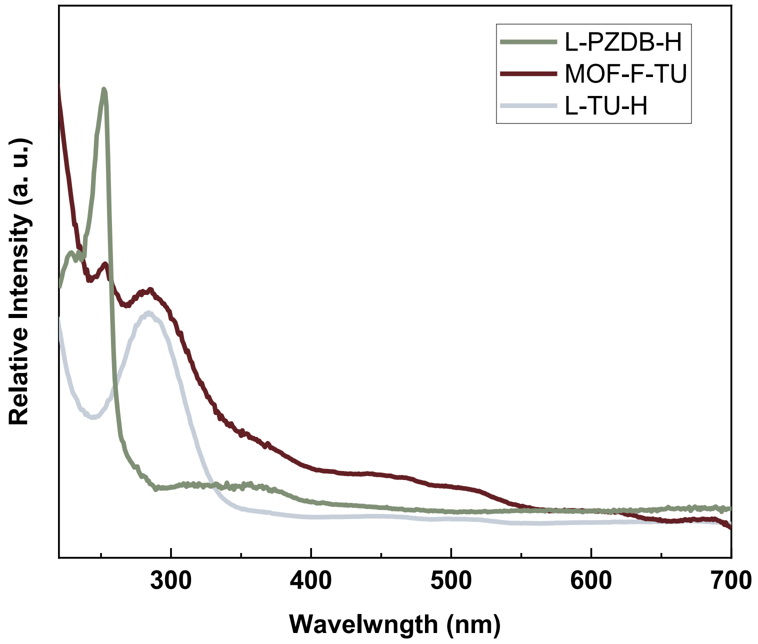

Figure S36. UV-Vis spectra of L-PZDB-H, **MOF-F-TU**, and L-TU-H. **MOF-F-TU** displayed a spectrum containing the features from both L-PZDB-H and L-TU-H.

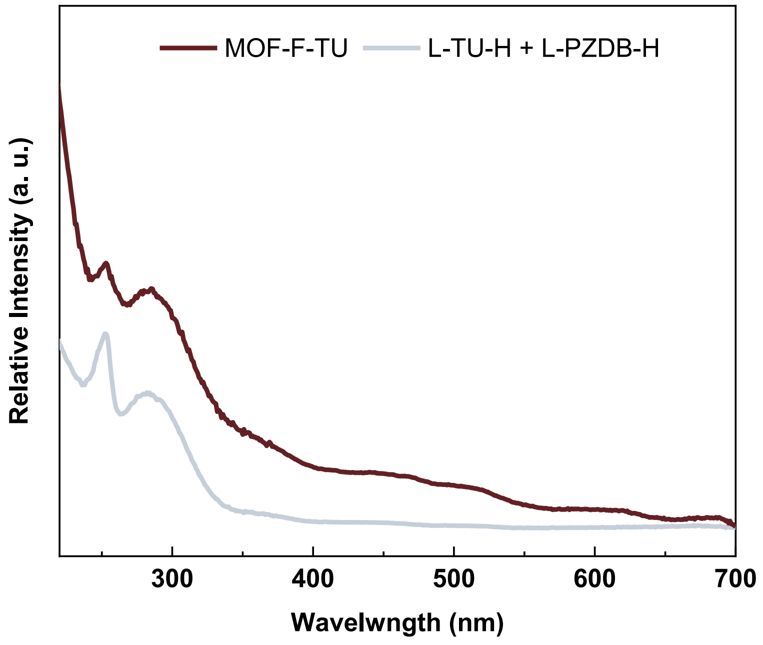

Figure S37. UV-Vis spectra of the mixture of L-PZDB-H and L-TU-H(1:2) and **MOF-F-TU**.

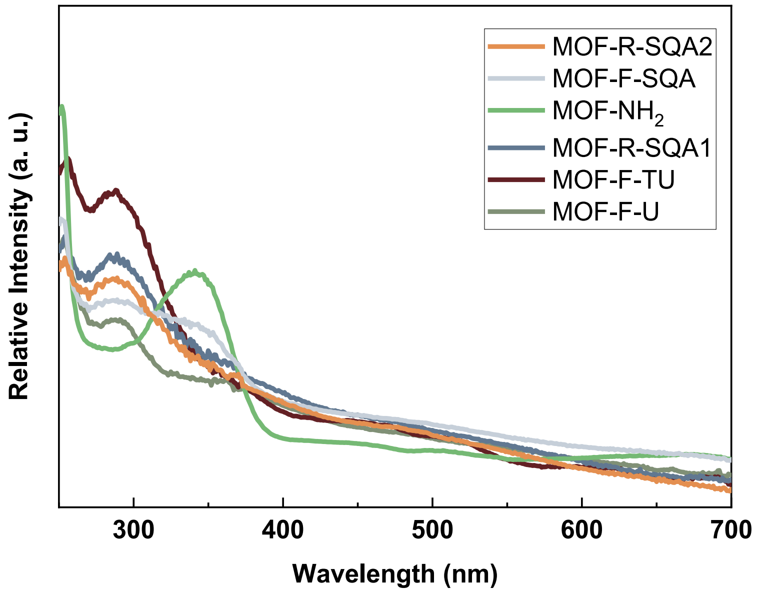

Figure S38. UV-Vis spectra of **MOF-NH₂**, **MOF-F-TU**, **MOF-F-U**, **MOF-R-SQA1**, **MOF-R-SQA2** and **MOF-F-SQA**.

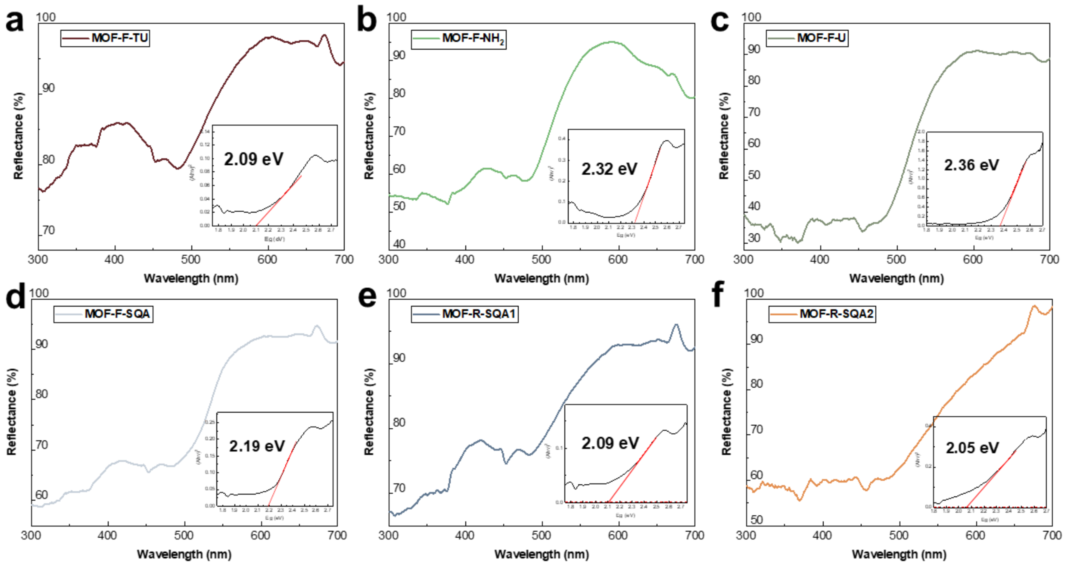

Figure S39. Solid-state UV-Vis diffuse reflectance spectrum of MOFs; inset: the corresponding Tauc plot.

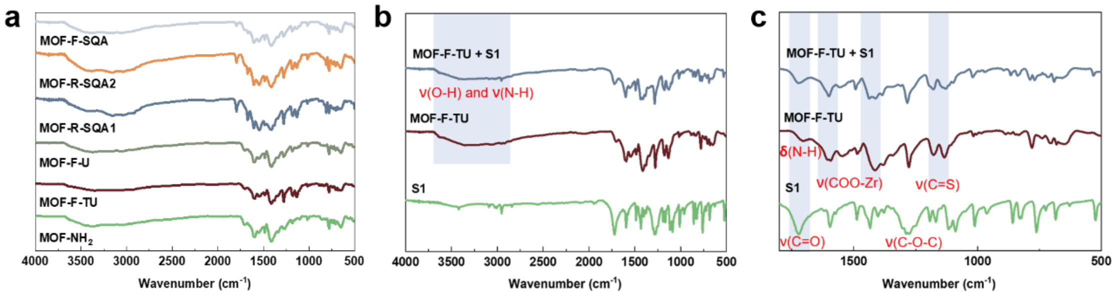

Figure S40. a), IR spectra of **MOF-NH₂**, **MOF-F-TU**, **MOF-F-U**, **MOF-R-SQA1**, **MOF-R-SQA2**, and **MOF-F-SQA**. b), IR spectra of **S1**, **MOF-F-TU** and **MOF-F-TU** after adsorption of **S1**. c), Magnified view of b).

3. Catalytic Reactions

3.1 General procedure

3.1.1 General procedure for dehalogenation

In a N_2-filled glovebox, aryl halide (0.2 mmol), *N*, *N*-diisopropylethylamine (DIPEA, 0.8 mmol), **MOF-DHBD** (0.5 mol% based on the PZDB site), and MeCN (2 mL) were loaded into a 10-mL reaction tube equipped with a magnetic stirring bar. The tube was then taken out from the glovebox, to which was added 0.2 mL degassed H_2O. After being tightly screw-caped, and the reaction mixture was subsequently stirred under irradiation (Kessil PR160L-427) at room temperature for 16 hours. GC-MS analysis of the crude reaction mixture was performed to identify the yield of the product (**1a-1h**).

3.1.2 General procedure for dehalogenative arylation

In a N_2-filled glovebox, aryl halide (0.1 mmol), *N*-methyl pyrrole, thiophene or furan (4 mmol), **MOF-DHBD** (2 mol% based on the PZDB site), and DMSO (1 mL) were loaded into a 4-mL reaction tube equipped with a magnetic stirring bar. After being tightly screw-caped, the tube was taken out from the glovebox and subsequently stirred under irradiation (Kessil PR160L-427) at room temperature for 24 hours. After reaction, purification by column chromatography on silica gel was performed to give pure products **2a-2h** with isolated yields.

3.1.3 General procedure for dehalogenative phosphination

In a N_2-filled glovebox, aryl halide (0.1 mmol), triethyl phosphite (0.5 mmol), **MOF-DHBD** (2 mol% based on the PZDB site), and MeCN (1 mL) were loaded into a 4-mL reaction tube equipped with a magnetic stirring bar. After being tightly screw-caped, the tube was taken out from the glovebox and subsequently stirred under irradiation (Kessil PR160L-427) at room temperature for 24 hours. After reaction, purification by column chromatography on silica gel was performed to give pure products **2i-2k** with isolated yields.

3.1.4 General procedure for remote C-H functionalization through 1,5-HAT

In a N_2-filled glovebox, aryl halide (0.1 mmol), DIPEA (0.2 mmol), Micheal acceptor (0.2 mmol), **MOF-DHBD** (2 mol% based on the PZDB site), and MeCN (1 mL) were loaded into a 4-mL reaction tube equipped with a magnetic stirring bar. After being tightly screw-caped, the tube was taken out from the glovebox, and subsequently stirred under irradiation (Kessil PR160L-427) at room temperature for 24 hours. After reaction, purification by column chromatography on silica gel was performed to give pure products **3a-3e** with isolated yields.

3.1.5 General procedure for decarboxylative alkylation

In a N_2-filled glovebox, redox-active ester (0.1 mmol), Micheal acceptor (0.2 mmol), γ-terpinene (0.3 mmol), **MOF-DHBD** (2 mol% based on the PZDB site), and MeCN (1 mL) were loaded into a 4-mL reaction tube equipped with a magnetic stirring bar. After being tightly screw-caped, the tube was then taken out from the glovebox and subsequently stirred under irradiation (Kessil PR160L-427) at room temperature for 24 hours. After reaction, purification by column chromatography on silica gel was performed to give pure products **4a-4e** with isolated yields.

3.2 Set up of gram scale experiments

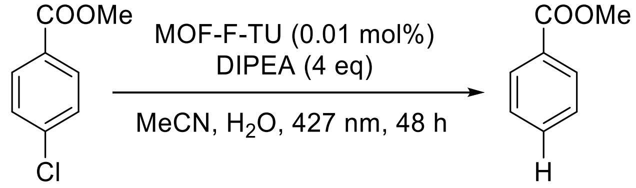

,

Methyl 4-chlorobenzoate (1.70 g, 10 mmol), DIPEA (5.17 g,40 mmol), **MOF-F-TU** (1 mg, 0.01 mol% based on the PZDB site), and MeCN (100 mL) were loaded into a 250-mL reaction tube equipped with a magnetic stirring bar. The tube was then taken out from the glovebox, to which was added 10 mL degassed H_2O. The resulting mixture was stirred and bubbled with nitrogen at room temperature for 30 minutes. Afterwards, the reaction mixture was stirred under irradiation (Kessil PR160L-427) at room temperature for 48 hours. After reaction, the MOF catalyst was removed via

centrifugation. To the resulting solution was added 50 mL aqueous solution of HCl (1M). The organic portioins were extracted with 50 mL DCM for 3 times, combined, and dried by vacuum to give **1a** (1.29 g, 95%) with a turnover number of 9500. Notably, no column chromatography was needed.

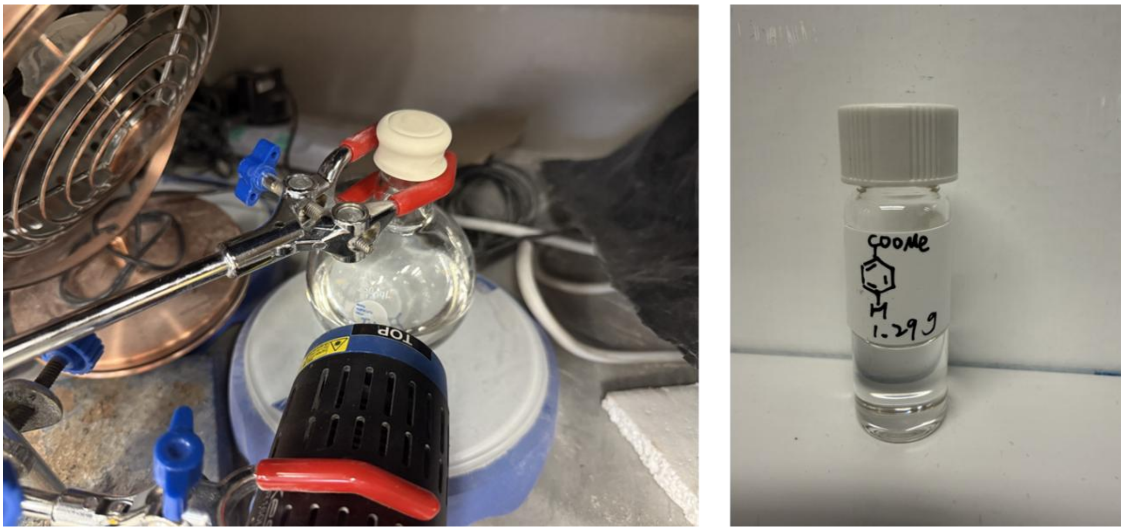

Figure S41. Set up of gram scale experiments.

3.3 Comparison of MOF-F-TU catalysis with homogeneous catalytic systems

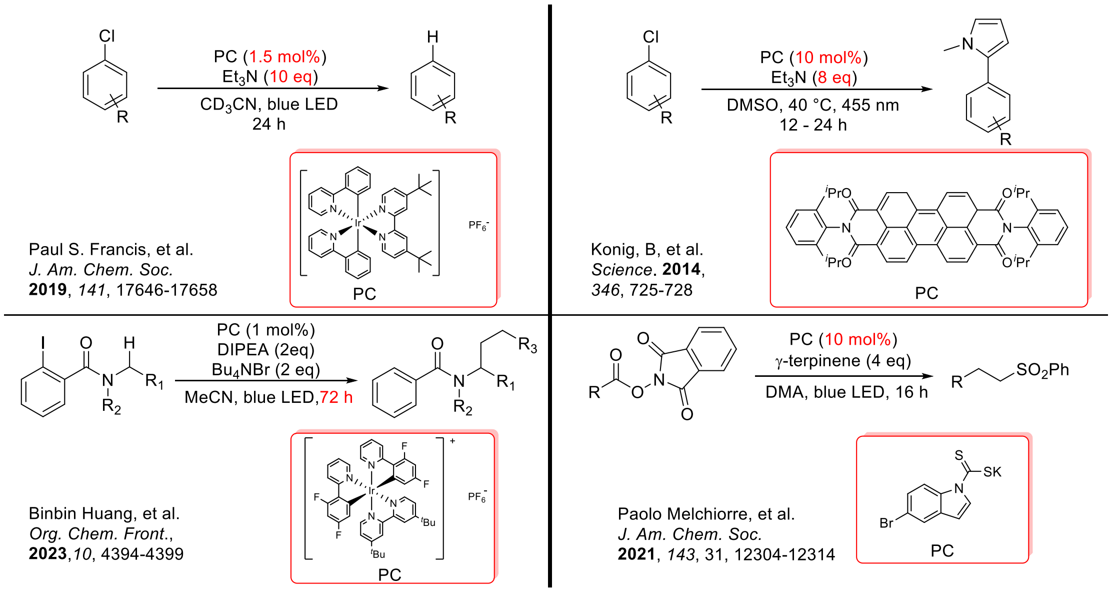

Figure S42. Comparing **MOF-F-TU** catalysis with representative homogeneous catalytic systems.

3.4 NMR data

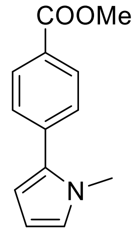

2a: ^{1}H NMR (600 MHz, Chloroform-*d*) δ 8.09 – 8.02 (m, 2H), 7.50 – 7.45 (m, 2H), 6.76 (t, *J* = 2.2 Hz, 1H), 6.33 (m, 1H), 6.22 (m, 1H), 3.93 (s, 3H), 3.71 (s, 3H). ^{13}C NMR (151 MHz, Chloroform-*d*) δ 167.06, 137.85, 133.61, 129.84, 128.00, 125.20, 127.97, 110.14, 108.38, 52.17, 35.47. HRMS (ESI): m/z Calculated for $C_{13}H_{13}NNaO^{+}$ [M+Na^{+}]: 238.0838, found 238.0850.

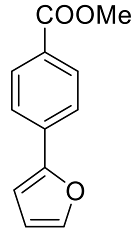

2b: ^{1}H NMR (600 MHz, Chloroform-*d*) δ 8.07 – 8.03 (m, 2H), 7.74 – 7.71 (m, 2H), 7.52 (d, *J* = 1.7 Hz, 1H), 6.78 (d, *J* = 3.4 Hz, 1H), 6.51 (m, 1H), 3.92 (s, 3H). ^{13}C NMR (151 MHz, Chloroform-*d*) δ 166.91, 153.04, 143.22, 134.87, 130.20, 128.65, 123.50, 112.11, 107.31, 52.17. HRMS (ESI): m/z Calculated for $C_{12}H_{11}O_{3}^{+}$ [M+H^{+}]: 203.0703, found 203.0710.

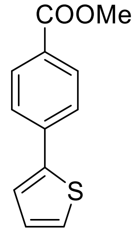

2c: ^{1}H NMR (600 MHz, Chloroform-*d*) δ 8.06 – 8.02 (m, 2H), 7.69 – 7.66 (m, 2H), 7.42 (m, 1H), 7.36 (m, 1H), 7.11 (m, 1H), 3.93 (s, 3H). ^{13}C NMR (151 MHz, Chloroform-*d*) δ 130.37, 128.40, 126.37, 125.62, 124.58, 52.21. HRMS (ESI): m/z Calculated for $C_{12}H_{11}O_{2}S^{+}$ [M+H^{+}]: 219.0474, found 219.0479.

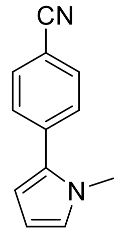

2d: ^{1}H NMR (600 MHz, Chloroform-*d*) δ 7.69 – 7.63 (m, 2H), 7.53 – 7.47 (m, 2H), 6.78 (t, *J* = 2.2 Hz, 1H), 6.35 (m, 1H), 6.23 (m, 1H), 3.71 (s, 3H). ^{13}C NMR (151 MHz, Chloroform-*d*) δ 137.81, 132.72, 132.35, 128.38, 125.94, 119.12, 110.85, 109.81, 108.69, 35.53. HRMS (ESI): m/z Calculated for $C_{12}H_{11}N_{2}^{+}$ [M+H^{+}]: 183.0917, found 183.0921.

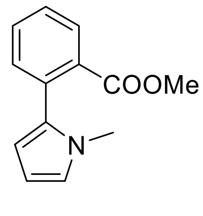

2e: ^{1}H NMR (600 MHz, Chloroform-*d*) δ 7.90 (m, 1H), 7.52 (m, 1H), 7.42 (m, 1H), 7.39 (m, 1H), 6.70 (m, 1H), 6.19 (m, 1H), 6.06 (m, 1H), 3.72 (s, 3H), 3.40 (s, 3H). ^{13}C NMR (151 MHz, Chloroform-*d*) δ 168.18, 133.98, 132.74, 132.47, 132.04, 131.41, 129.88, 127.83, 122.34, 108.52, 107.57, 52.30, 34.25. HRMS (ESI): m/z Calculated for $C_{13}H_{13}NNaO^{+}$ [M+Na^{+}]: 238.0838, found 238.0853.

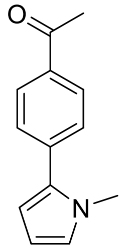

2f: ^{1}H NMR (600 MHz, Chloroform-*d*) δ 8.01 – 7.96 (m, 2H), 7.53 – 7.47 (m, 2H), 6.80 – 6.74 (m, 1H), 6.35 (m, 1H), 6.23 (m, 1H), 3.72 (s, 3H), 2.62 (s, 3H). ^{13}C NMR (151 MHz, Chloroform-*d*) δ 192.89, 133.26, 130.29, 128.75, 123.92, 123.30, 120.63, 105.56, 103.70, 30.75, 21.88. HRMS (ESI): m/z Calculated for $C_{13}H_{14}NO^+$ [M+H$^+$]: 200.1070, found 200.1087.

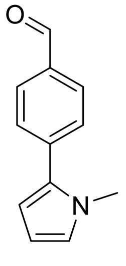

2g: ^{1}H NMR (600 MHz, Chloroform-*d*) δ 10.02 (s, 1H), 7.90 (d, *J* = 8.1 Hz, 2H), 7.57 (d, *J* = 8.1 Hz, 2H), 6.79 (t, *J* = 2.2 Hz, 1H), 6.39 (m, 1H), 6.24 (t, *J* = 3.2 Hz, 1H), 3.74 (s, 3H). ^{13}C NMR (151 MHz, Chloroform-*d*) δ 191.85, 139.35, 134.32, 133.34, 130.08, 128.31, 125.87, 110.83, 108.63, 35.65. HRMS (ESI): m/z Calculated for $C_{12}H_{12}NO^+$ [M+H$^+$]: 186.0913, found 186.0918.

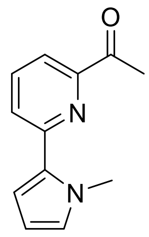

2h: ^{1}H NMR (600 MHz, Chloroform-*d*) δ 7.84 – 7.70 (m, 3H), 6.79 (s, 1H), 6.68 (d, *J* = 4.0 Hz, 1H), 6.21 (s, 1H), 4.11 (s, 3H), 2.73 (s, 3H). ^{13}C NMR (151 MHz, Chloroform-*d*) δ 200.51, 152.39, 152.02, 137.24, 131.26, 127.32, 124.51, 118.04, 111.63, 107.98, 37.75, 26.27. HRMS (ESI): m/z Calculated for $C_{12}H_{13}N_2O^+$ [M+H$^+$]: 201.1022, found 201.1031.

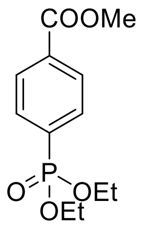

2i: ^{1}H NMR (600 MHz, Chloroform-*d*) δ 8.17 – 8.08 (m, 2H), 7.95 – 7.85 (m, 2H), 4.23 – 4.09 (m, 4H), 3.95 (s, 3H), 1.34 (t, *J* = 7.1 Hz, 6H). ^{13}C NMR (151 MHz, Chloroform-*d*) δ 166.39, 113.72 (d, *J* = 3.0 Hz), 132.94 (d, *J* = 187.2 Hz), 131.91 (d, *J* = 9.1 Hz), 129.53 (d, *J* = 15.1 Hz), 62.81 (d, *J* = 6.0 Hz), 52.58, 16.38 (d, *J* = 6.0 Hz). ^{31}P NMR (243 MHz, Chloroform-*d*) δ 17.71. HRMS (ESI): m/z Calculated for $C_{12}H_{17}NaO_5P^+$ [M+Na$^+$]: 295.0706, found 295.0716.

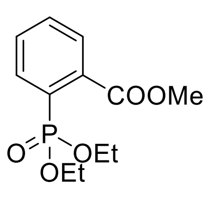

2j: ^{1}H NMR (600 MHz, Chloroform-*d*) δ 7.98 (m, 1H), 7.72 (m, 1H), 7.59 (m, 2H), 4.27 – 4.07 (m, 4H), 3.94 (s, 3H), 1.35 (t, *J* = 7.1 Hz, 6H). ^{13}C NMR (151 MHz, Chloroform-*d*) δ ^{13}C NMR (151 MHz, Chloroform-*d*) δ 168.51 (d, *J* = 5.0 Hz), 136.27 (d, *J* = 8.8 Hz), 133.92 (d, *J* = 8.2 Hz), 132.16 (d, *J* = 2.9 Hz), 130.65 (d, *J* = 14.1 Hz), 129.29 (d,

J = 12.4 Hz), 127.55 (d, J = 187.2 Hz), 62.59 (d, J = 5.7 Hz), 52.73, 16.35 (d, J = 6.6 Hz). ^{31}P NMR (243 MHz, Chloroform-*d*) δ 16.73. HRMS (ESI): m/z Calculated for $C_{12}H_{17}NaO_5P^+$ [M+Na$^+$]: 295.0706, found 295.0712.

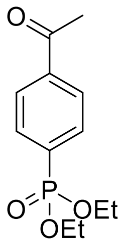

2k: ^{1}H NMR (600 MHz, Chloroform-*d*) δ 8.04 – 8.01 (m, 2H), 7.97 – 7.89 (m, 2H), 4.23 – 4.08 (m, 4H), 2.65 (s, 3H), 1.34 (t, J = 7.1 Hz, 6H). ^{13}C NMR (151 MHz, Chloroform-*d*) δ 197.63, 139.95 (d, J = 3.0 Hz), 132.18 (d, J = 10.0 Hz), 128.16 (d, J = 15.1 Hz), 133.30 (d, J = 187.2Hz), 62.64 (d, J = 5.6 Hz), 26.91, 16.41 (d, J = 6.5 Hz). ^{31}P NMR (243 MHz, Chloroform-*d*) δ 17.71. HRMS (ESI): m/z Calculated for $C_{12}H_{17}NaO_4P^+$ [M+Na$^+$]: 279.0757, found 279.0762.

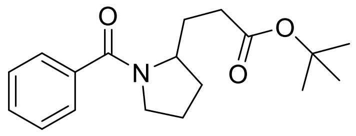

3a: ^{1}H NMR (400 MHz, Chloroform-*d*) δ 7.54 – 7.49 (m, 2H), 7.39 (m, 3H), 4.33 (m 1H), 3.44 (m, 2H), 2.35 (m, 2H), 2.27 – 2.17 (m, 1H), 2.12 – 1.92 (m, 3H), 1.73 (m, 2H), 1.45 (s, 9H). ^{13}C NMR (101 MHz, Chloroform-*d*) δ 172.88, 170.37, 137.16, 129.98, 128.23, 127.37, 80.36, 56.71, 50.12, 32.46, 30.19, 29.28, 28.10, 25.00. HRMS (ESI): m/z Calculated for $C_{18}H_{25}NNaO_3^+$ [M+Na$^+$]: 326.1727, found 326.1730.

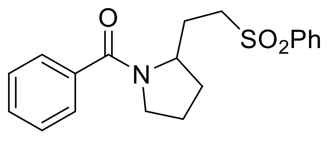

3b: ^{1}H NMR (600 MHz, Chloroform-*d*) δ 7.92 (d, J = 7.8 Hz, 2H), 7.64 (m, 1H), 7.56 (m, 2H), 7.49 – 7.44 (m, 2H), 7.40 (m, 3H), 4.39 (m, 1H), 3.49 (m, 1H), 3.42 (m, 1H), 3.28 (m, 2H), 2.26 (m, 1H), 2.19 – 2.10 (m, 1H), 2.01 (m, 1H), 1.93 (m, 1H), 1.76 (m, 1H), 1.68 (m, 1H). ^{13}C NMR (151 MHz, Chloroform-*d*) δ 170.98, 139.14, 136.67, 133.85, 130.36, 129.44, 128.40, 128.14, 127.44, 55.71, 53.95, 50.23, 30.82, 27.80, 25.01. HRMS (ESI): m/z Calculated for $C_{19}H_{21}NNaO_3S^+$ [M+Na$^+$]: 366.1134, found 366.1141.

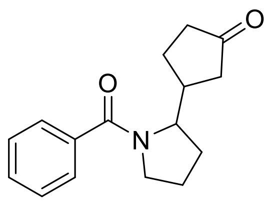

3c: dr = 1.4:1, ^{1}H NMR (600 MHz, Chloroform-*d*) δ 7.51 (m, 2H), 7.41 (m, 3H), 4.58 (m, 1H), 3.47 (s, 2H), 2.72 (m, 1H), 2.45 – 2.31 (m, 2H), 2.12 (m, 4H), 1.92 (m, 2H), 1.79 (m, 2H). ^{13}C NMR (151 MHz, Chloroform-*d*) δ 218.66, 171.26, 137.08, 130.31, 128.40, 127.51, 59.30, 50.79, 50.55, 42.61, 41.42, 41.17, 38.45, 27.92, 26.67,

25.61, 25.16. HRMS (ESI): m/z Calculated for $C_{16}H_{19}NNaO_2^+$ [M+Na$^+$]: 280.1308, found 280.1313.

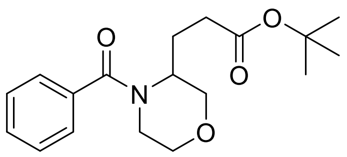

3d: ^{1}H NMR (600 MHz, Chloroform-*d*) δ 7.44 – 7.36 (m, 5H), 4.86 – 3.97 (m, 1H), 4.07 – 2.96 (m, 6H), 2.52 – 1.85 (m, 4H), 1.39 (s, 9H). ^{13}C NMR (151 MHz, Chloroform-*d*) δ 172.36, 170.71, 135.76, 129.83, 128.72, 126.95, 80.68, 69.52, 67.23, 48.64, 43.67, 32.18, 28.16, 24.31. HRMS (ESI): m/z Calculated for $C_{18}H_{25}NNaO_4^+$ [M+Na$^+$]: 342.1676, found 342.1682.

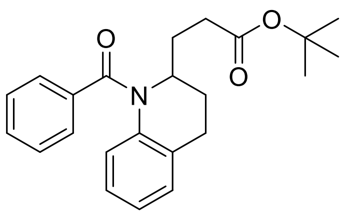

3e: ^{1}H NMR (600 MHz, Chloroform-*d*) δ 7.31 – 7.19 (m, 5H), 7.17 – 7.12 (m, 1H), 7.02 – 6.97 (m, 1H), 6.83 (m, 1H), 6.51 (s, 1H), 4.88 (m, 1H), 2.78 (m, 2H), 2.47 – 2.12 (m, 5H), 1.96 – 1.82 (m, 1H), 1.43 (s, 9H). ^{13}C NMR (151 MHz, Chloroform-*d*) δ 172.68, 170.00, 138.02, 136.45, 129.94, 128.66, 128.58, 127.99, 127.96, 126.91, 126.03, 125.19, 51.95, 42.27, 32.28, 29.79, 28.84, 28.17, 25.17. HRMS (ESI): m/z Calculated for $C_{23}H_{27}NNaO_3^+$ [M+Na$^+$]: 388.1883, found 388.1882.

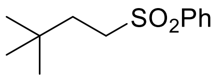

4a: ^{1}H NMR (600 MHz, Chloroform-*d*) δ 7.95 – 7.89 (m, 2H), 7.69 – 7.65 (m, 1H), 7.58 (t, *J* = 7.8 Hz, 2H), 3.09 – 3.04 (m, 2H), 1.62 – 1.58 (m, 2H), 0.87 (s, 9H). ^{13}C NMR (151 MHz, Chloroform-*d*) δ 139.31, 133.69, 129.34, 128.10, 53.02, 35.71, 30.09, 28.98. HRMS (ESI): m/z Calculated for $C_{12}H_{18}NaO_2S^+$ [M+Na$^+$]: 249.0920, found 249.0934.

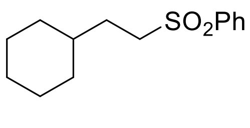

4b: ^{1}H NMR (600 MHz, Chloroform-*d*) δ 7.95 – 7.85 (m, 2H), 7.66 (t, *J* = 7.5 Hz, 1H), 7.57 (t, *J* = 7.7 Hz, 2H), 3.12 – 3.06 (m, 2H), 1.70 – 1.58 (m, 8H), 1.20 – 1.06 (m, 3H), 0.86 (m, 2H). ^{13}C NMR (151 MHz, Chloroform-*d*) δ 133.67, 129.33, 128.12, 54.46, 36.71, 32.85, 29.69, 26.33, 26.06. HRMS (ESI): m/z Calculated for $C_{14}H_{20}NaO_2S^+$ [M+Na$^+$]: 275.1076, found 275.1090.

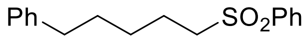

4c: ^{1}H NMR (600 MHz, Chloroform-*d*) δ 7.92 – 7.87 (m, 2H), 7.66 (t, *J* = 7.4 Hz, 1H), 7.57 (t, *J* = 7.8 Hz, 2H), 7.25

(s, 2H), 7.17 (t, *J* = 7.3 Hz, 1H), 7.14 – 7.10 (m, 2H), 3.11 – 3.02 (m, 2H), 2.57 (t, *J* = 7.7 Hz, 2H), 1.76 – 1.71 (m, 2H), 1.60 (d, *J* = 7.4 Hz, 2H), 1.38 (m, 2H). ^{13}C NMR (151 MHz, Chloroform-*d*) δ 142.03, 139.25, 133.73, 129.36, 128.43, 128.42, 128.14, 125.93, 56.30, 35.54, 30.87, 27.88, 22.63. HRMS (ESI): m/z Calculated for $C_{17}H_{20}NaO_2S^+$ [M+Na$^+$]: 311.1076, found 311.1096.

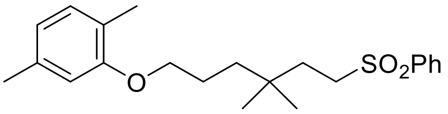

4d: ^{1}H NMR (600 MHz, Chloroform-*d*) δ 7.91 (d, *J* = 7.7 Hz, 2H), 7.66 (t, *J* = 7.5 Hz, 1H), 7.57 (t, *J* = 7.6 Hz, 2H), 7.00 (d, *J* = 7.4 Hz, 1H), 6.66 (d, *J* = 7.4 Hz, 1H), 6.59 (s, 1H), 3.86 (t, *J* = 6.1 Hz, 2H), 3.10 – 3.02 (m, 2H), 2.30 (s, 3H), 2.14 (s, 3H), 1.68 – 1.63 (m, 4H), 1.38 – 1.28 (m, 2H), 0.88 (s, 6H). ^{13}C NMR (151 MHz, Chloroform-*d*) δ 156.99, 139.25, 136.61, 133.76, 130.42, 129.39, 128.12, 123.55, 120.83, 112.01, 68.09, 52.53, 37.79, 33.47, 32.36, 26.87, 24.13, 21.50, 15.90. HRMS (ESI): m/z Calculated for $C_{22}H_{30}NaO_3S^+$ [M+Na$^+$]: 397.1808, found 397.1823.

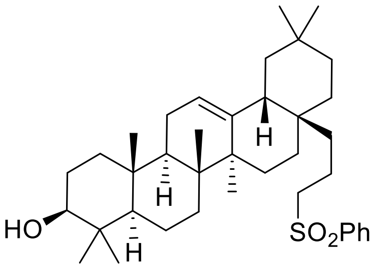

4e: ^{1}H NMR (600 MHz, Chloroform-*d*) δ 7.91 – 7.86 (m, 2H), 7.67 – 7.62 (m, 1H), 7.56 (t, *J* = 7.8 Hz, 2H), 5.16 (t, *J* = 3.7 Hz, 1H), 3.20 (m, 1H), 3.07 (m, 1H), 2.97 (m, 1H), 1.90 (m, 3H), 1.81 (m, 2H), 1.66 (d, *J* = 13.6 Hz, 1H), 1.64 – 1.37 (m, 9H), 1.38 – 1.09 (m, 11H), 1.05 – 0.98 (m, 4H), 0.95 – 0.88 (m, 5H), 0.86 (s, 3H), 0.83 (s, 3H), 0.79 (s, 3H), 0.70 (dd, *J* = 11.8, 1.9 Hz, 1H), 0.61 (s, 3H). ^{13}C NMR (151 MHz, Chloroform-*d*) δ 133.63, 129.34, 128.13, 123.08, 79.03, 55.19, 51.60, 47.58, 46.85, 46.50, 39.70, 38.84, 38.66, 36.94, 34.63, 34.25, 33.21, 32.87, 32.46, 31.88, 30.94, 28.16, 27.27, 26.11, 25.48, 23.57, 23.30, 18.35, 16.46, 15.68, 15.56. HRMS (ESI): m/z Calculated for $C_{38}H_{58}NaO_3S^+$ [M+Na$^+$]: 617.3999, found 617.3993.

4. Mechanistic Study

4.1 DFT simulation

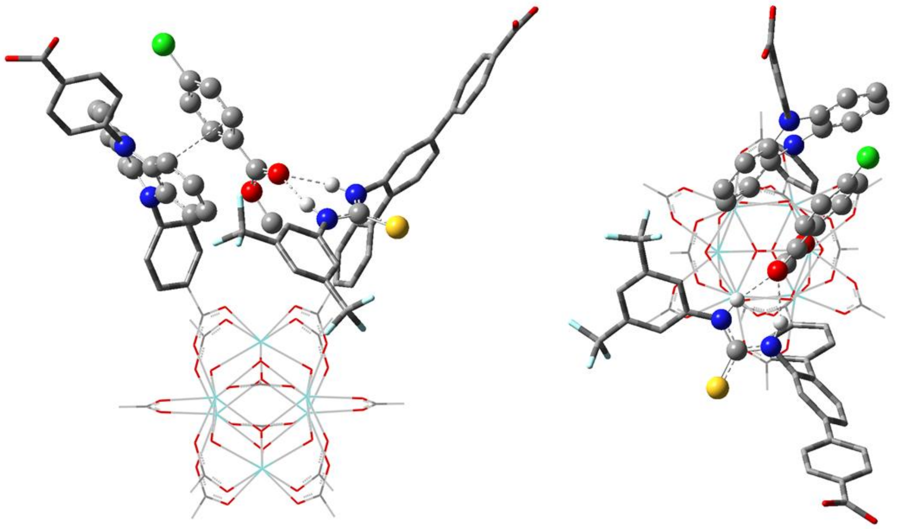

Figure S43. DFT/semi-empirical computation of non-covalent interaction between methyl 4-chlorobenzoate and **MOF-F-TU** (left: side view, right: top view).

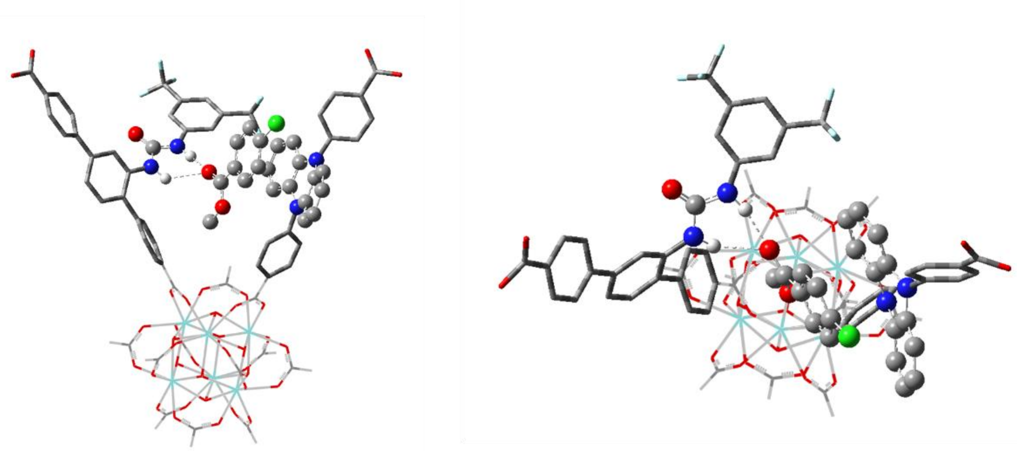

Figure S44. DFT/semi-empirical computation of non-covalent interaction between methyl 4-chlorobenzoate and **MOF-F-U** (left: side view, right: top view).

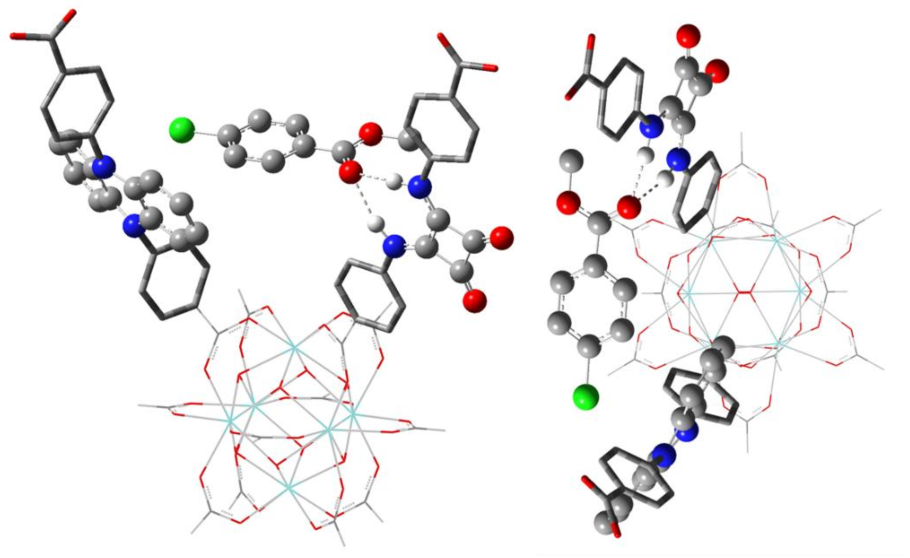

Figure S45. DFT/semi-empirical computation of non-covalent interaction between methyl 4-chlorobenzoate and **MOF-R-SQA1** (left: side view, right: top view).

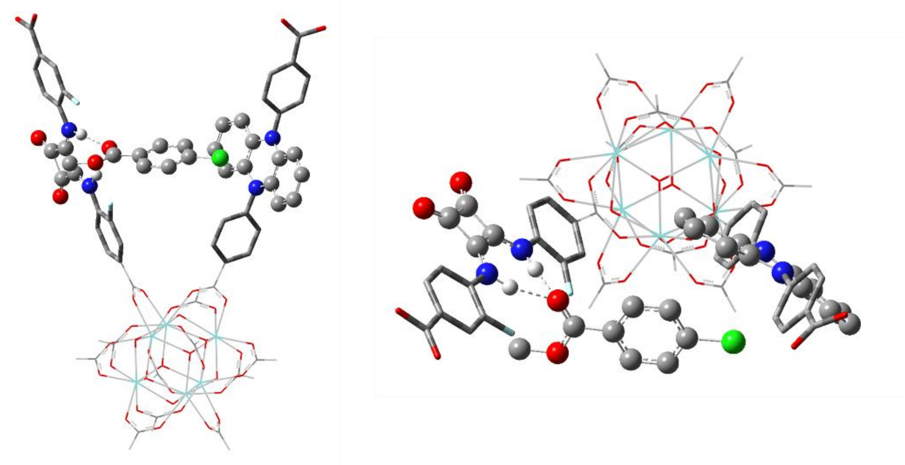

Figure S46. DFT/semi-empirical computation of non-covalent interaction between methyl 4-chlorobenzoate and **MOF-R-SQA2** (left: side view, right: top view).

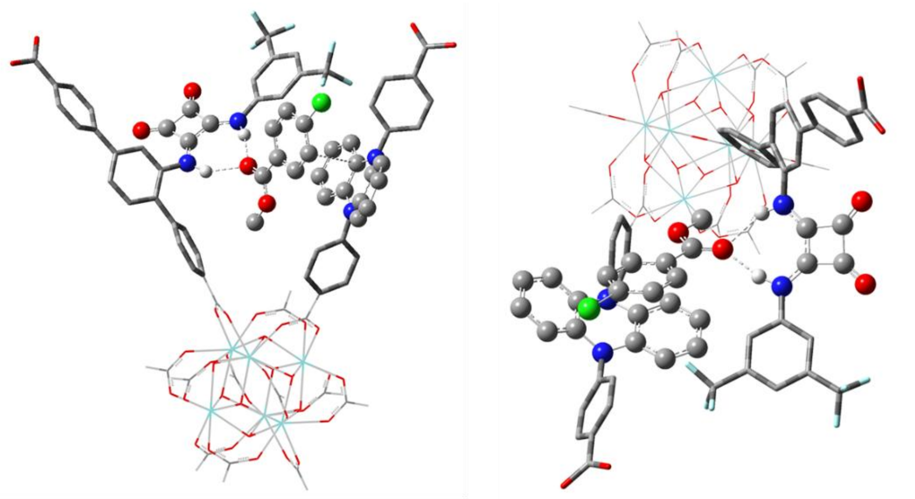

Figure S47. DFT/semi-empirical computation of non-covalent interaction between methyl 4-chlorobenzoate and **MOF-F-SQA** (left: side view, right: top view).

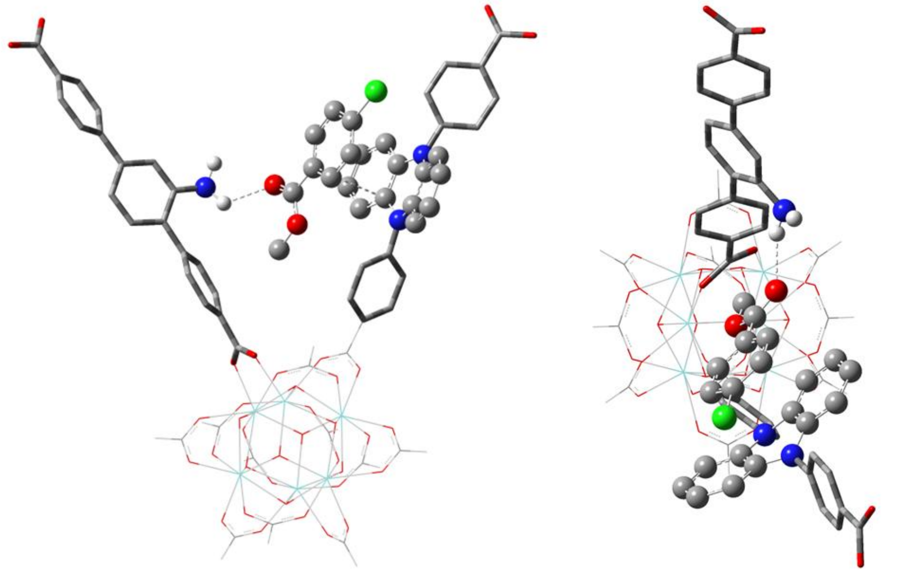

Figure S48. DFT/semi-empirical computation of non-covalent interaction between methyl 4-chlorobenzoate and **MOF-NH$_2$** (left: side view, right: top view).

The structures were optimized using density functional theory (DFT)/semi-empirical (SE) model. The ligands and substrate were simulated using DFT, and the Zr_6 SBU was extracted from single crystal XRD result and fixed during structure optimization. All other sites on Zr_6 SBU were replaced with formic acid. The DFT part was computed using the M06-2x functional[3] and def2-TZVP basis set[4] for S, Cl atoms, 6-31G(d,p)

basis set for other atoms. In the SE part, the wavefunction was generated using PM6 method[5].

All computation was carried out using Gaussian 16 software[6], visualization was produced from GaussView 6 software[7]. The coordinate file was attached as a separate .xyz document.

4.2 Control experiments

Table S1. Control experiments using MOF catalysts with different component ratios for dehalogenation.[a]

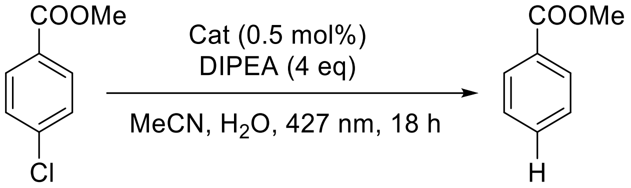

Entry	Catalyst	Yield (%)[b]
1	**MOF-F-TU(2:1)**	68
2	**MOF-F-TU(1:1)**	80
3	**MOF-F-TU(1:2)**	99
4	**MOF-R-SQA1(2:1)**	ND
5	**MOF-R-SQA1(1:1)**	ND
6	**MOF-R-SQA1(1:2)**	ND

[a]Standard conditions: Methyl 4-chlorobenzoate (34.0 mg, 0.2 mmol), DIPEA (103.4 mg, 0.8 mmol), **MOF-F-TU** or **MOF-R-SQA1** (0.5 mol% based on the PZDB site), and MeCN (2 mL), blue LED (427 nm, 30 W), N_2 atmosphere, rt, 18 h. [b] GC yields.

Table S2. Control experiments with varied conditions for dehalogenation.[a]

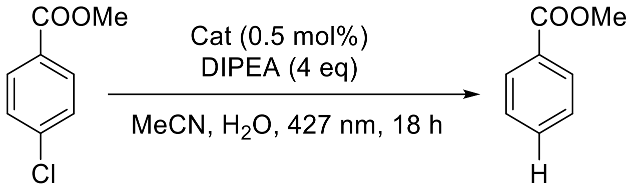

Entry	Standard Condition	Yield (%)[b]
1	**-**	99
2	**MOF-R-SQA1(1:2)** instead of **MOF-F-TU(1:2)**	ND
3	**MOF-R-SQA2(1:2)** instead of **MOF-F-TU(1:2)**	ND
4	**MOF-F-U(1:2)** instead of **MOF-F-TU(1:2)**	72
5	**MOF-F-SQA(1:2)** instead of **MOF-F-TU(1:2)**	62
6	**MOF-NH$_2$(1:2)** instead of **MOF-F-TU(1:2)**	11
7	**Zr-PZDB** instead of **MOF-F-TU(1:2)**	ND
8	**L-PZDB-Me/L-TU-Me (1:2)** instead of **MOF-F-TU(1:2)**	8
9	**TU-1**	ND
10	**Zr-PZDB/TU-1 (1:2)** instead of **MOF-F-TU(1:2)**	41
11	**L-PZDB-Me/TU-1 (1:2)** instead of **MOF-F-TU(1:2)**	44
12	**L-PZDB-Me/L-SQA-R1-H (1:2)** instead of **MOF-F-TU(1:2)**	32
13	**DPPZ** instead of **MOF-F-TU(1:2)**	22
14	**DPPZ/TU-1 (1:2)** instead of **MOF-F-TU(1:2)**	56
15	**L-PZDB-Me** instead of **MOF-F-TU(1:2)**	ND
16	**L-DHBDs** instead of **MOF-F-TU(1:2)**	ND
17	**No light**	ND

18	**No catalyst**	ND

[a]Standard conditions: Methyl 4-chlorobenzoate (34.0 mg, 0.2 mmol), DIPEA (103.4 mg, 0.8 mmol), catalyst (0.5 mol% based on the PZDB, the ratio between PZDB and DHBD is 1:2 if both are used), and MeCN (2 mL), blue LED (427 nm, 30 W), N_2 atmosphere, rt, 18 h; For other catalysts, 0.5 mol% loading is based on the PC site, the ratio between PC and DHBD is 1:2. [b] GC yields.

Table S3. Control experiments with different catalysts for dehalogenative arylation.[a]

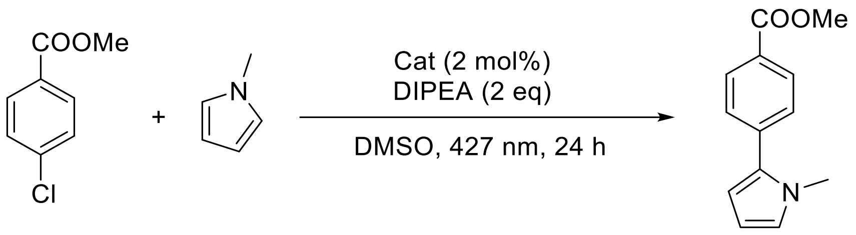

Entry	Catalyst	Yield (%)[b]
1	**MOF-F-TU(1:2)**	90
2	**Zr-PZDB**	ND
3	**MOF-R-SQA1(1:2)**	ND
4	**L-PZDB-Me**	ND
5	**L-PZDB-Me/TU-1 (1:2)**	< 5
6	**L-PZDB-Me/ L-SQA-R1-Me (1:2)**	< 5

[a]Standard conditions: Methyl 4-chlorobenzoate (17.0 mg, 0.1 mmol), *N*-methyl pyrrole (324.5 mg, 4 mmol), DIPEA (25.8 mg, 0.2 mmol), catalyst (2 mol% based on the PZDB, the ratio between PZDB and DHBD is 1:2 if both are used), and DMSO (1 mL), blue LED (427 nm, 30 W), N_2 atmosphere, rt, 24 h. [b] isolated yields.

Table S4. Control experiments with different catalysts for remote C-H alkylation.[a]

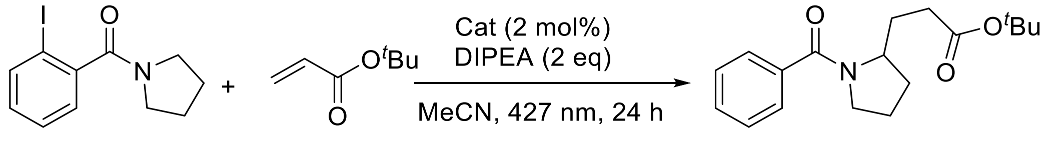

Entry	Catalyst	Yield (%)[b]
1	**MOF-F-TU(1:2)**	82
2	**Zr-PZDB**	33
3	**MOF-R-SQA1(1:2)**	26
4	**L-PZDB-Me**	28
5	**L-PZDB-Me + TU-1 (1:2)**	60
6	**L-PZDB-Me + L-SQA-R1-Me (1:2)**	45

[a]Standard conditions: 1-(2-iodobenzoyl) pyrrolidine (30.0 mg, 0.1 mmol), tert-butyl acrylate (0.2 mmol, 25.6 mg), DIPEA (25.8 mg, 0.2 mmol), catalyst (2 mol% based on the PZDB, the ratio between PZDB and DHBD is 1:2 if both are used), and MeCN (1 mL), blue LED (427 nm, 30 W), N_2 atmosphere, rt, 24 h. [b] Isolated yields.

Table S5. Control experiments with different catalysts for decarboxylative alkylation.[a]

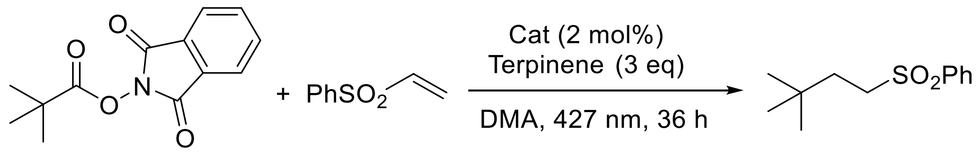

Entry	Catalyst	Yield (%)[b]
1	**MOF-F-TU(1:2)**	95
2	**Zr-PZDB**	55
3	**MOF-R-SQA1(1:2)**	43
4	**L-PZDB-Me**	52
5	**L-PZDB-Me/TU-1 (1:2)**	93
6	**L-PZDB-Me/L-SQA-R1-Me (1:2)**	90

[a]Standard conditions: Methyl 4-chlorobenzoate (24.7 mg, 0.1 mmol), phenyl vinyl sulfone (33.6 mg, 0.2 mmol), γ-terpinene (44.9 mg, 0.3 mmol), catalyst (2 mol%

based on the PZDB, the ratio between PZDB and DHBD is 1:2 if both are used), and DMA (1 mL), blue LED (427 nm, 30 W), N_2 atmosphere, rt, 36 h. [b] Isolated yields.

4.3 Light on-off experiments

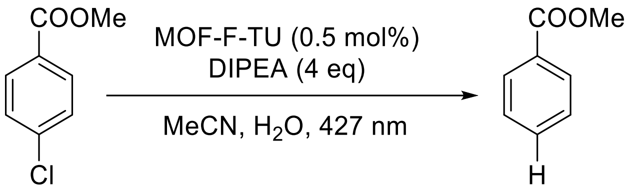

In a N_2-filled glovebox, methyl 4-chlorobenzoate (34.0 mg, 0.2 mmol), DIPEA (103.4 mg, 0.8 mmol), **MOF-F-TU** (1 mg, 0.5 mol% based on the PZDB site), and MeCN (2 mL) were loaded into a 10-mL reaction tube equipped with a magnetic stirring bar. The tube was then taken out from the glovebox, to which was added 0.2 mL degassed H_2O. After being tightly screw-caped, the reaction mixture was subsequently stirred under irradiation (Kessil PR160L-427) at room temperature for 1 hour. A proportion (20 µl) of reaction mixture was taken out and then subjected to GC-MS analysis. After that, the left mixture was stirred under dark for 1 hour. Another proportion (20 µl) of reaction mixture was taken out and subjected to GC-MS analysis. The same light on/off procedure repeated four more times. The results were compiled in Figure S49, indicating no reaction proceeding in the dark.

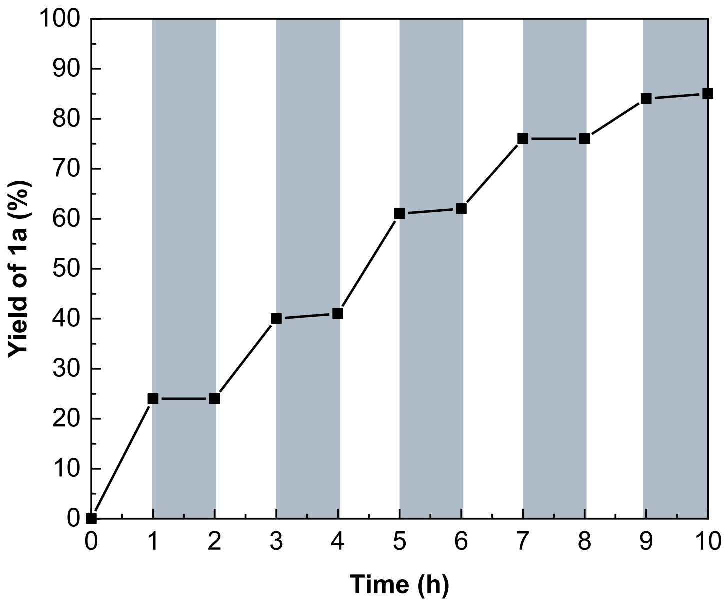

Figure S49. Yields of **1a** for light on/off experiments.

4.4 Radical Capture Experiments

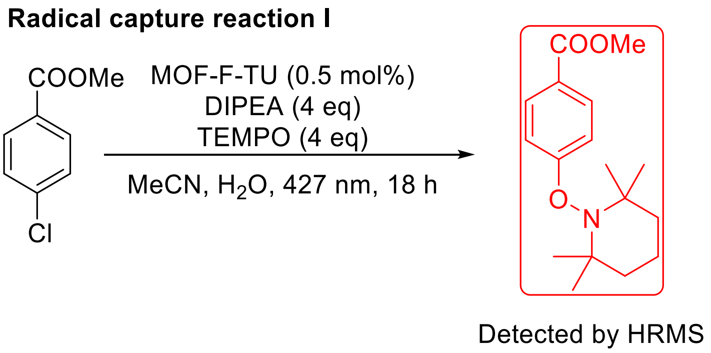

In a N_2-filled glovebox, methyl 4-chlorobenzoate (34.0 mg, 0.2 mmol), DIPEA (103.4 mg, 0.8 mmol), TEMPO (62.5 mg, 0.4 mmol), **MOF-F-TU** (1 mg, 0.5 mol% based on the PZDB site), and MeCN (2 mL) were added into a reaction tube equipped with a magnetic stirring bar. The tube was then taken out from the glovebox, to which was added 0.2 mL degassed H_2O. After being tightly screw-caped, the tube was subsequently stirred under irradiation (Kessil PR160L-427) at room temperature for 18 hours. Afterward, the MOF catalyst was separated by centrifugation, and the

supernatant was subjected to HRMS analyses. HRMS (ESI): m/z Calculated for $C_{17}H_{25}NO_3$ [M^+]: 291.1834, found 291.1893.

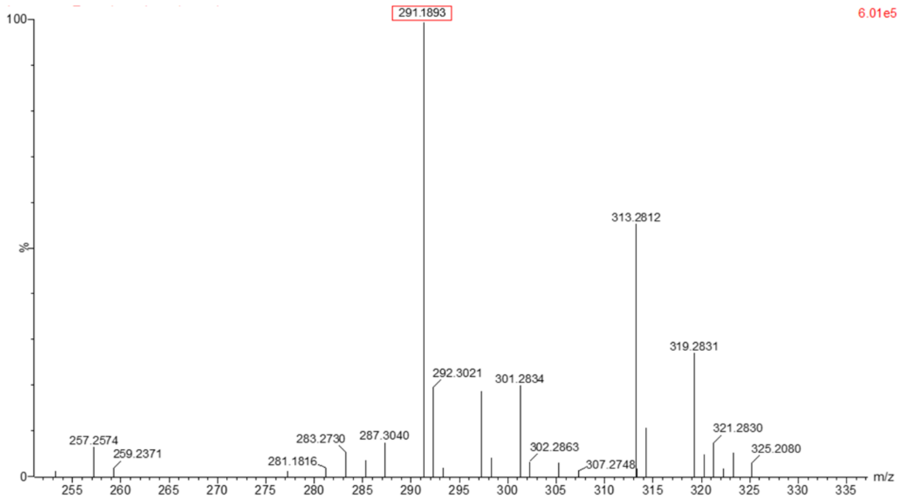

Figure S50. HRMS spectrum of the mixture of radical capture reaction I.

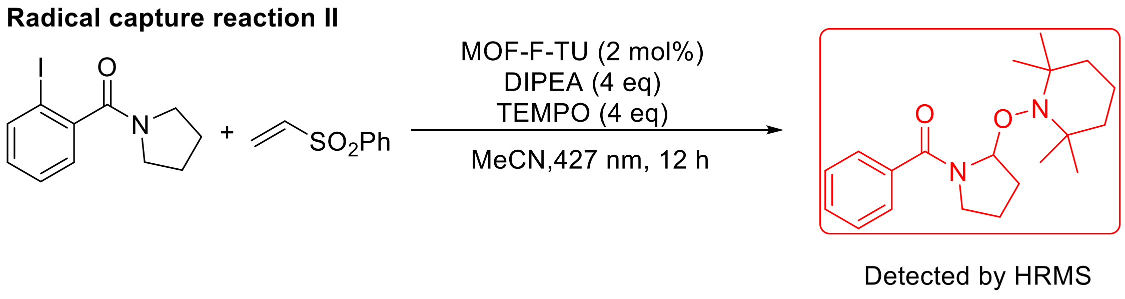

In a N_2-filled glovebox, aryl halide (30.0 mg, 0.1 mmol), DIPEA (51.7 mg, 0.4 mmol), Micheal acceptor (33.6 mg, 0.2 mmol), TEMPO (62.5 mg, 0.4 mmol), **MOF-F-TU** (2 mg, 2 mol% based on the PZDB site), and MeCN (1 mL) were loaded into a 4-mL reaction tube equipped with a magnetic stirring bar. After being tightly screw-caped, the tube was then taken out from the glovebox and was subsequently stirred under irradiation (Kessil PR160L-427) at room temperature for 12 hours. Afterward, the MOF catalyst was separated by centrifugation, and the supernatant was subjected to HRMS analyses. HRMS (ESI): m/z Calculated for $C_{20}H_{30}N_2NaO_2$ [$M+Na^+$]: 353.2199, found 353.2192.

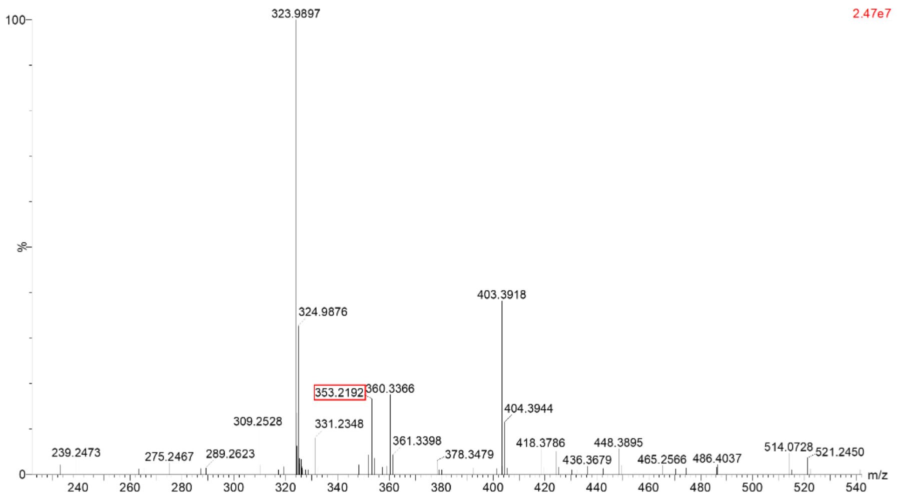

Figure S51. HRMS spectrum of the mixture of radical capture reaction II.

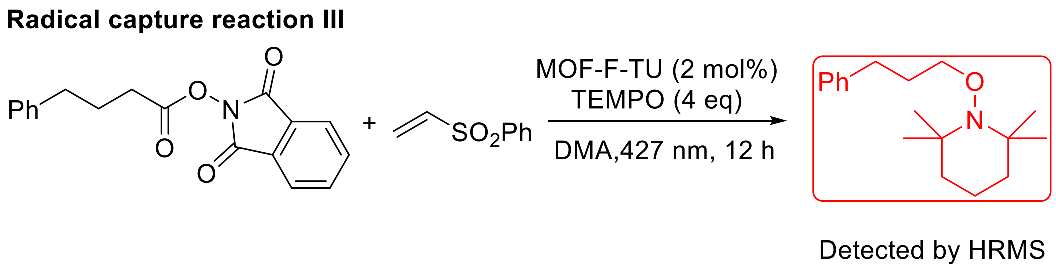

In a N_2-filled glovebox, NHPI ester (30.9 mg, 0.1 mmol), Micheal acceptor (33.6 mg, 0.2 mmol), γ-terpinene (44.9 mg, 0.3 mmol), **MOF-F-TU** (2 mg, 2 mol% based on the PZDB), and MeCN (1 mL) were loaded into a 4-mL reaction tube equipped with a magnetic stirring bar. After being tightly screw-caped, the tube was then taken out from the glovebox and subsequently stirred under irradiation (Kessil PR160L-427) at room temperature for 12 hours. Afterward, the MOF catalyst was separated by centrifugation, and the supernatant was subjected to HRMS analyses. HRMS (ESI): m/z Calculated for $C_{18}H_{29}NO$ [M^{+}]: 275.2249, found 275.2286.

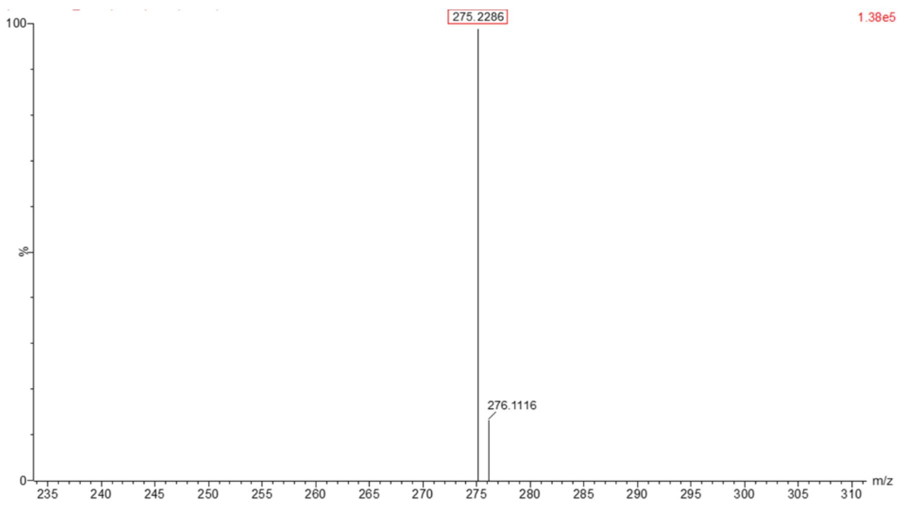

Figure S52. HRMS spectrum of the mixture of radical capture reaction III.

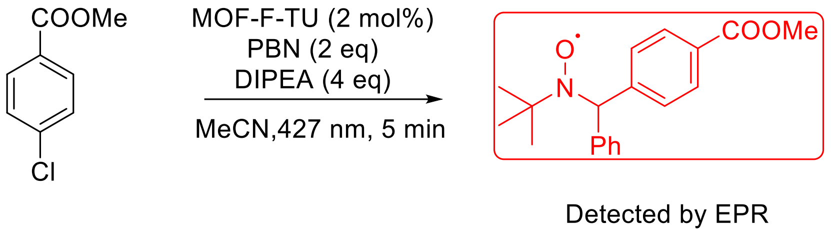

In a N_2-filled glovebox, methyl 4-chlorobenzoate (34.0 mg, 0.2 mmol), DIPEA (103.4 mg, 0.8 mmol), PBN (62.5 mg, 0.4 mmol), **MOF-F-TU** (1 mg, 0.5 mol% based on the PZDB site), MeCN (2 mL) and 0.2 mL degassed H_2O were added into a EPR test tube. The tube was then taken out of the glovebox. After being tightly screw-capped, the tube was used to collect the background signal and then irradiated for 5 minutes (Kessil PR160L-427) to detect the signal of the radical species.

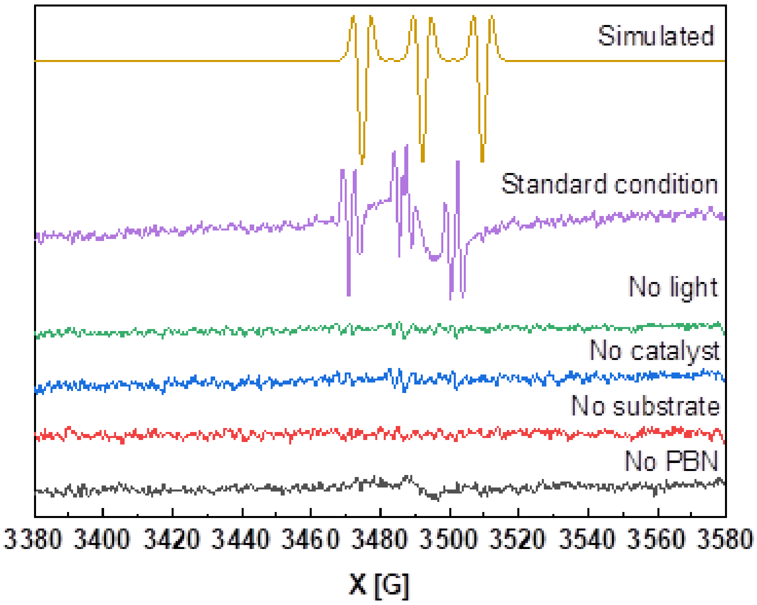

Figure S53. EPR tests under different reaction conditions. Fitted parameters: experimental g = 2.006, simulated g = 2.002; experimental a_N = 15.1 G, a_H = 3.5 G; simulated a_N = 17.6 G, a_H = 5.3 G.

4.5 Recycle Test

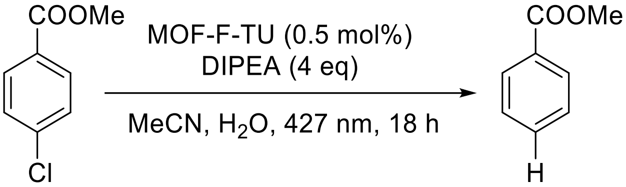

In a N_2-filled glovebox, methyl 4-chlorobenzoate (34.0 mg, 0.2 mmol), DIPEA (103.4 mg, 0.8 mmol), **MOF-F-TU** (1 mg, 0.5 mol% based on the PZDB site), and MeCN (2 mL) were loaded into a 10-mL reaction tube equipped with a magnetic stirring bar. The tube was then taken out from the glovebox, to which was added 0.2 mL degassed H_2O. After being tightly screw-caped, the reaction mixture was subsequently stirred under irradiation (Kessil PR160L-427) at room temperature for 18 hours. Afterward, the MOF catalyst was separated from the reaction mixture by centrifugation in the glovebox, washed with MeCN (3 x 2 mL), and directly used for the next catalytic cycle without the need for activation or drying. The corresponding supernatant of the reaction mixture was evaporated under vacuum and subjected to GC-MS analysis for each run.

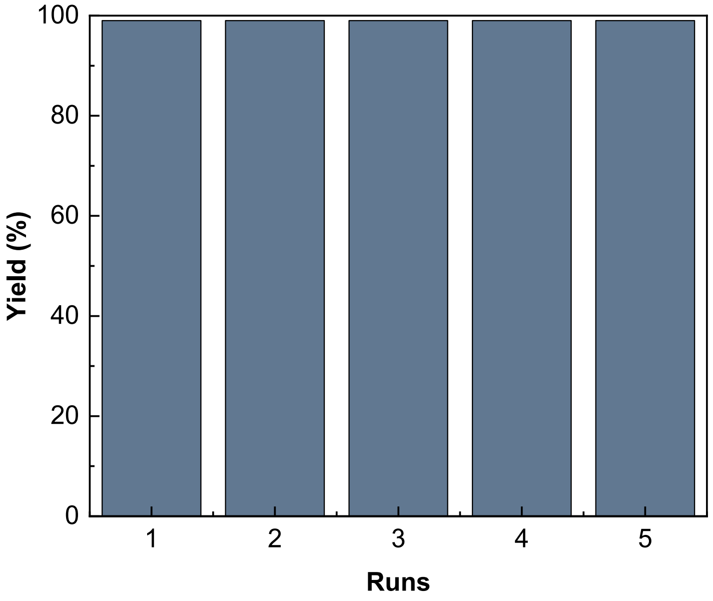

Figure S54. Yields of **1a** for recycle experiments.

4.6 Fluorescence Quenching Experiments

4.6.1 Luminescence quenching of DPPZ (5,10-diphenyl-5,10-dihydrophenazine) by S1 (methyl 4-chlorobenzoate).

In a N_2-filled glovebox, 2 mL CH_3CN solution of DPPZ (10^{-4} M) was added to a 4 mL cuvette. Different amounts of S1 (5, 10, 20, 50, and 100 eq) were then added into the solution, respectively. The luminescence intensity of DPPZ in each of these samples was measured separately and the results were used to plot a Stern-Volmer curve (Figure S55).

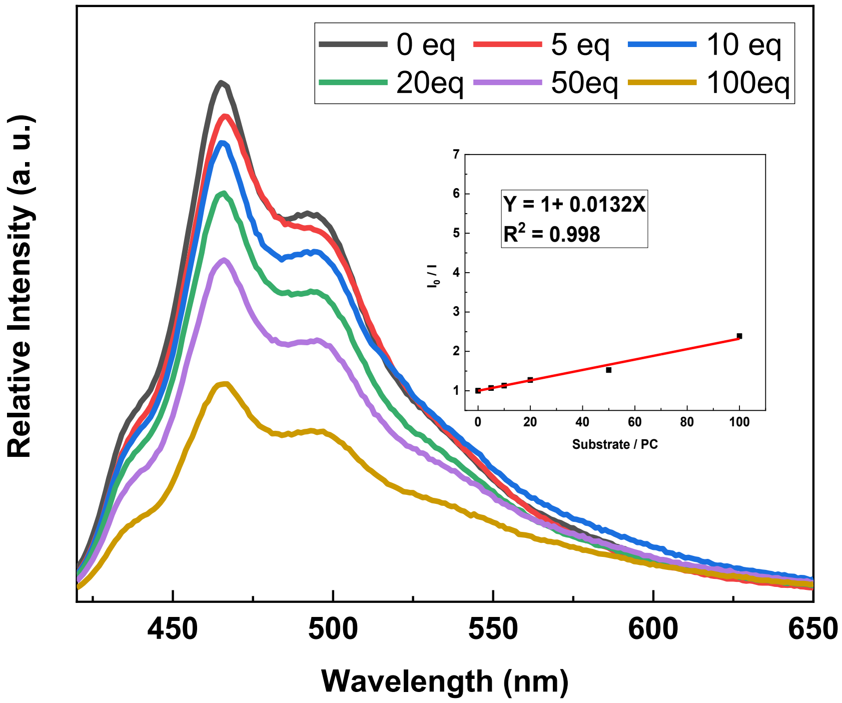

Figure S55. Luminescence intensity of DPPZ when quenched by S1; insert: the Stern-Volmer curve.

4.6.2. Luminescence quenching of DPPZ by a mixture of S1 and TU-1 (1,3-bis[3,5-bis(trifluoromethyl)phenyl] thiourea) (1:1).

In a N_2-filled glovebox, 2 mL CH_3CN solution of DPPZ (10^{-4} M) was added to a 4 mL cuvette. Different amounts of the mixture of S1 and TU-1 (5, 10, 20, 50, and 100 eq) were then added into the solution, respectively. The luminescence intensity of DPPZ in each of these samples was measured separately and the results were used to plot a Stern-Volmer curve (Figure S56).

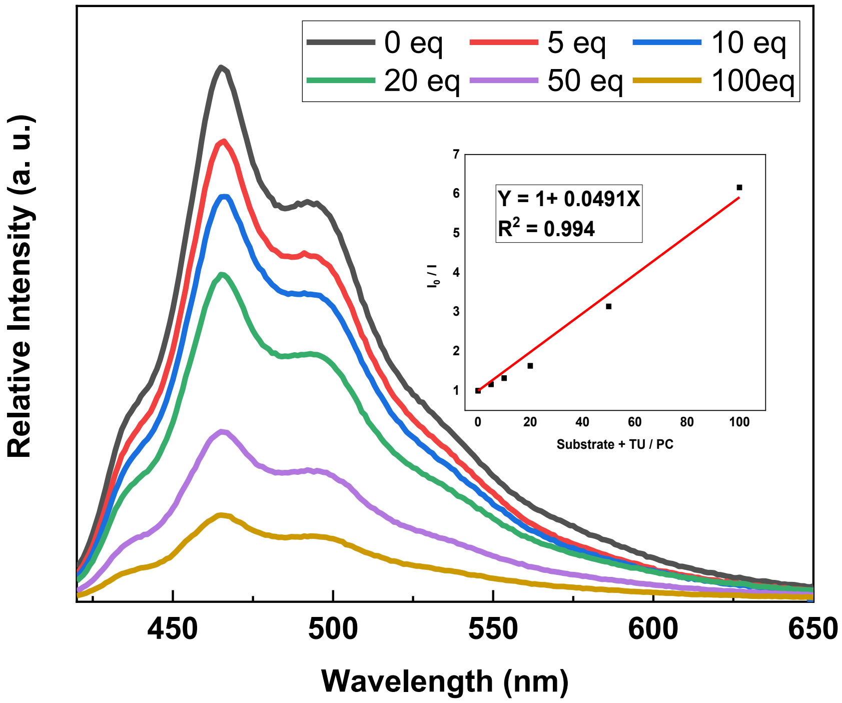

Figure S56. Luminescence intensity of DPPZ when quenched by a mixture of S1 and TU-1; insert: the Stern-Volmer curve.

4.6.3 Luminescence quenching of DPPZ by other reagents.

In a N_2-filled glovebox, 2 mL CH_3CN solution of DPPZ (10^{-4} M) was added to a 4 mL cuvette. Different reagents (100 eq), including TU-1 and DIPEA, were then added into the solution, respectively. The resultant mixtures were then subjected to luminescence measurements. The corresponding results indicated no luminescence quenching of DPPZ by these reagents being observed.

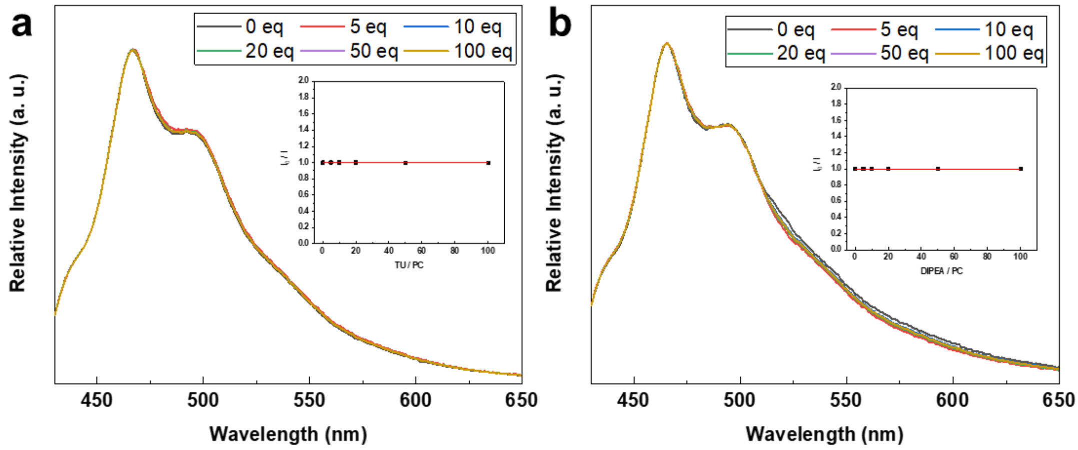

Figure S57. Luminescence intensity of DPPZ when quenched by TU-1 (a) and DIPEA (b).

4.7 TA measurements

DPPZ (100 μM) were dissolved in acetonitrile and MOF suspension (0.1 mg·mL^{-1}) were prepared in acetonitrile in the absence and presence of **S1** (100 mM). The prepared samples were then transferred into a 2 mm pathlength quartz cuvette, sealed with a septum cap and degassed by argon bubbling for 15 minutes prior to TA measurements. Each TA measurement was averaged 3-5 times to increase the signal-to-noise ratio. Global lifetime analysis (GLA) was carried out within Surface Xplorer software. GLA was used to provide a way to visualise complex data sets of time-resolved spectra by decomposing them into a small number of compartments and to examine the time dependence. Here we used a sequential evolution GLA approach where the TA data were fitted into 3 sequentially excited compartments ($A_1 \rightarrow A_2 \rightarrow A_3 \rightarrow$) and the population of each compartment is modelled by a single exponential decay function, which will be convoluted (⊗) by an instrument response function (IRF(t,λ)), Eq 1. The measured TRIR spectrum consists of the sum of the contributions from each pathway.

$$\Delta A\ (t,\lambda) = \sum_{j=1}^{n} x_j(\tau_j,\lambda)\, e^{-t/\tau_j} \otimes IRF(t,\lambda)$$ Equation S-1

Where ΔA is the change in optical density, x_j represents the pre-exponential factor, n is the number of compartments, τ_j is the lifetime for each conponent, λ is the wavelength.

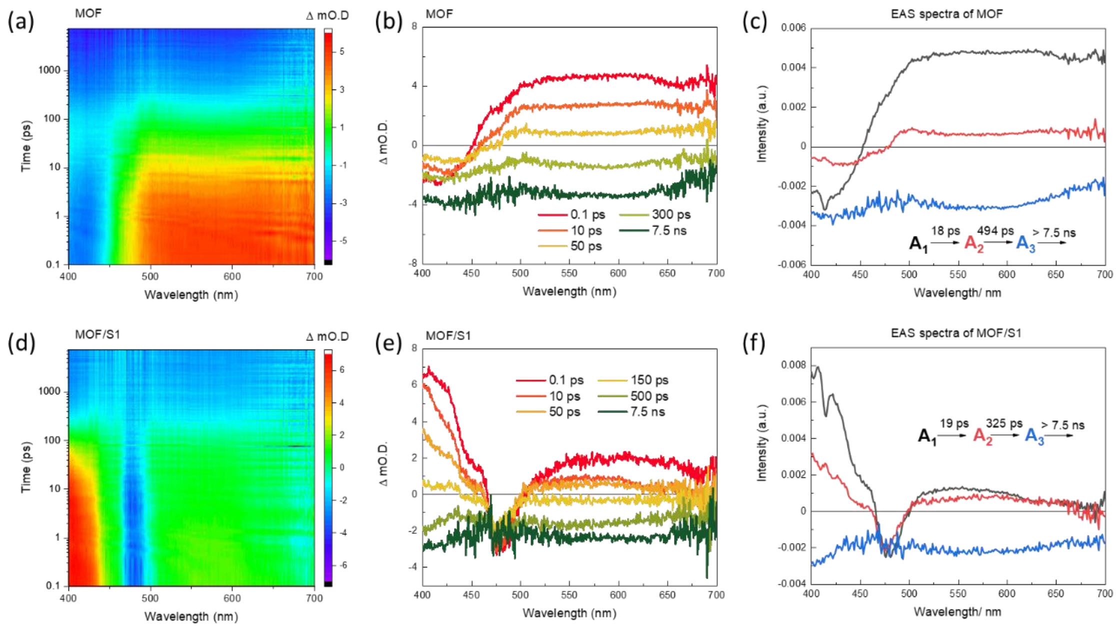

Figure S58. The complete ultrafast transient absorption surface of **MOF-F-TU** (1mg/mL) in the absence (a) and presence (d) of **S1**(100 mM) following 390 nm excitation, and the corresponding TA spectra at selected pump-probe time delays (b,c), and the evolution associated spectra (EAS) and time constants (c,f) generated through 3 compartment sequential pathway global lifetime analysis (GLA) of data in part (a, b) respectively.

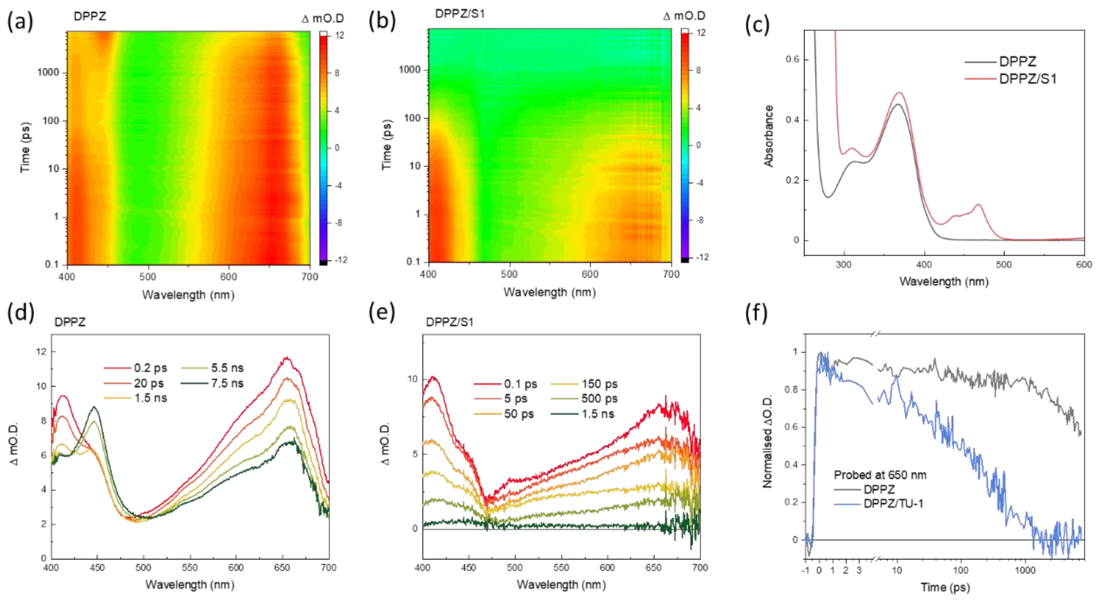

Figure S59. The complete ultrafast transient absorption surface of DPPZ (100µM) in the absence (a) and presence (b) of **S1** (100 mM) following 390 nm excitation, and the corresponding TA spectra at selected pump-probe time delays (d,e), respectively. (c) UV-vis spectra of DPPZ and DPPZ/**S1**. (e) The kinetic traces probed at 650 nm assigned to singlet excited state of DPPZ, showing the ultrafast electron transfer from excited DPPZ to **S1**.

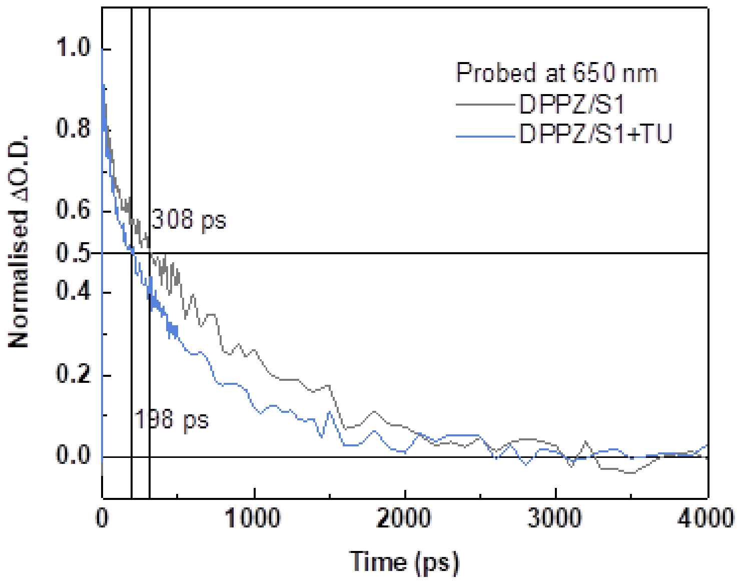

Figure S60. Kinetic traces probed at 650 nm assigned to singlet excited state of DPPZ in the presence of **S1** or a mixture of **S1** and TU. The addition of **S1** significantly

reduced the excited-state lifetime of DPPZ from 10 ns to 444 ps, while using a mixture of **S1** and TU-1 further decreased the lifetime to 286 ps ($\tau = t_{1/2}$ / ln2).

4.8 Deuterium labeling experiment

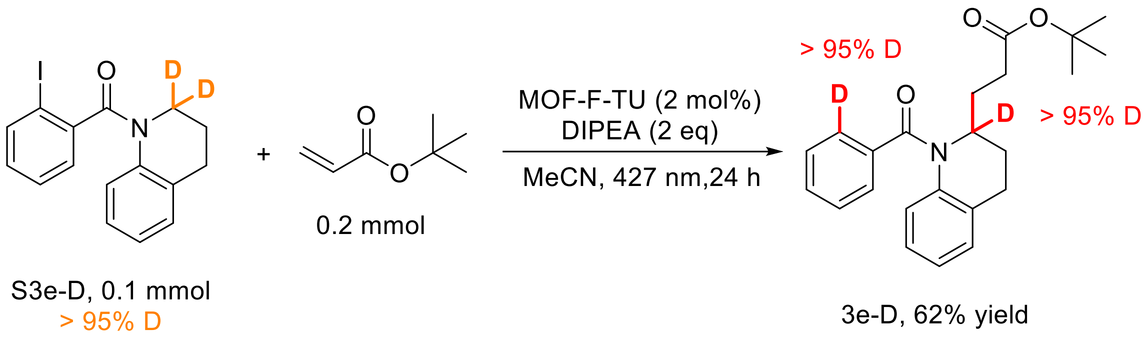

In a N_2-filled glovebox, deuterium labelaryl halide (36.5 mg, 0.1 mmol), DIPEA (25.6 mg, 0.2 mmol), Micheal acceptor (33.6 mg, 0.2 mmol), **MOF-F-TU** (2 mg, 2 mol% based on the PZDB site), and MeCN (1 mL) were loaded into a 4-mL reaction tube equipped with a magnetic stirring bar. After being tightly screw-caped, the tube was then taken out from the glovebox and was subsequently stirred under irradiation (Kessil PR160L-427) at room temperature for 24 hours. After reaction, purification by column chromatography on silica gel was performed to give pure products with isolated yields.

4.9 Analysis of side products

Table S6. Detailed analysis of the control experiment.

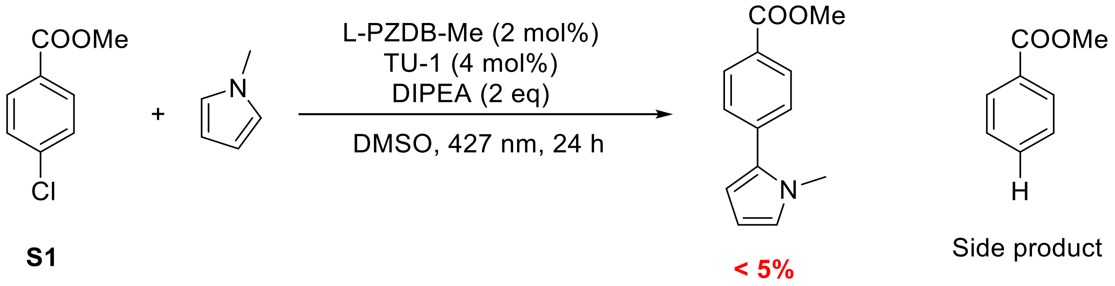

Entry	Compound	Remaining quantity (%)[a]
1	**S1**	90
2	**Side product**	6

3	***N*-methylpyrrole**	> 95
4	**L-PZDB-Me**	ND
5	**TU-1**	80

[a]Detected by GC-MS and ^{1}H NMR.

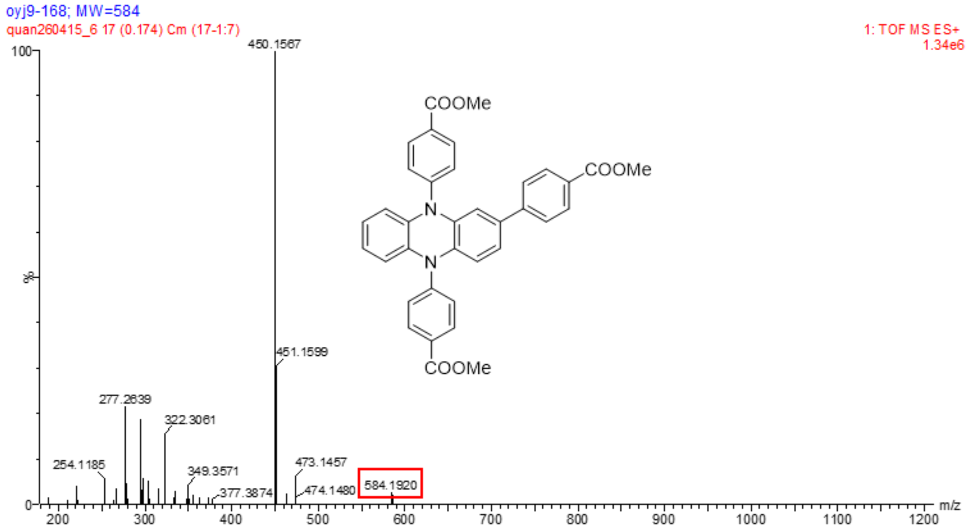

Figure S61. HRMS spectrum of the reaction mixture from dehalogenative arylation reaction.

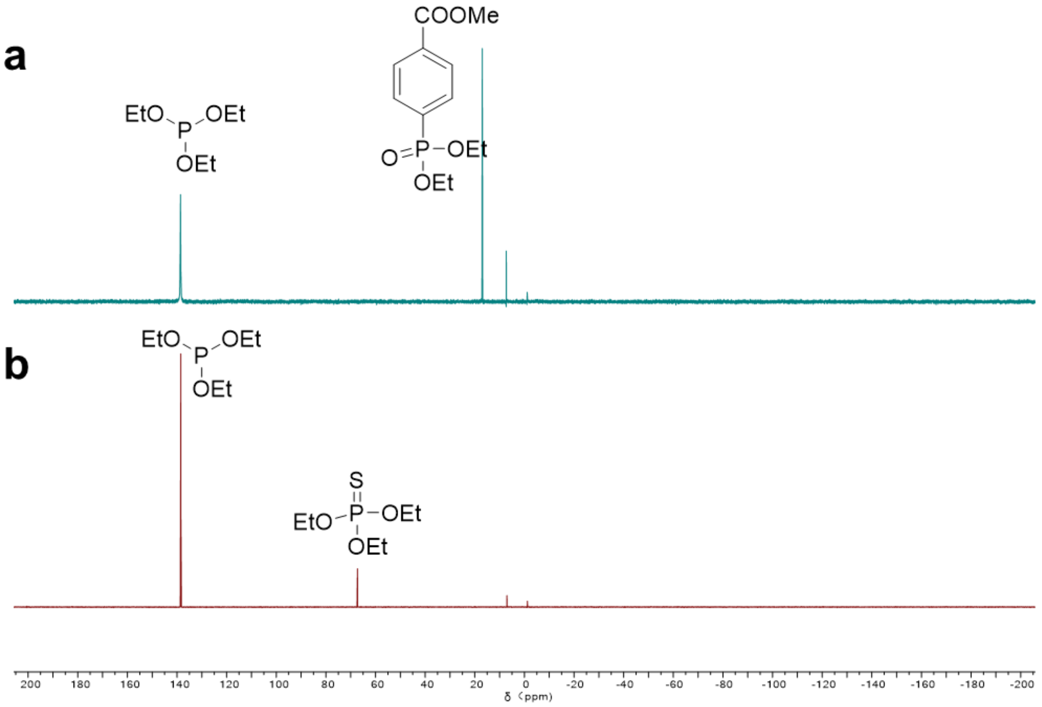

Figure S62. ^{31}P NMR spectra of the reaction mixture from dehalogenative phosphination reaction, a) **MOF-F-TU** as catalyst, b) L-PZDB-Me and TU-1 as catalyst.

4.10 Hot-Filtration Experiments

In a N_2-filled glovebox, methyl 4-chlorobenzoate (34 mg, 0.2 mmol), DIPEA (103.4 mg, 0.8 mmol), **MOF-F-TU** (1 mg, 0.5 mol% based on the PZDB site), and MeCN (2 mL) were added into a reaction tube equipped with a magnetic stirring bar. The tube was then taken out from the glovebox, to which was added 0.2 mL degassed H_2O. After being tightly screw-caped, he reaction mixture was subsequently stirred under irradiation (Kessil PR160L-427) at room temperature for 12 hours. Afterward, the MOF catalyst was separated from the reaction mixture by centrifugation in the glovebox. Half of the supernatant was subjected to GC-MS analysis to identify the yield of **1a**. Another half was stirred under irradiation (427 nm) at room temperature for an additional 12 hours and subsequently subjected to GC-MS analysis to identify the yield of **1a**.

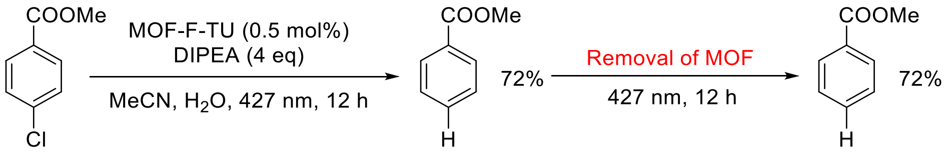

Figure S63. Hot-filtration experiments.

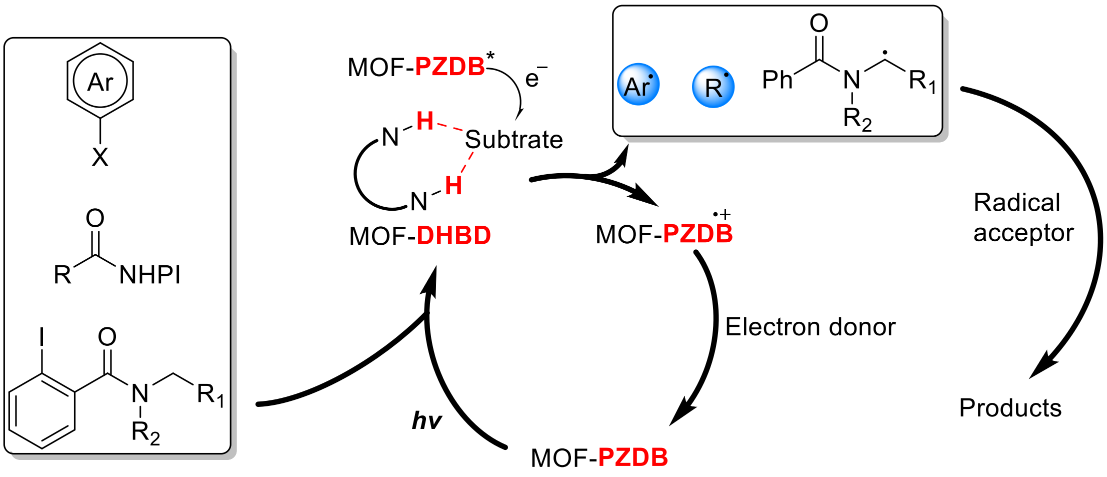

Figure S64. Proposed mechanism.

5. GC-MS Spectra

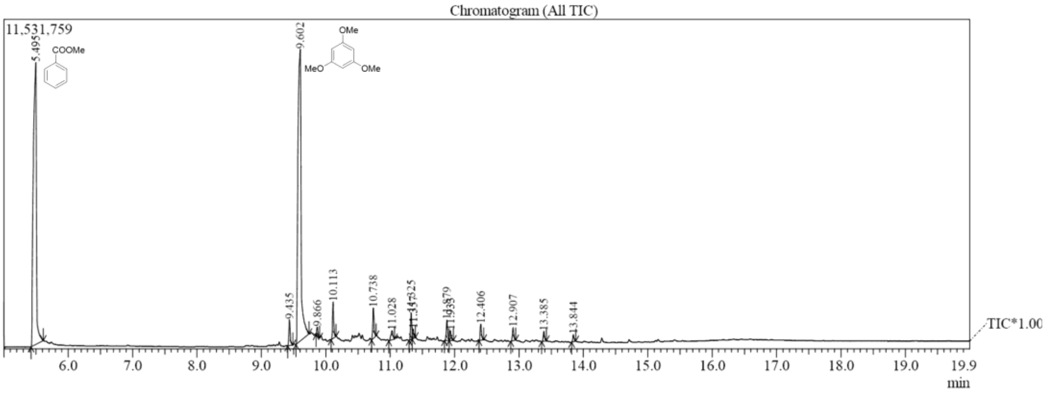

Figure S65. GC-MS spectrum of **1a**, 1,3,5-trimethoxybenzene (16.8 mg, 0.1 mmol) as internal standard.

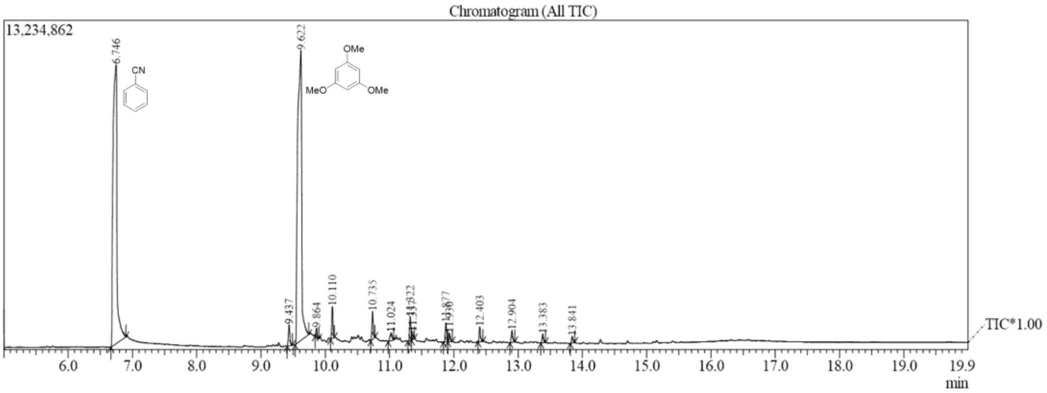

Figure S66. GC-MS spectrum of **1b**, 1,3,5-trimethoxybenzene (16.8 mg, 0.1 mmol) as internal standard.

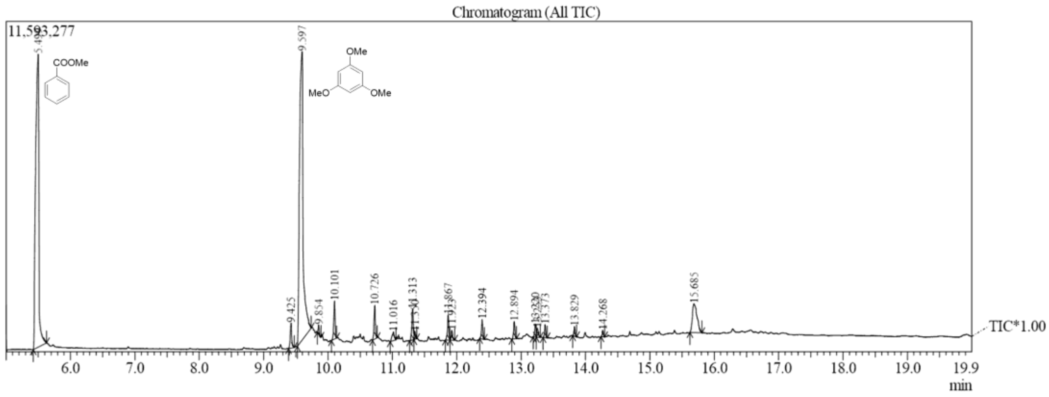

Figure S67. GC-MS spectrum of **1c**, 1,3,5-trimethoxybenzene (16.8 mg, 0.1 mmol) as internal standard.

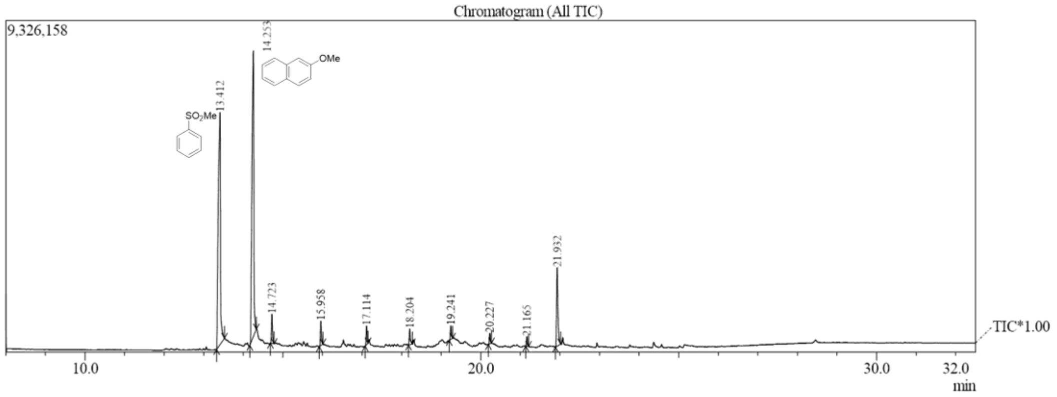

Figure S68. GC-MS spectrum of **1d**, 2-methoxynaphthalene (15.8 mg, 0.1 mmol) as internal standard.

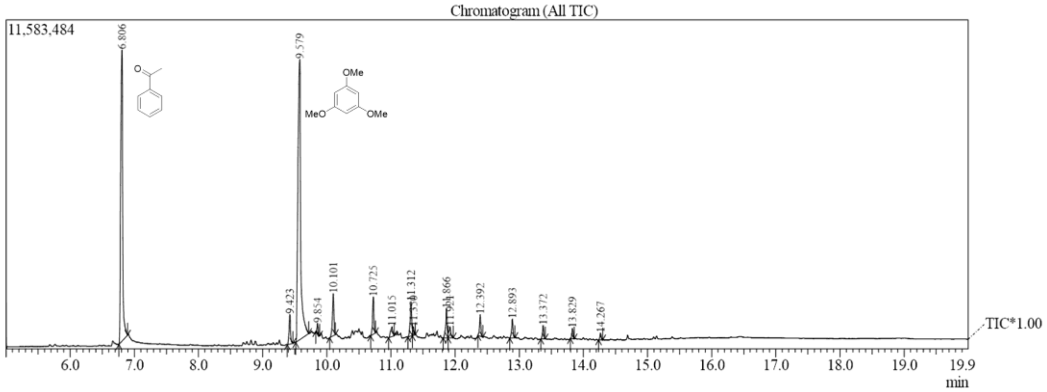

Figure S69. GC-MS spectrum of **1e**, 1,3,5-trimethoxybenzene (16.8 mg, 0.1 mmol) as internal standard.

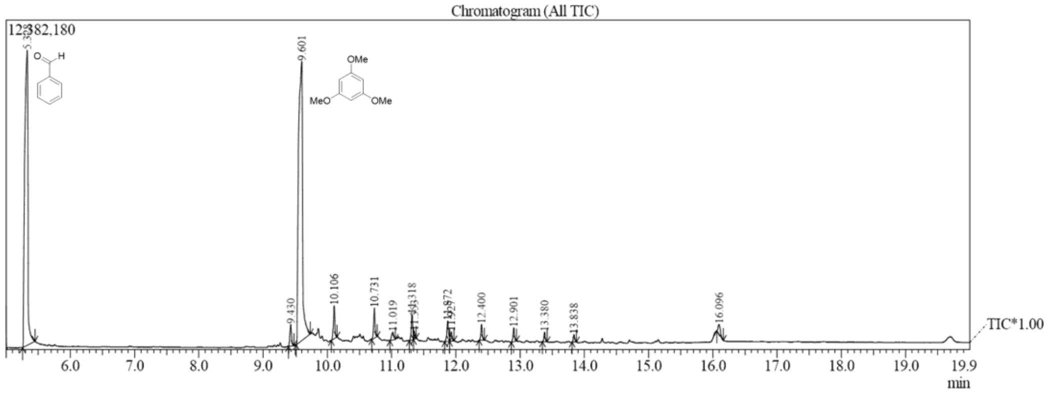

Figure S70. GC-MS spectrum of **1f**, 1,3,5-trimethoxybenzene (16.8 mg, 0.1 mmol) as internal standard.

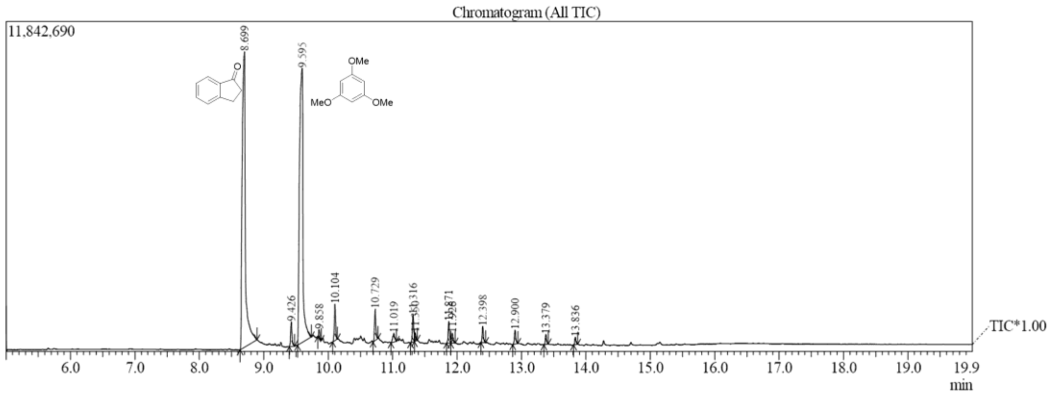

Figure S71. GC-MS spectrum of **1g**, 1,3,5-Trimethoxybenzene (16.8 mg, 0.1 mmol) as internal standard.

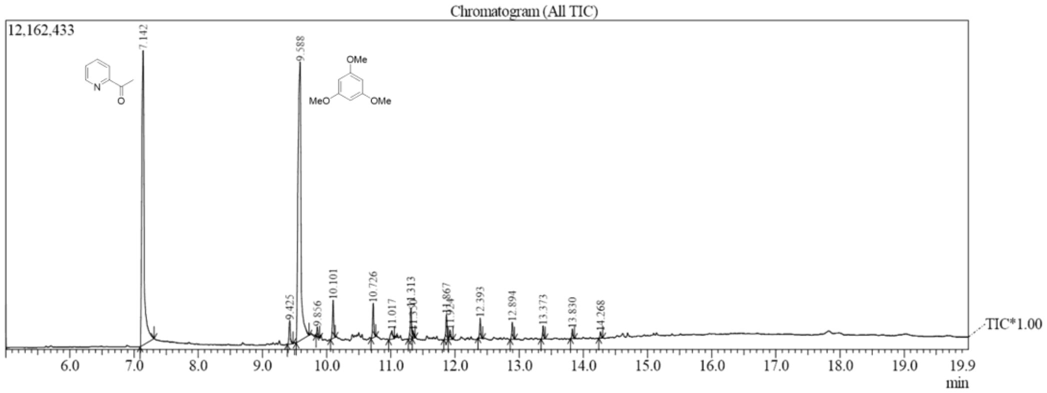

Figure S72. GC-MS spectrum of **1h**, 1,3,5-Trimethoxybenzene (16.8 mg, 0.1 mmol) as internal standard.

6. NMR Spectra

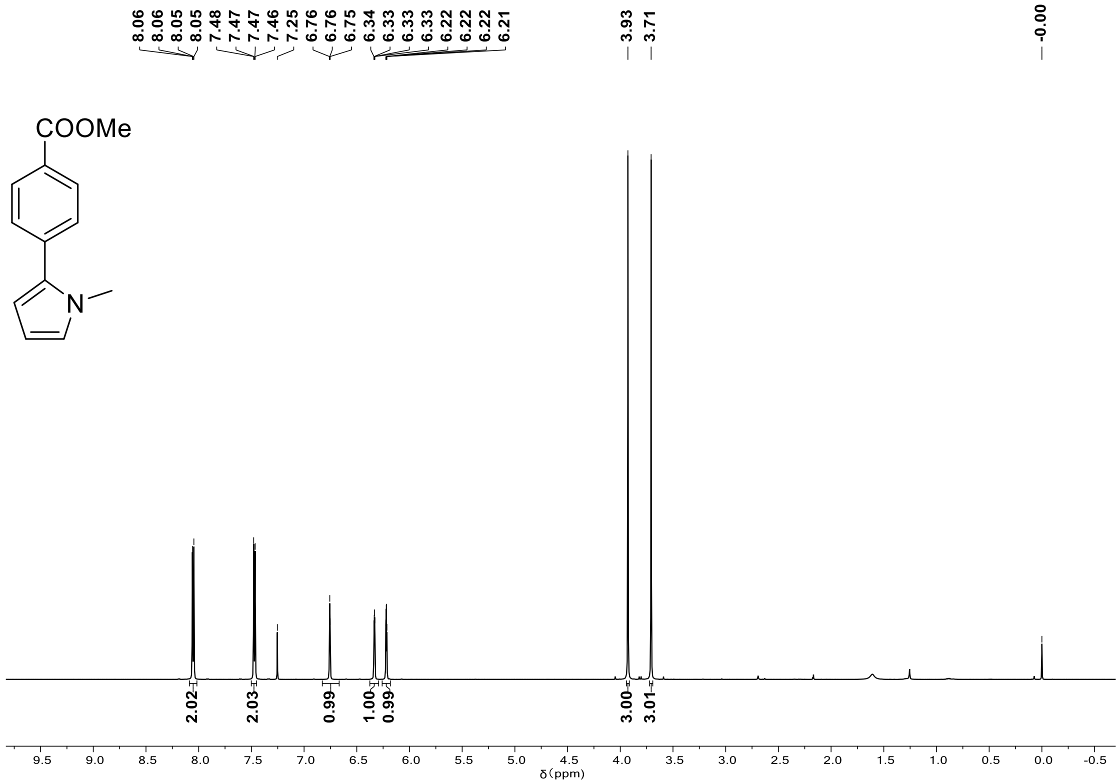

Figure S73. ^{1}H NMR spectrum of **2a** (600 MHz) in $CDCl_3$.

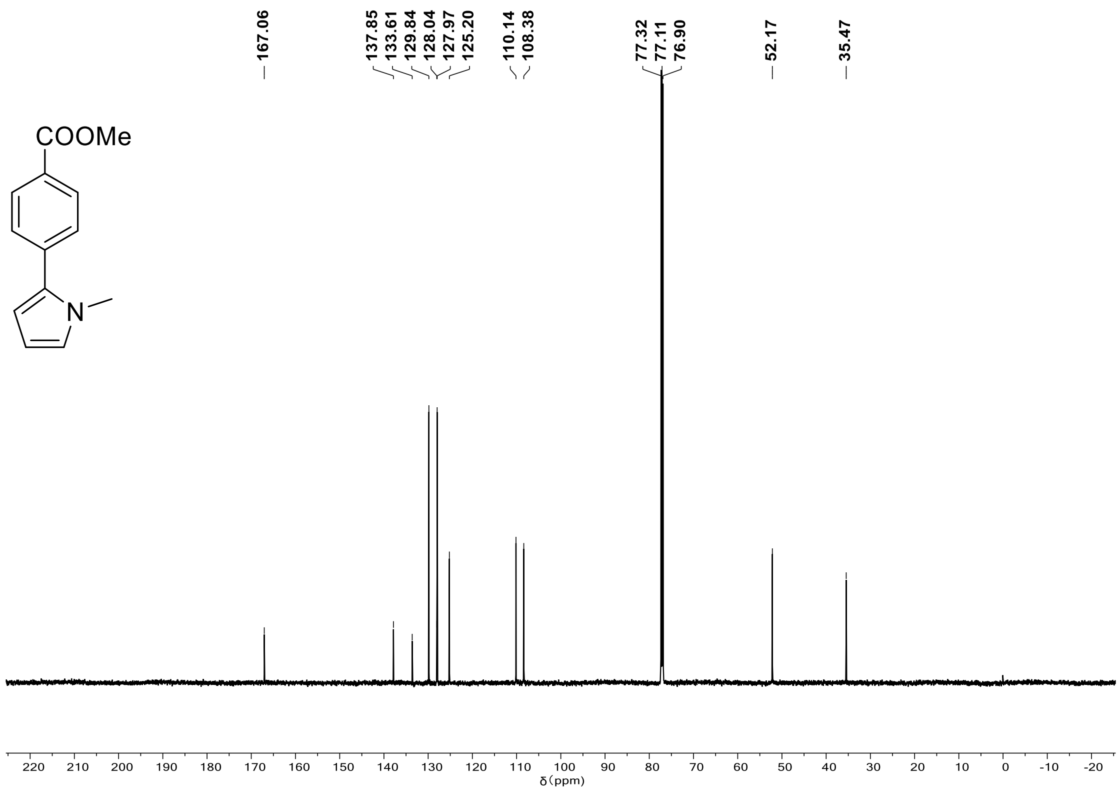

Figure S74. ^{13}C NMR spectrum of **2a** (151 MHz) in $CDCl_3$.

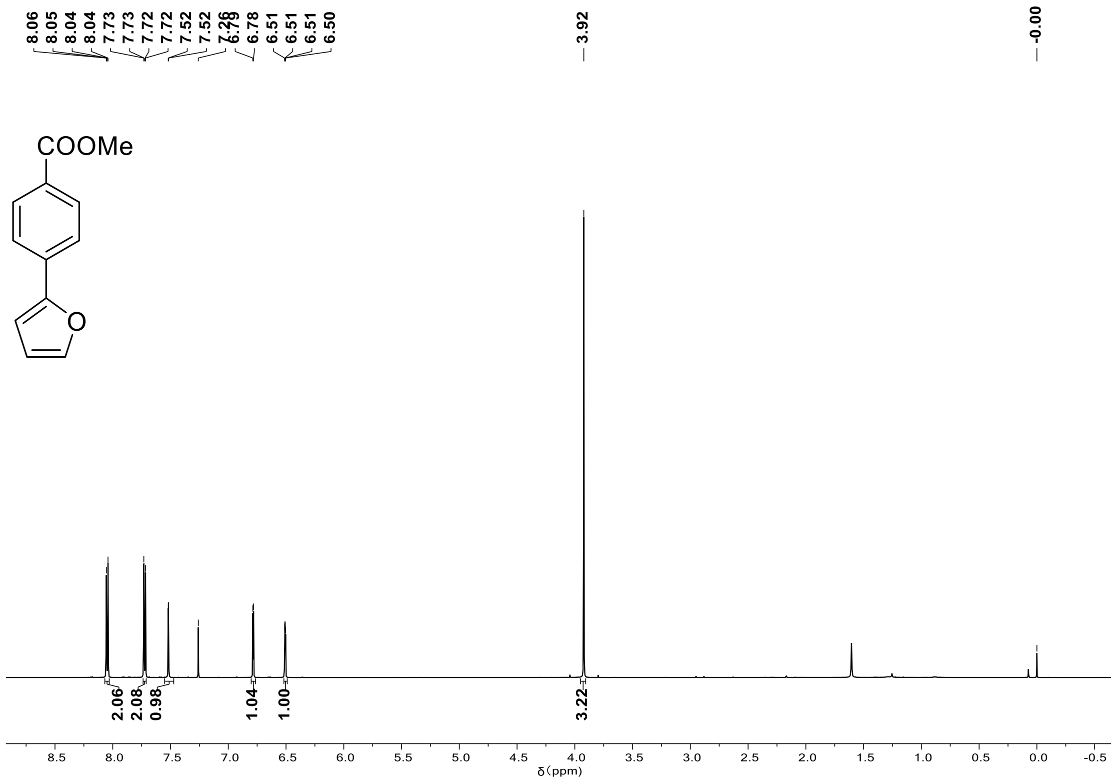

Figure S75. ^{1}H NMR spectrum of **2b** (600 MHz) in $CDCl_3$.

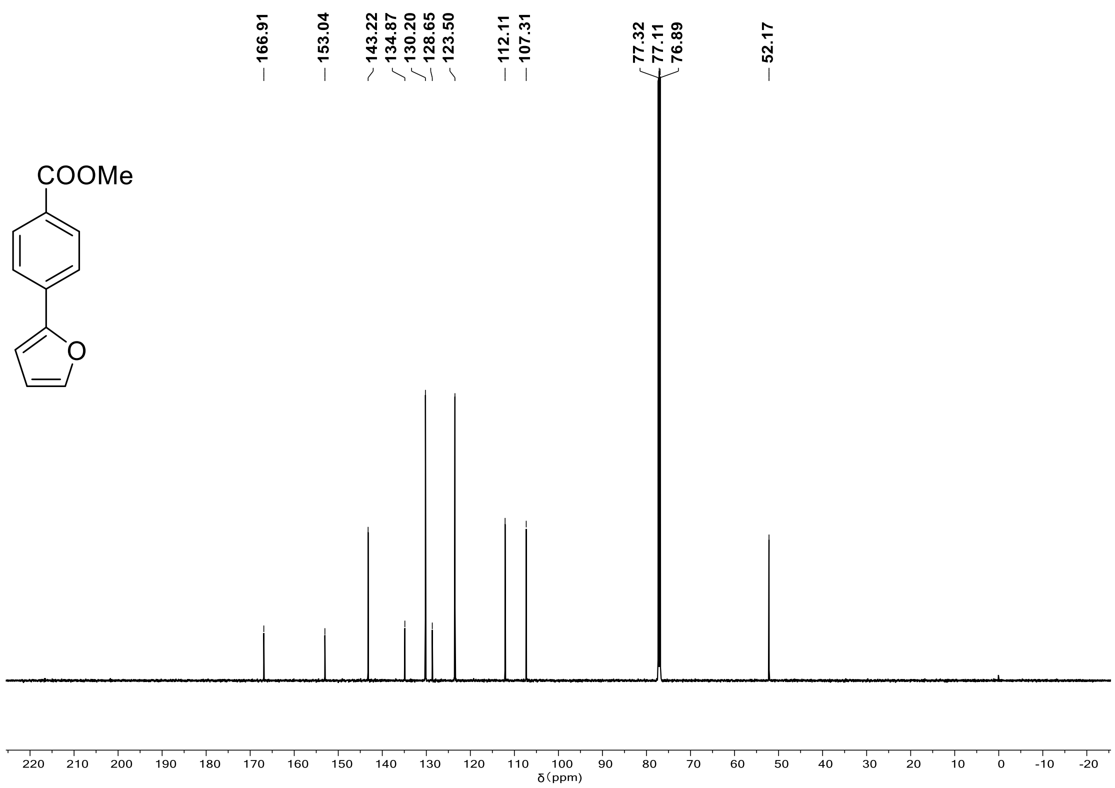

Figure S76. ^{13}C NMR spectrum of **2b** (151 MHz) in $CDCl_3$.

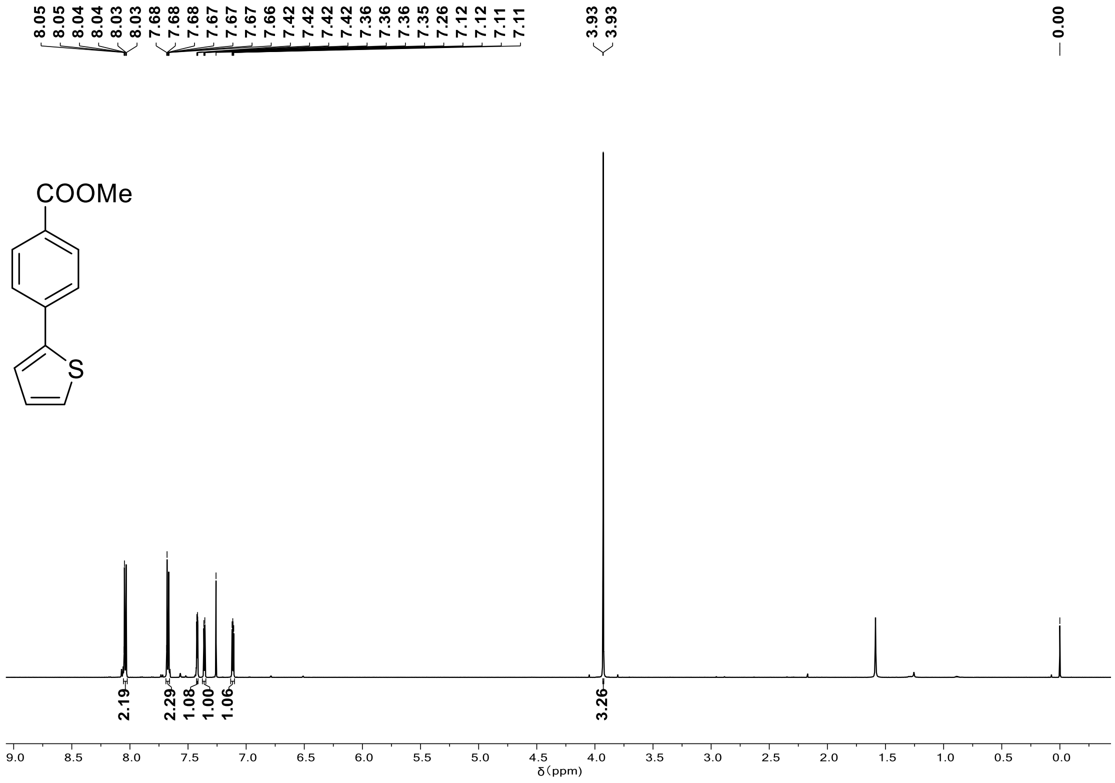

Figure S77. ^{1}H NMR spectrum of **2c** (600 MHz) in $CDCl_3$.

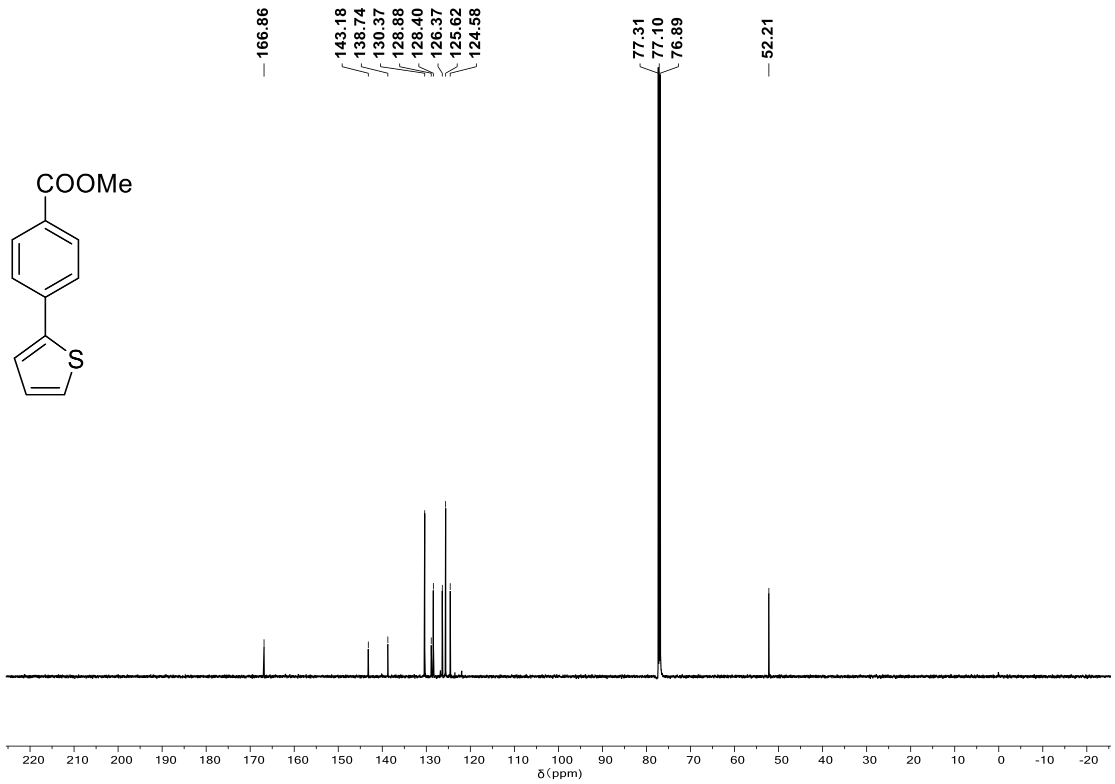

Figure S78. ^{13}C NMR spectrum of **2c** (151 MHz) in $CDCl_3$.

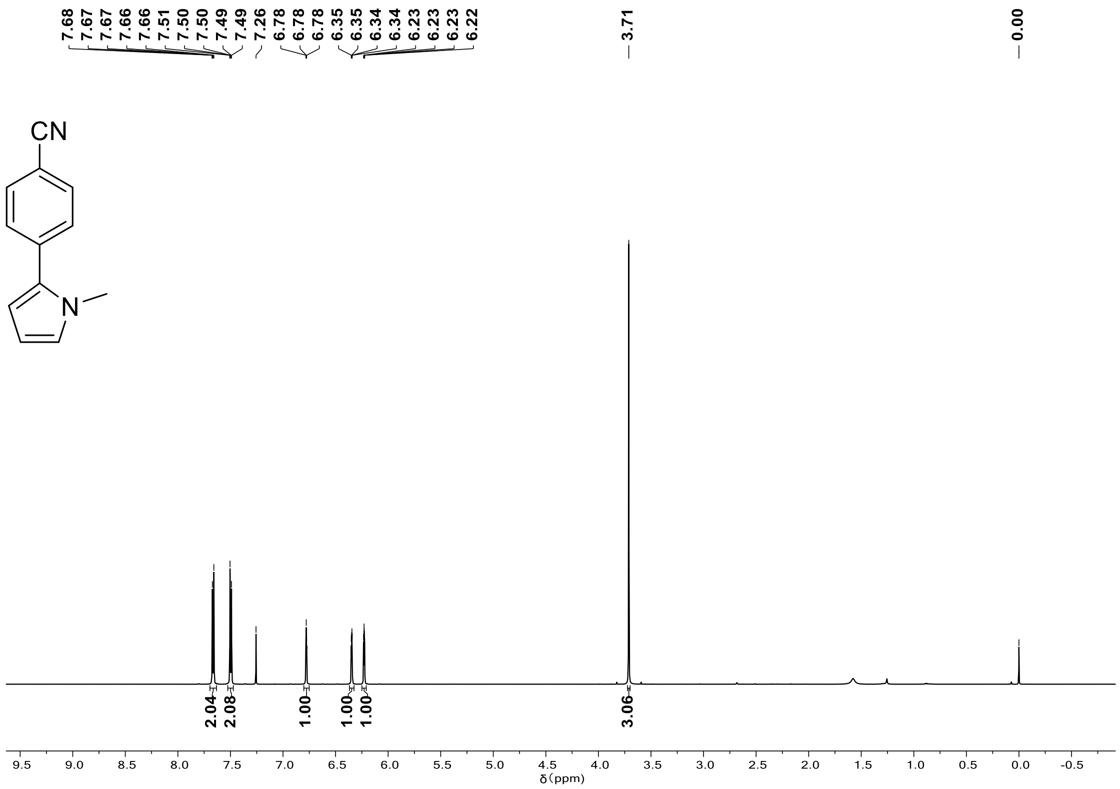

Figure S79. ^{1}H NMR spectrum of **2d** (600 MHz) in $CDCl_3$.

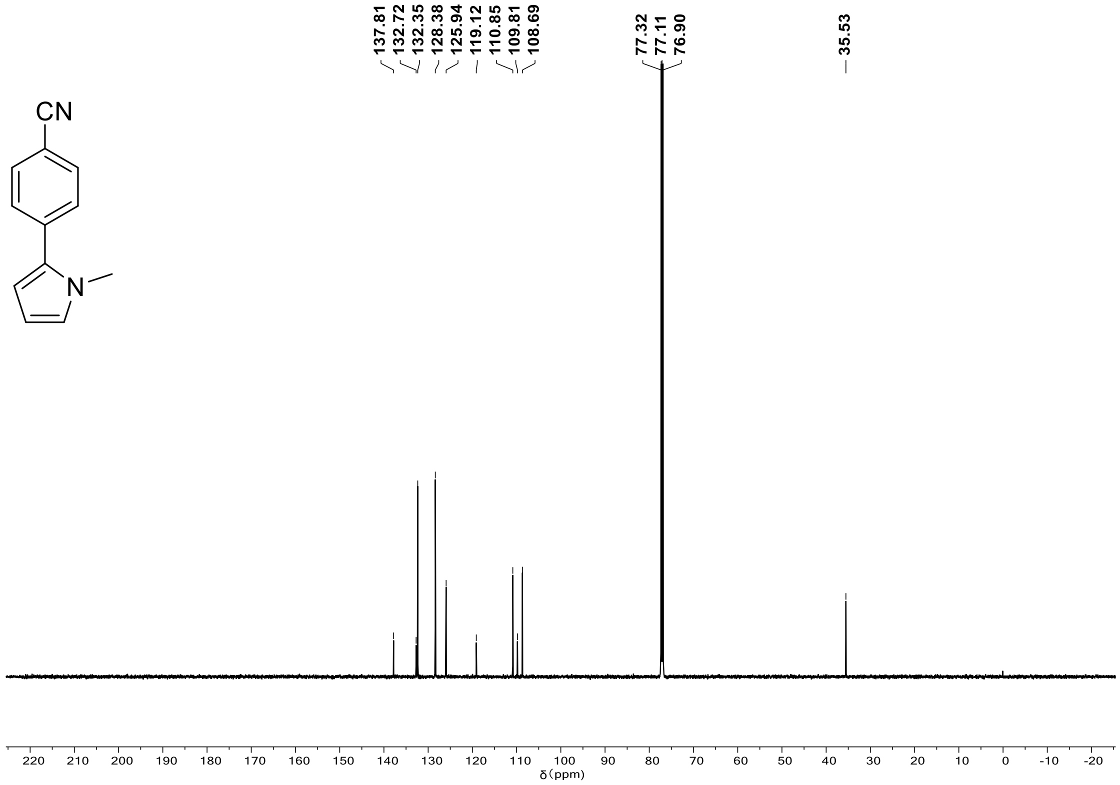

Figure S80. ^{13}C NMR spectrum of **2d** (151 MHz) in $CDCl_3$.

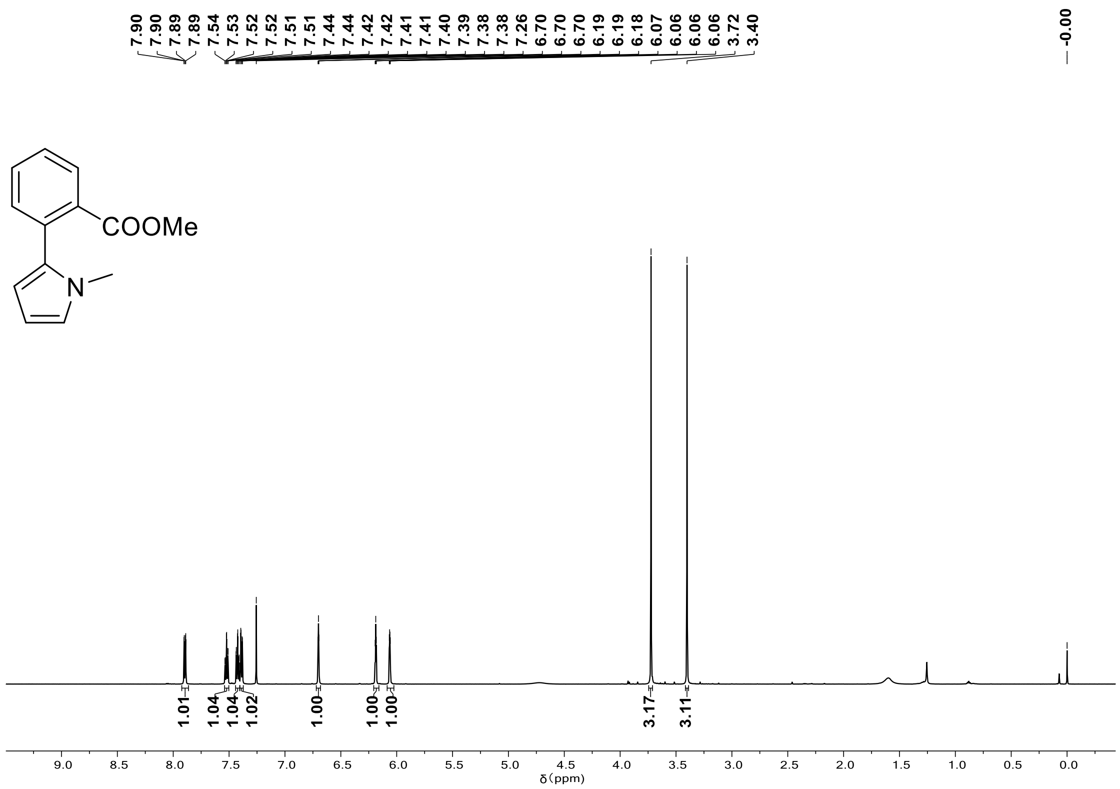

Figure S81. ^{1}H NMR spectrum of **2e** (600 MHz) in $CDCl_3$.

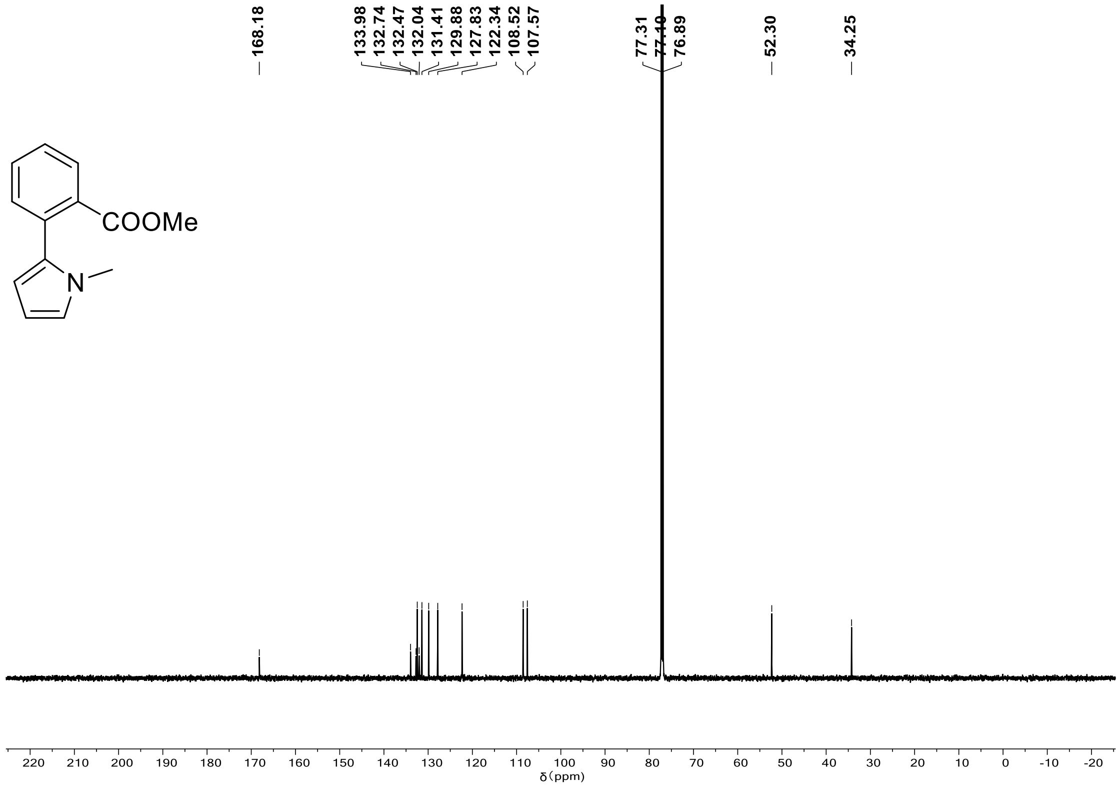

Figure S82. ^{13}C NMR spectrum of **2e** (151 MHz) in $CDCl_3$.

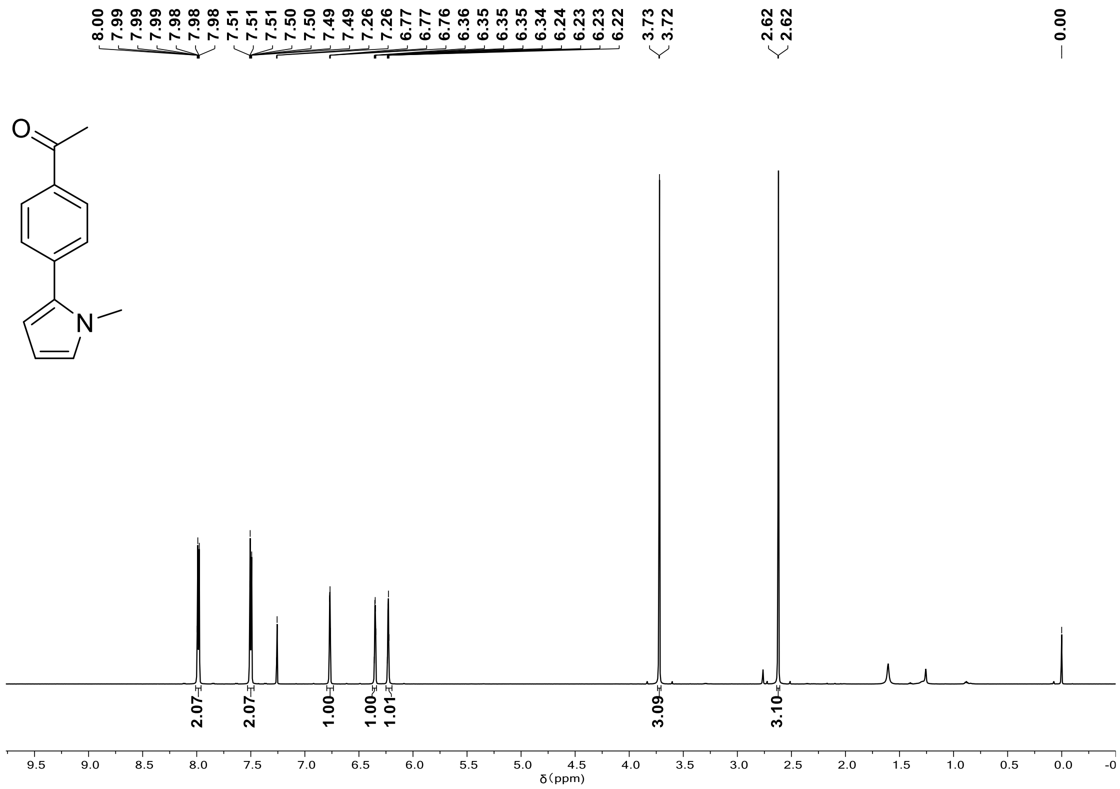

Figure S83. ^{1}H NMR spectrum of **2f** (600 MHz) in $CDCl_3$.

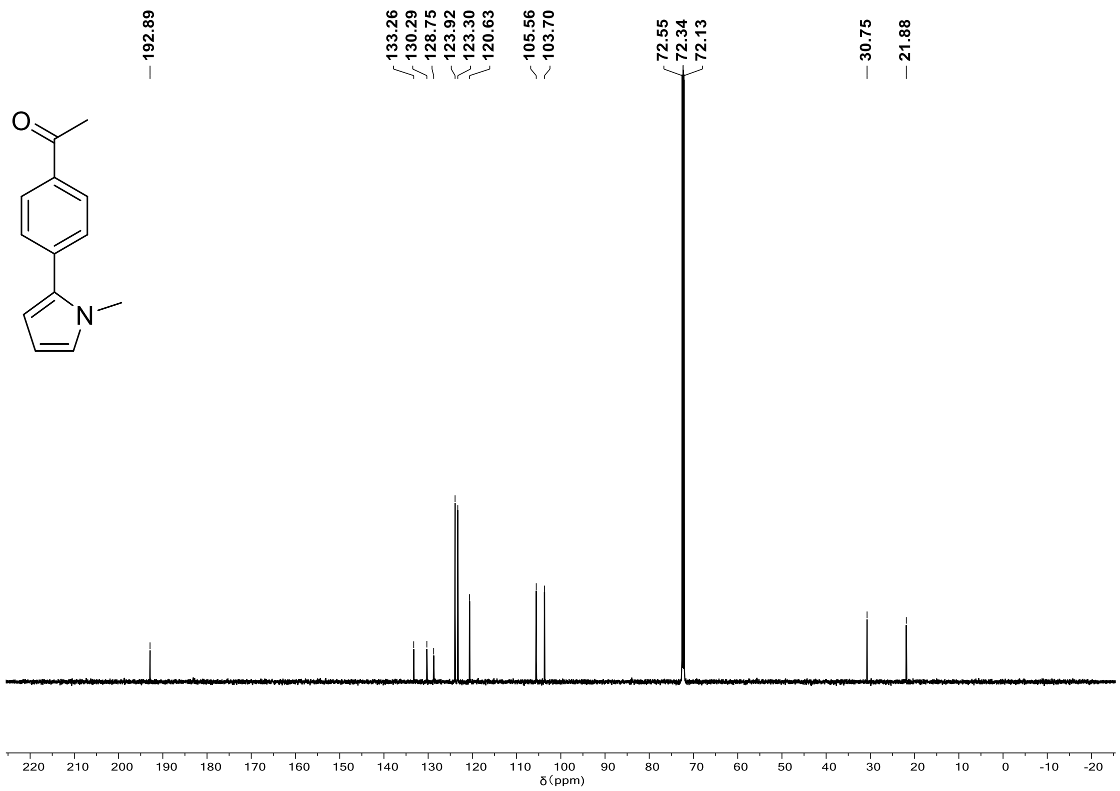

Figure S84. ^{13}C NMR spectrum of **2f** (151 MHz) in $CDCl_3$.

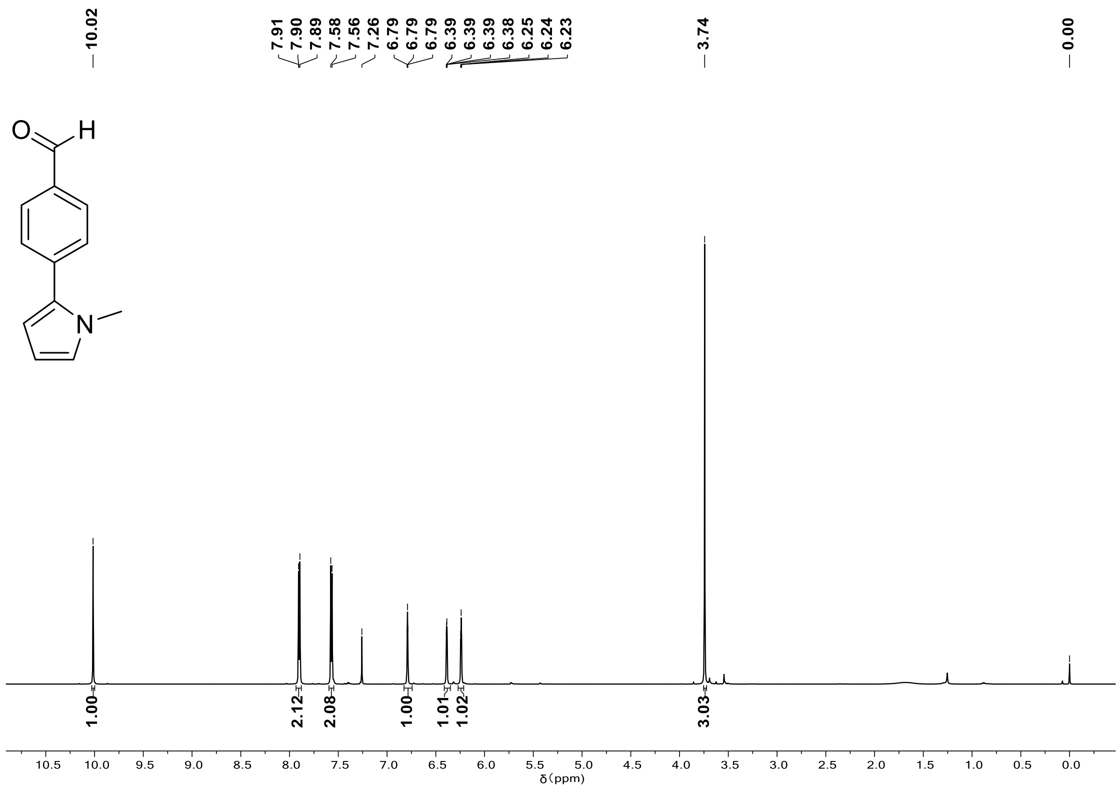

Figure S85. ^{1}H NMR spectrum of **2g** (600 MHz) in $CDCl_3$.

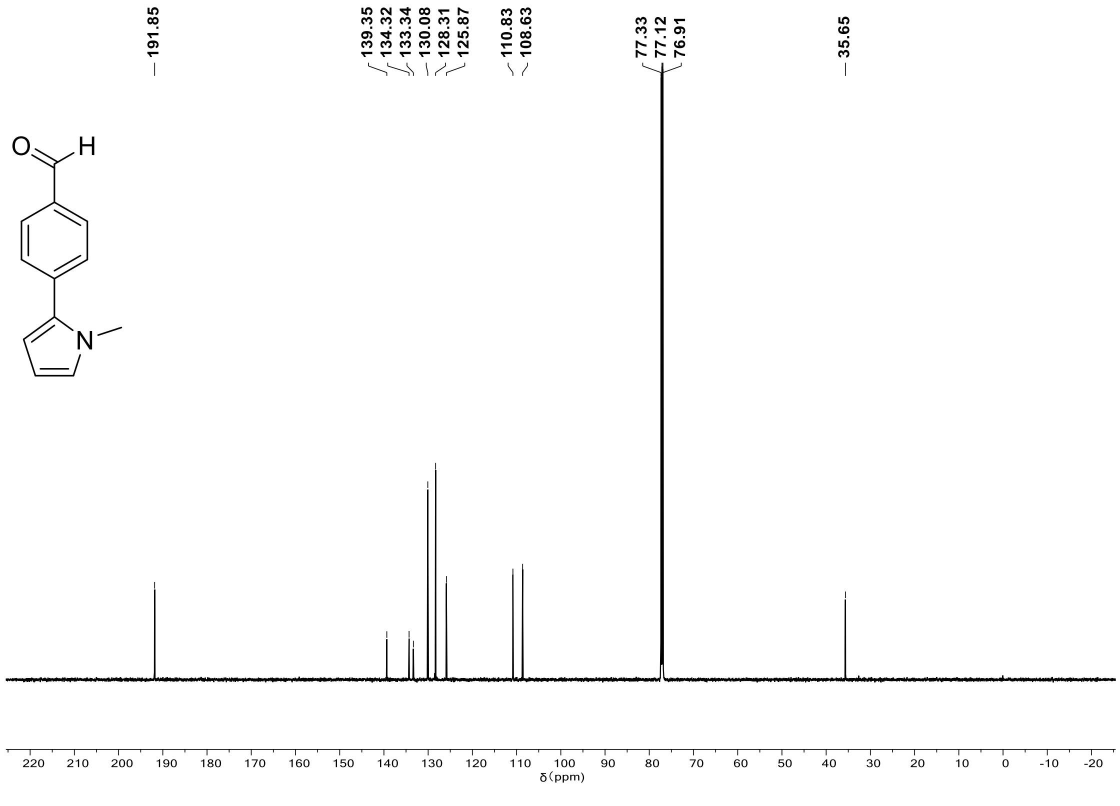

Figure S86. ^{13}C NMR spectrum of **2g** (151 MHz) in $CDCl_3$.

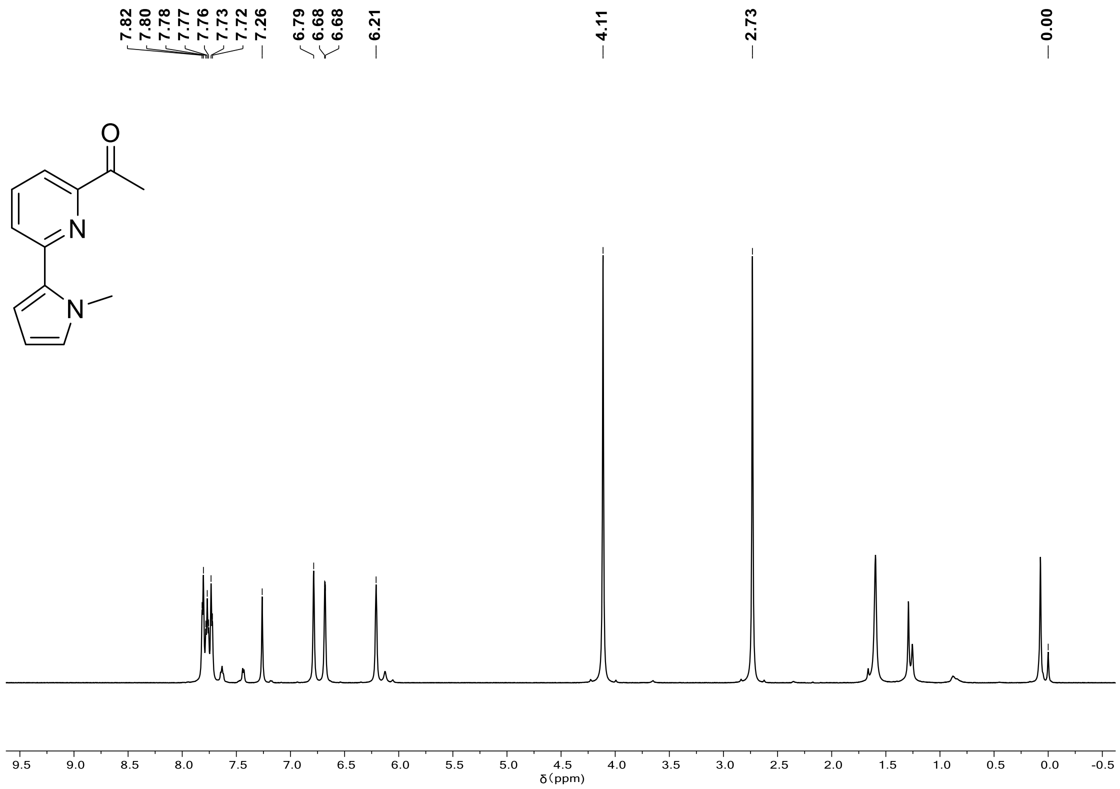

Figure S87. ^{1}H NMR spectrum of **2h** (600 MHz) in $CDCl_3$.

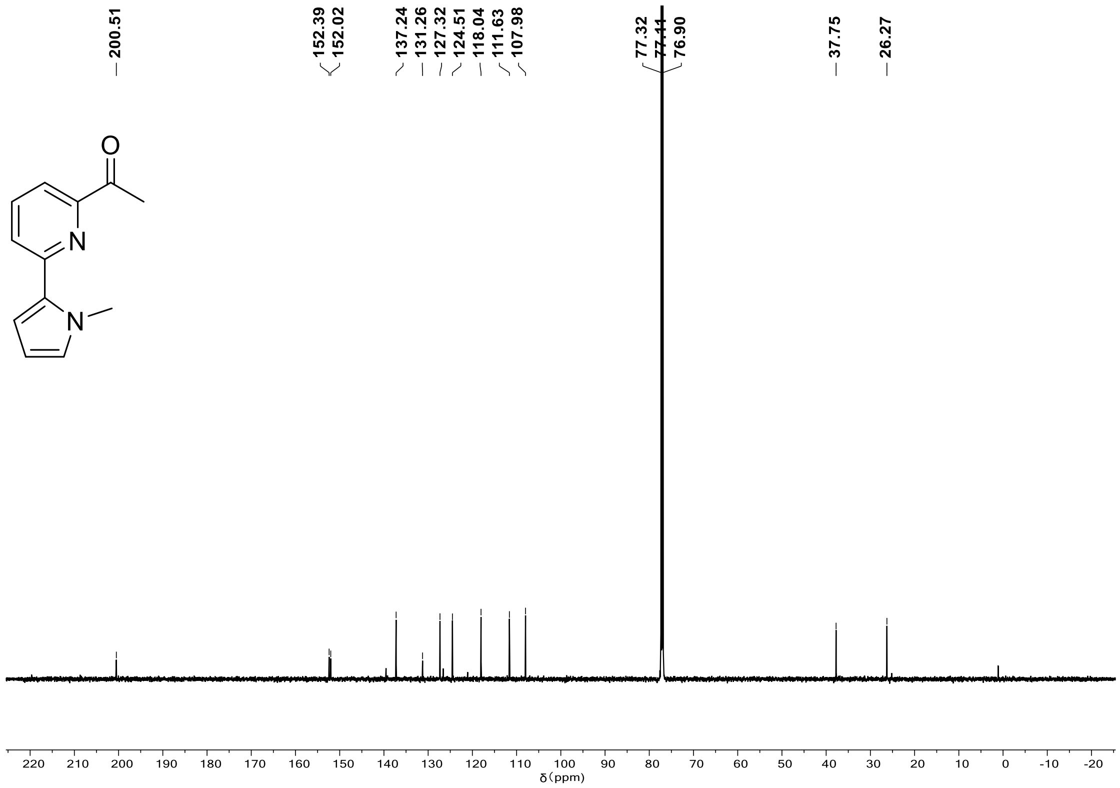

Figure S88. ^{13}C NMR spectrum of **2h** (151 MHz) in $CDCl_3$.

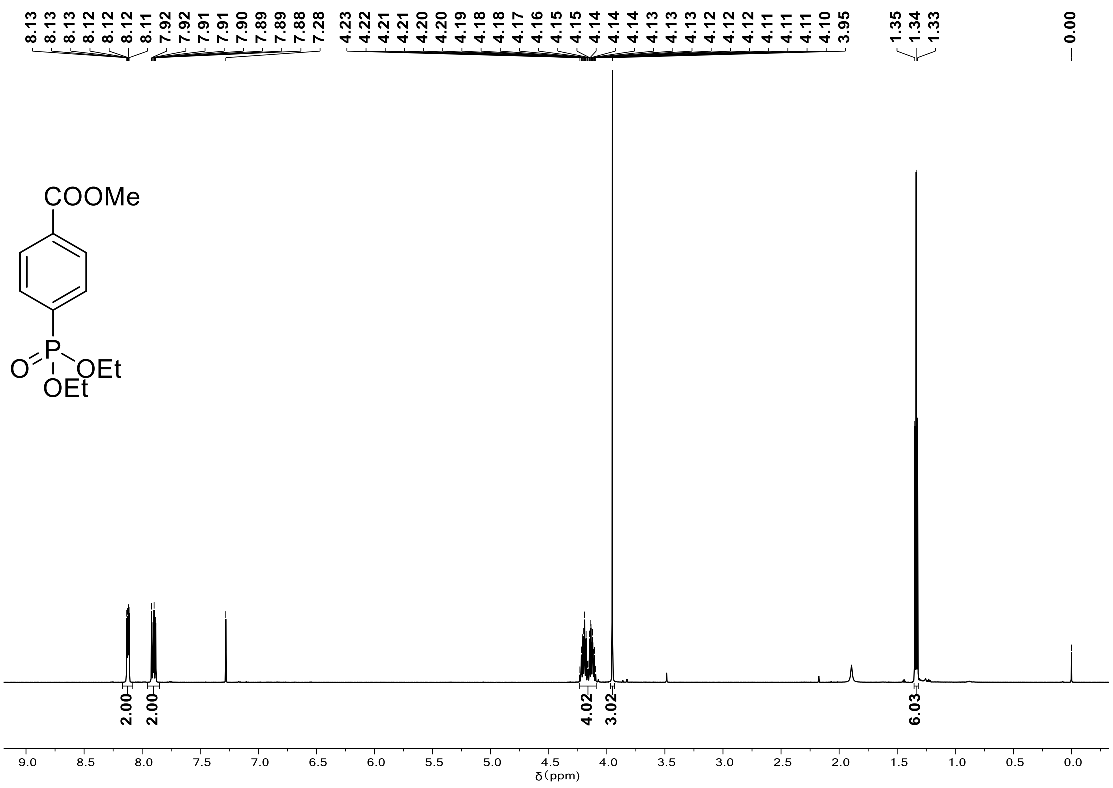

Figure S89. ^{1}H NMR spectrum of **2i** (600 MHz) in $CDCl_3$.

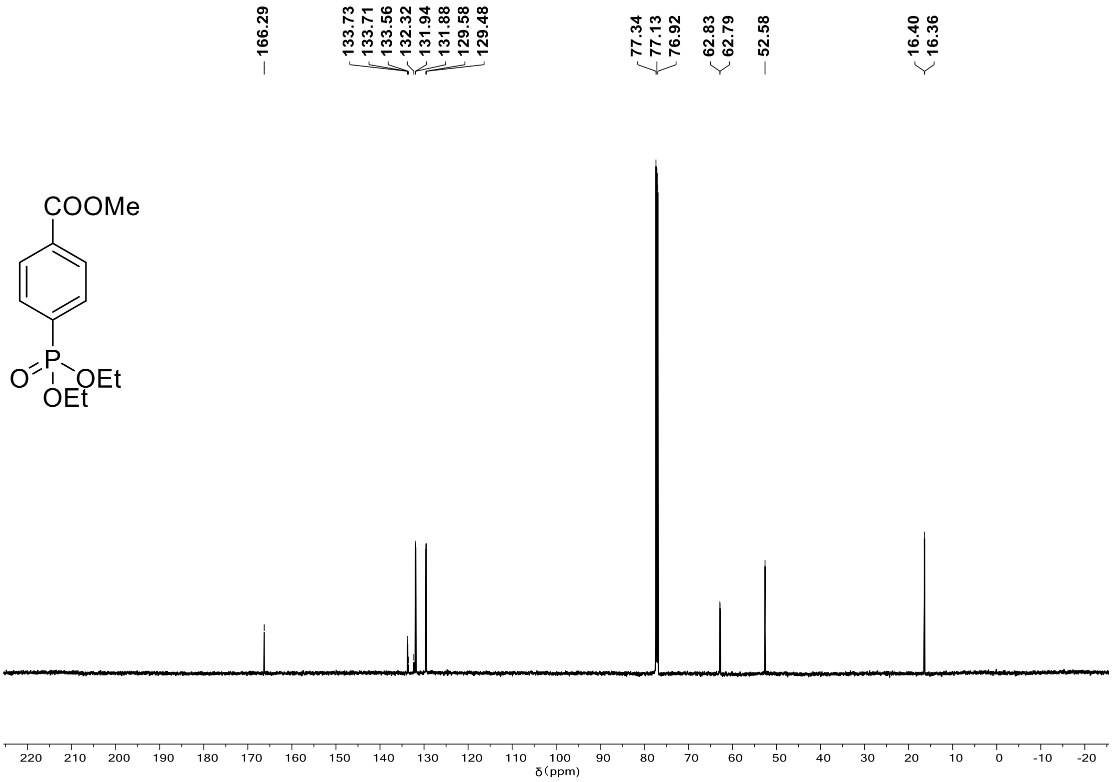

Figure S90. ^{13}C NMR spectrum of **2i** (151 MHz) in $CDCl_3$.

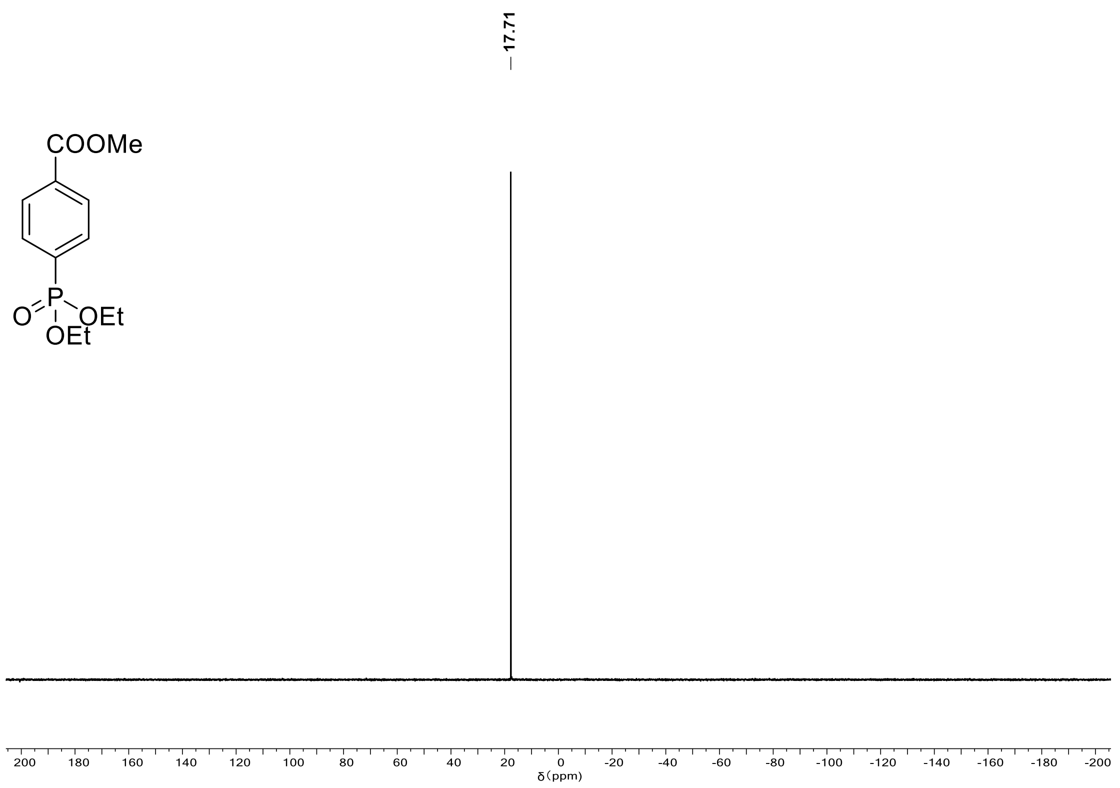

Figure S91. ^{31}P NMR spectrum of **2i** (243 MHz) in $CDCl_3$.

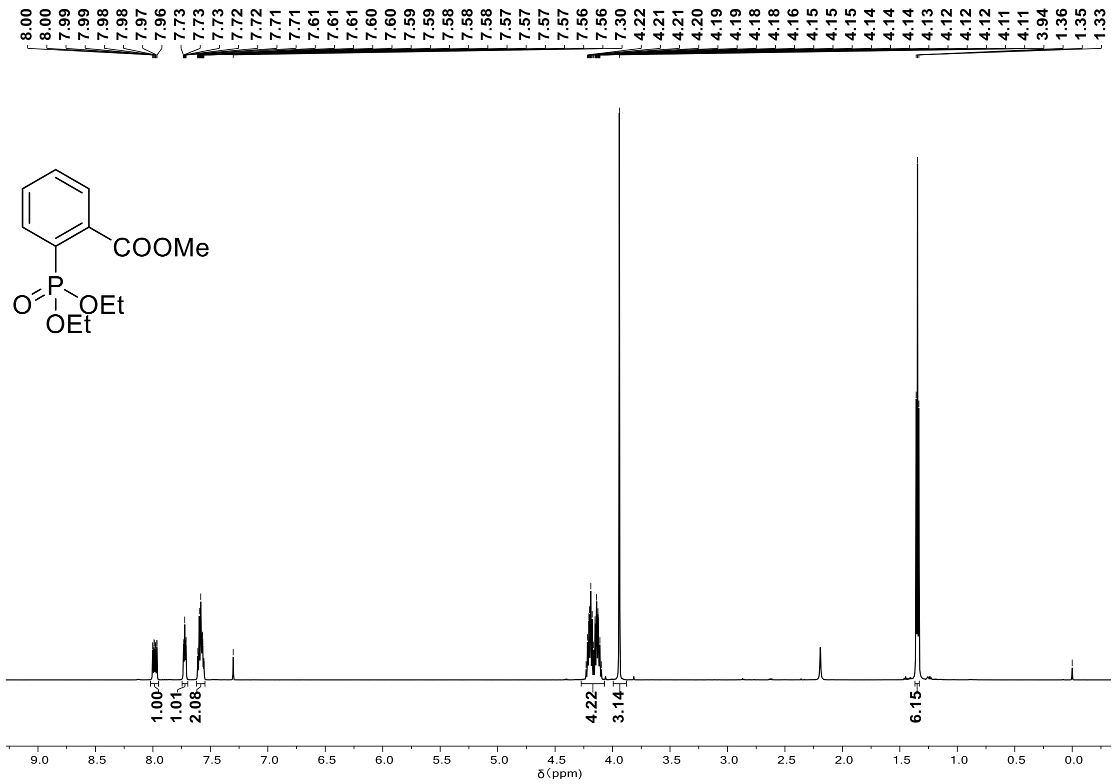

Figure S92. ^{1}H NMR spectrum of **2j** (600 MHz) in $CDCl_3$.

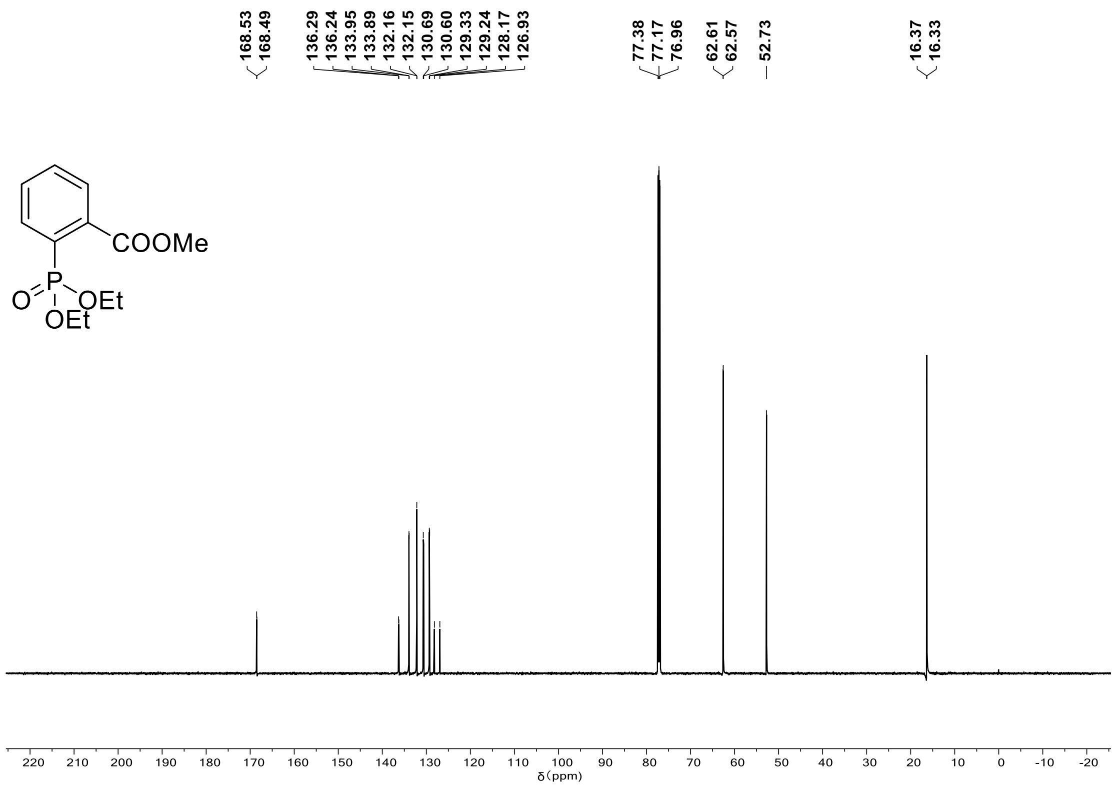

Figure S93. ^{13}C NMR spectrum of **2j** (151 MHz) in $CDCl_3$.

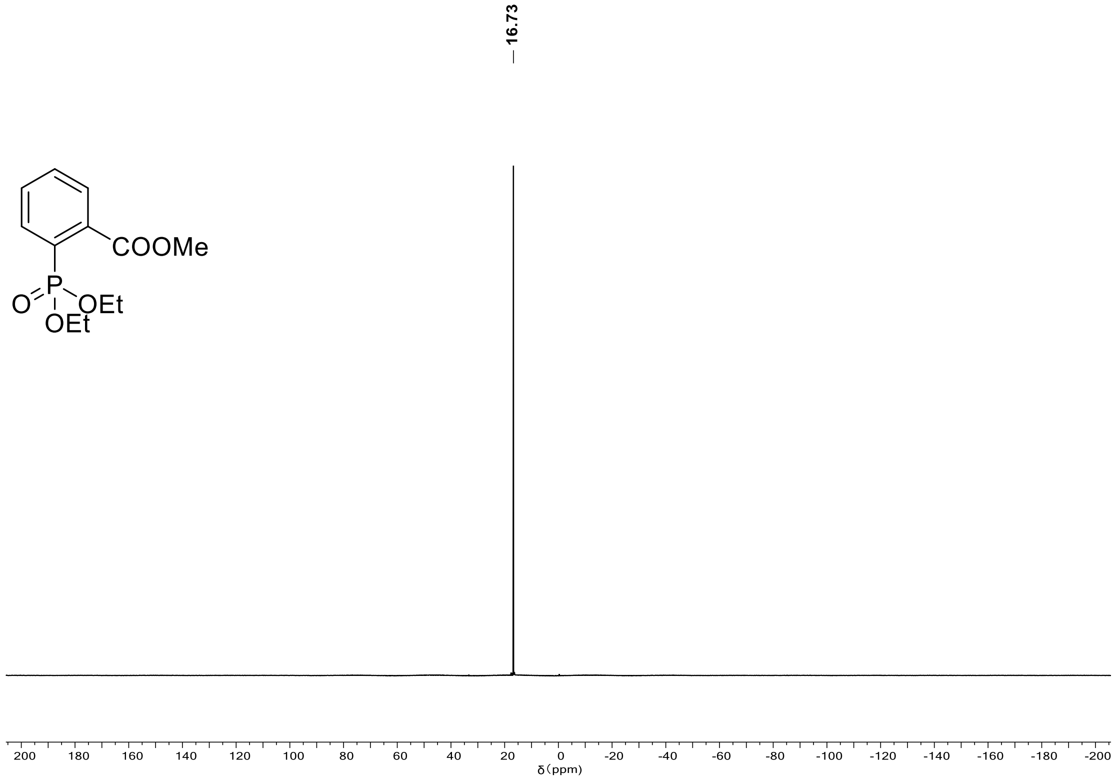

Figure S94. ^{31}P NMR spectrum of **2j** (243 MHz) in $CDCl_3$.

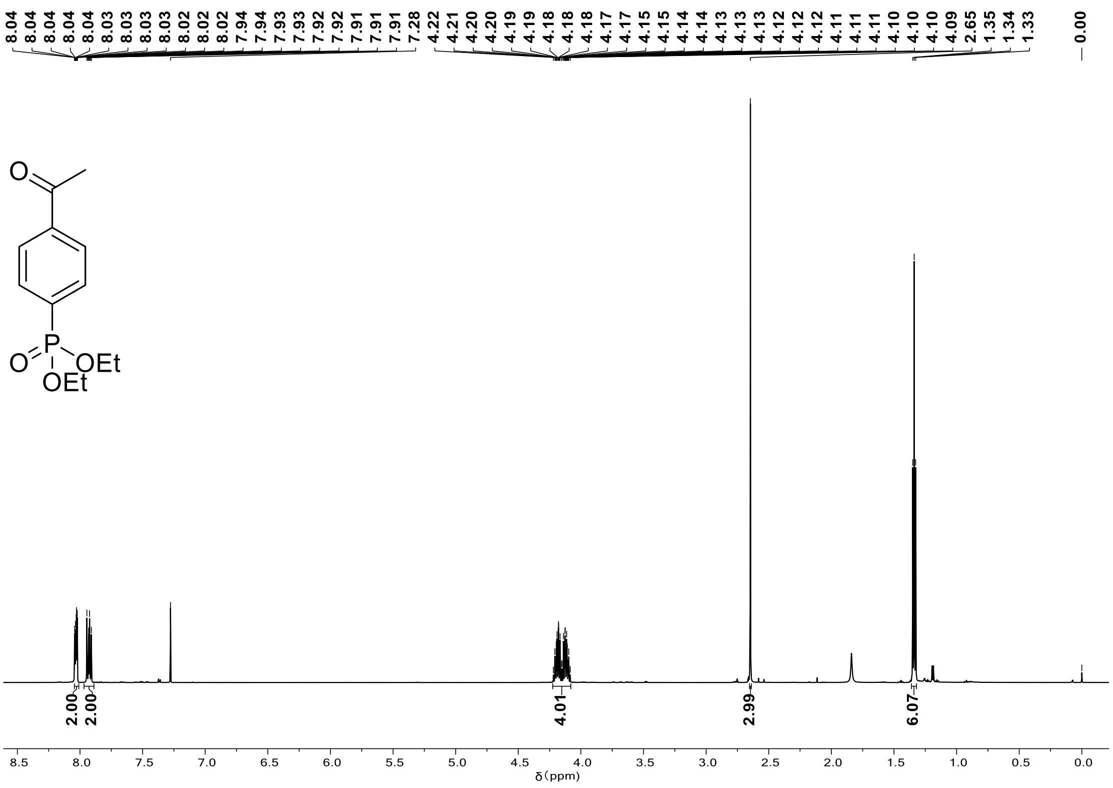

Figure S95. ^{1}H NMR spectrum of **2k** (600 MHz) in $CDCl_3$.

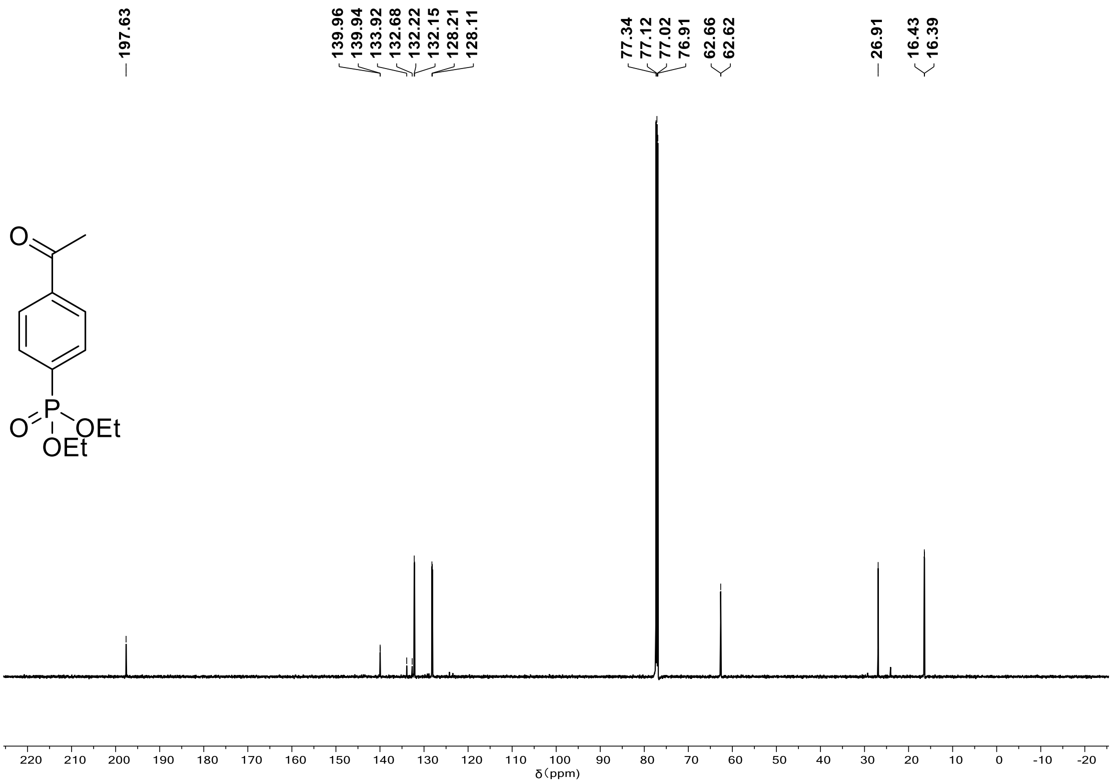

Figure S96. ^{13}C NMR spectrum of **2k** (151 MHz) in $CDCl_3$.

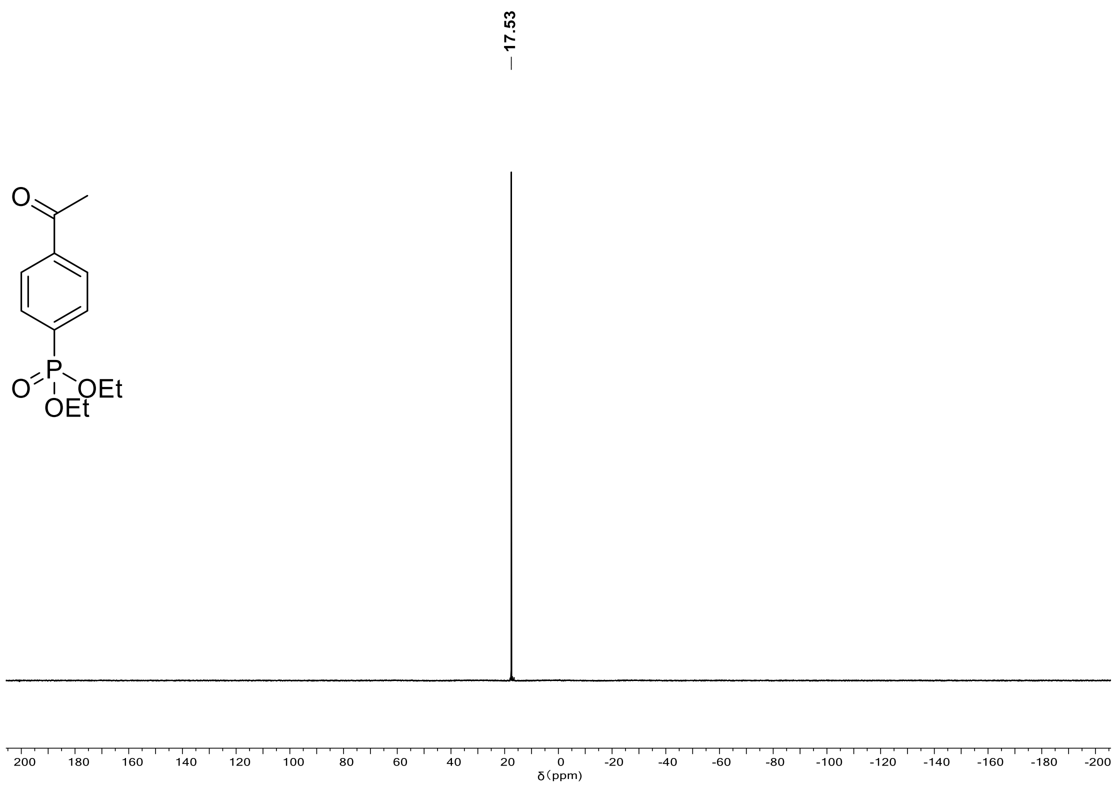

Figure S97. ^{31}P NMR spectrum of **2k** (243 MHz) in $CDCl_3$.

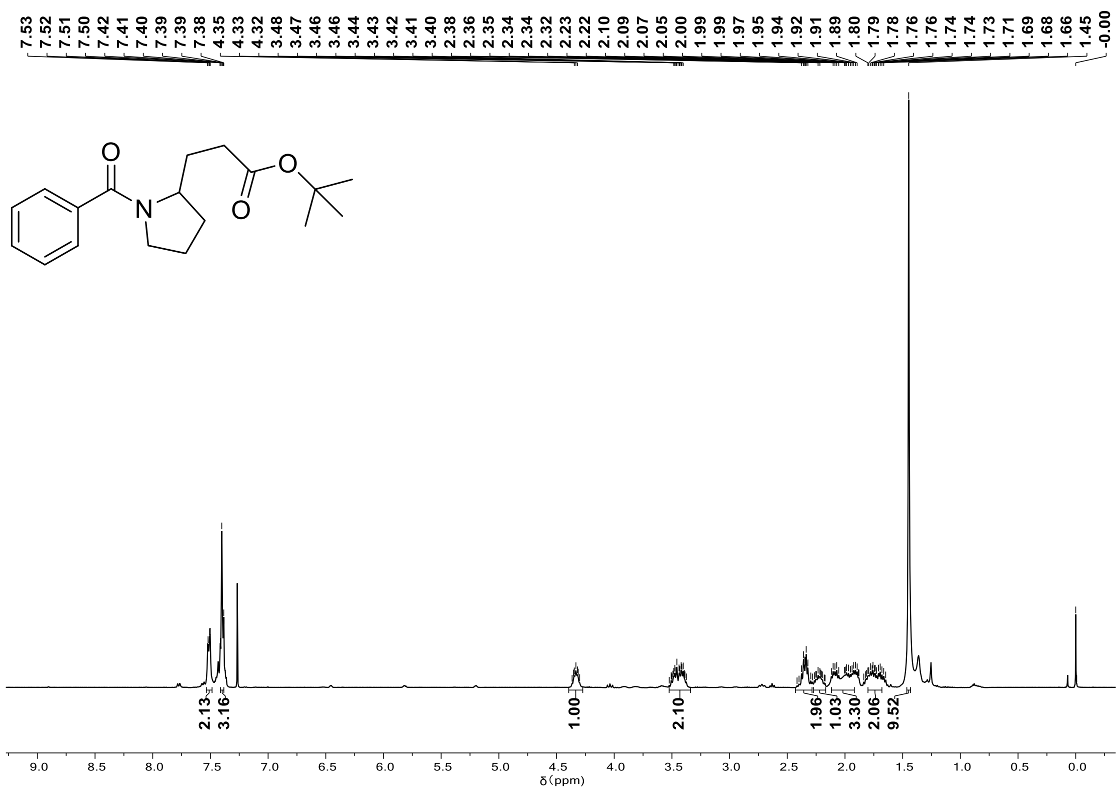

Figure S98. ^{1}H NMR spectrum of **3a** (600 MHz) in $CDCl_3$.

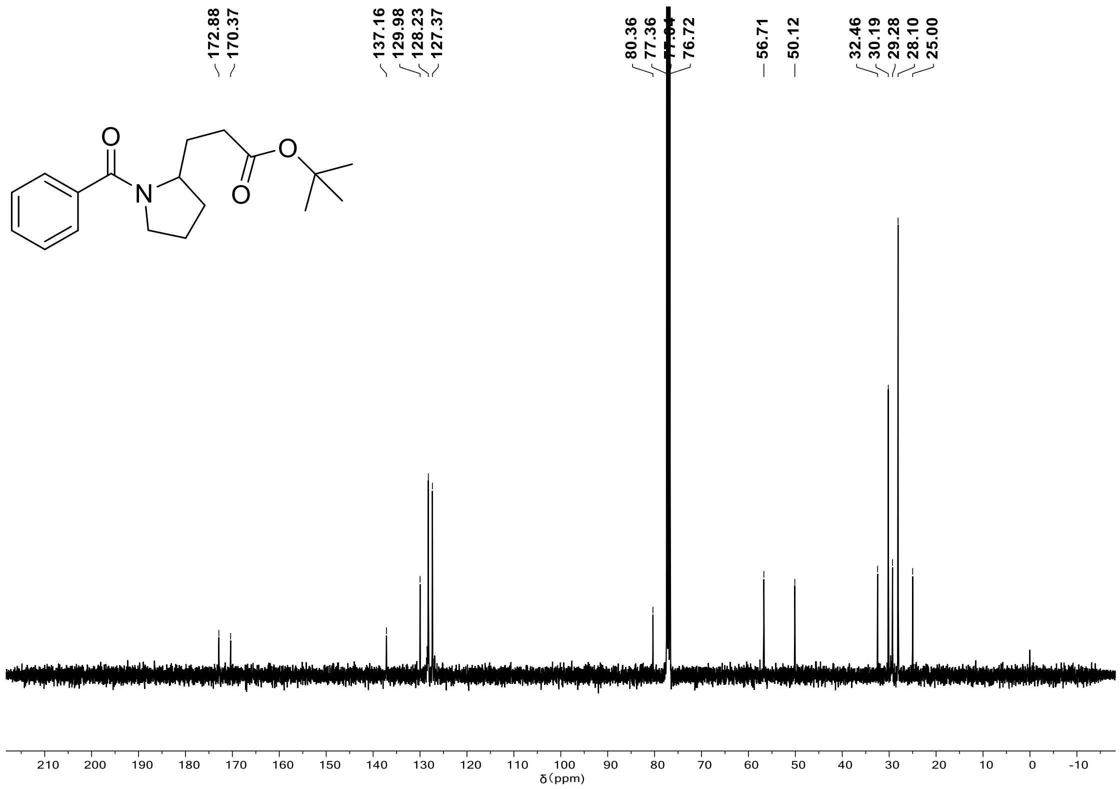

Figure S99. ^{13}C NMR spectrum of **3a** (151 MHz) in $CDCl_3$.

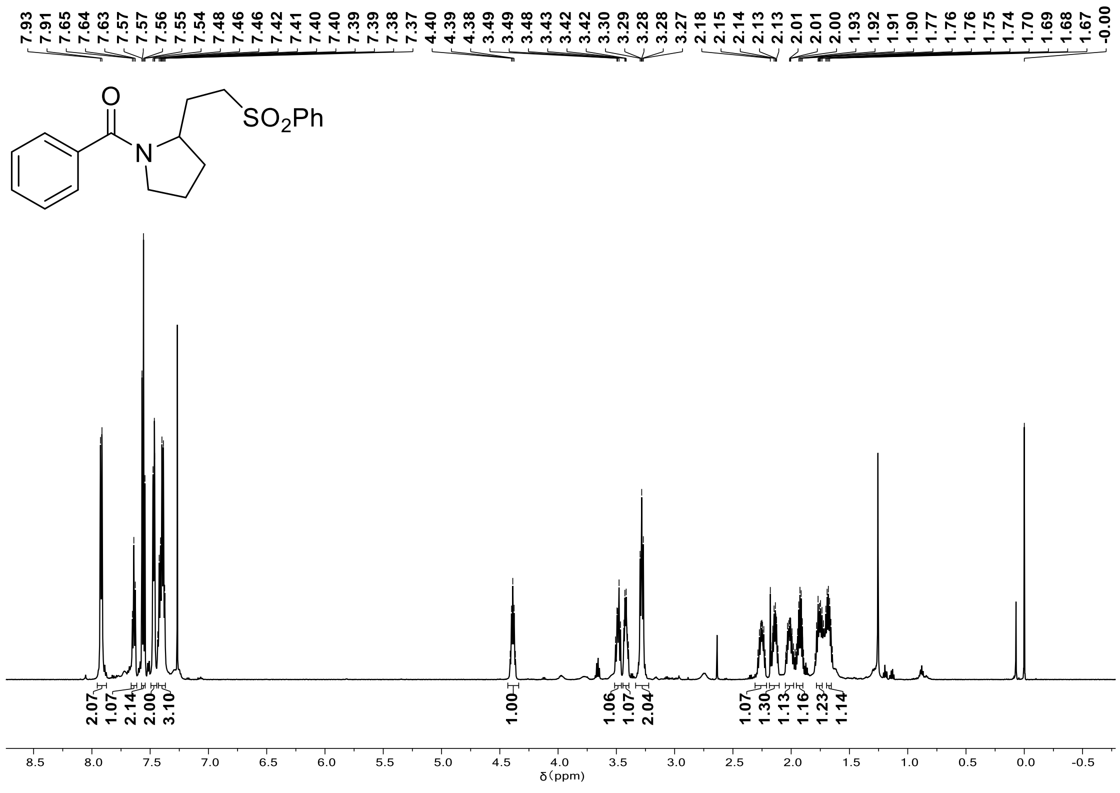

Figure S100. ^{1}H NMR spectrum of **3b** (600 MHz) in $CDCl_3$.

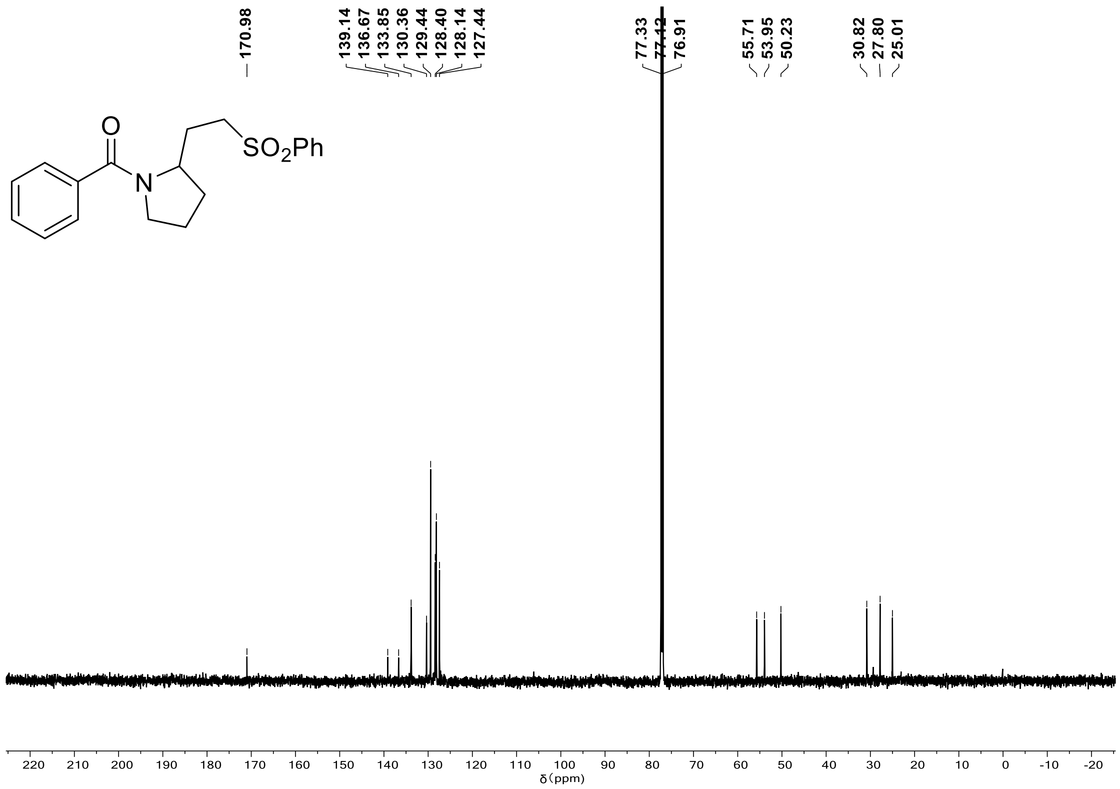

Figure S101. ^{13}C NMR spectrum of **3b** (151 MHz) in $CDCl_3$.

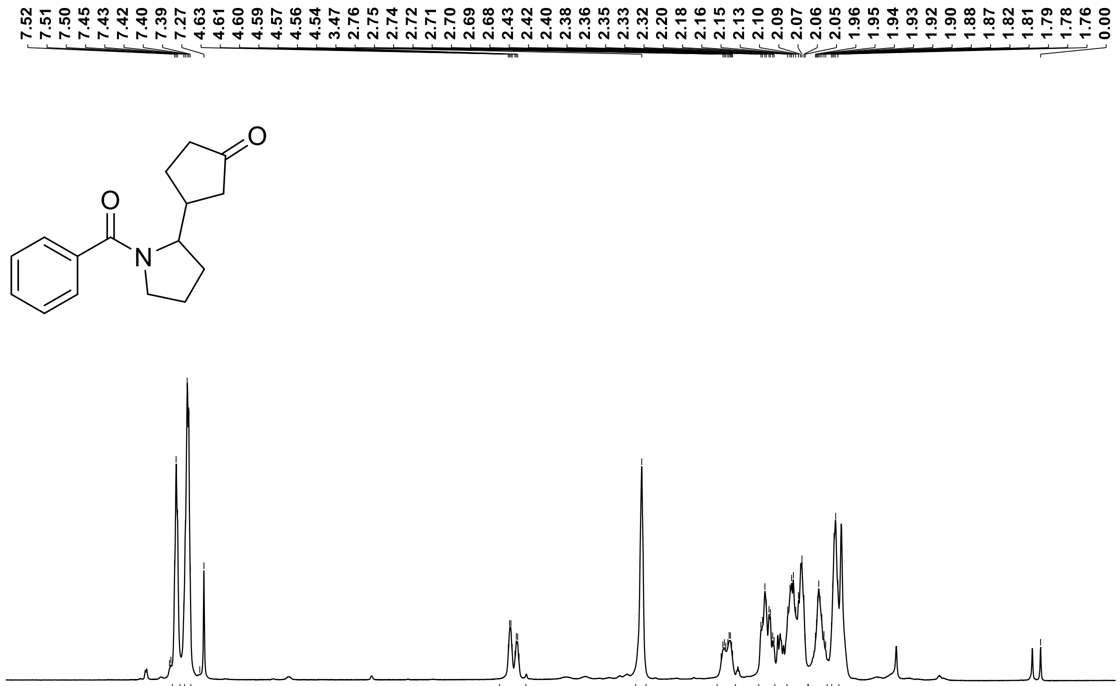

Figure S102. ^{1}H NMR spectrum of **3c** (600 MHz) in $CDCl_3$.

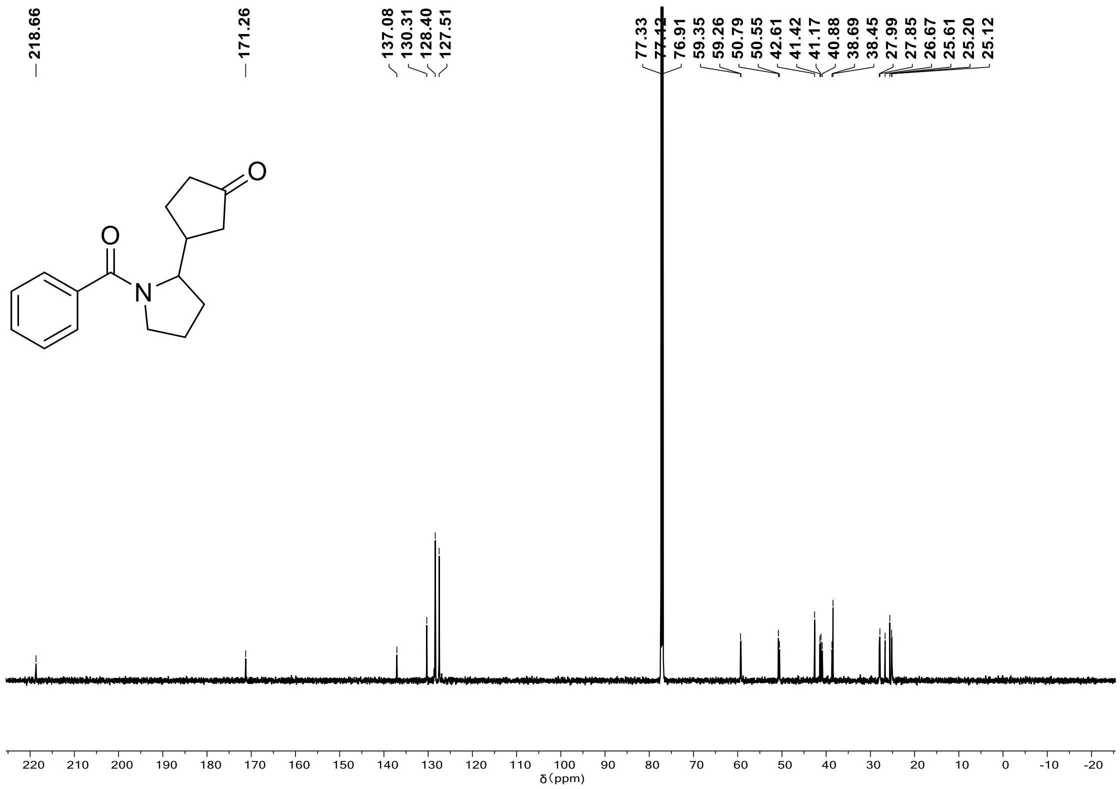

Figure S103. ^{13}C NMR spectrum of **3c** (151 MHz) in $CDCl_3$.

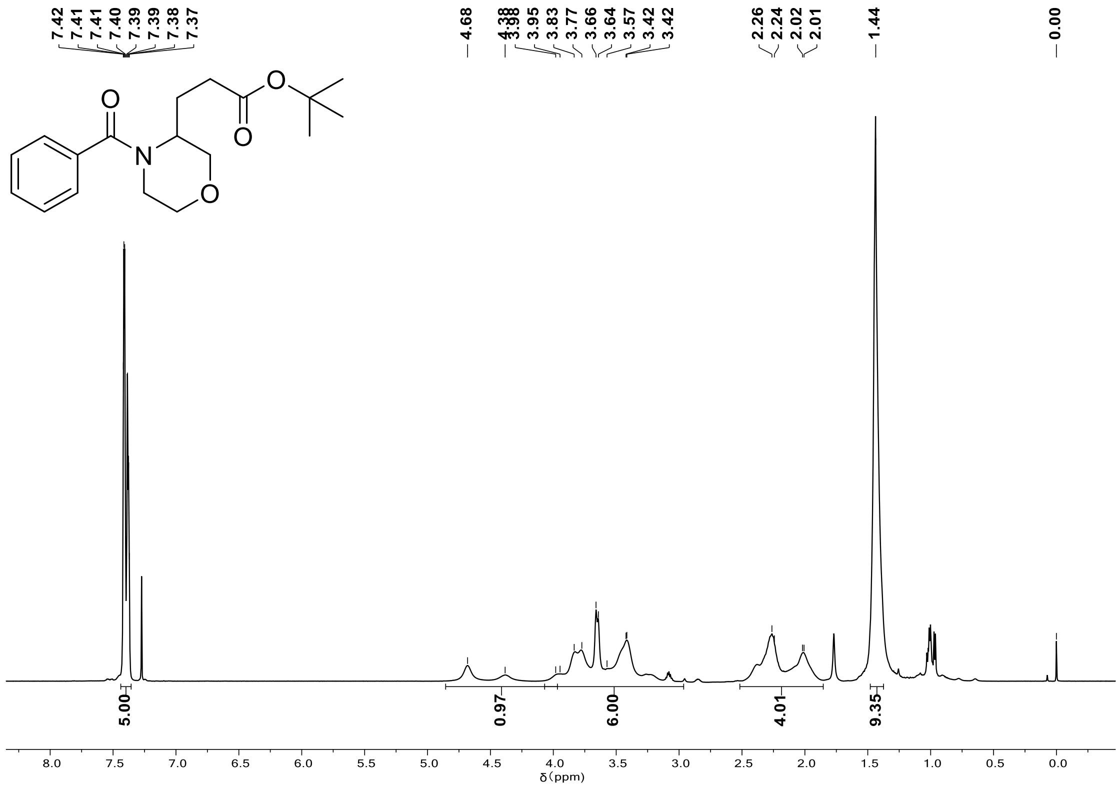

Figure S104. ^{1}H NMR spectrum of **3d** (600 MHz) in $CDCl_3$.

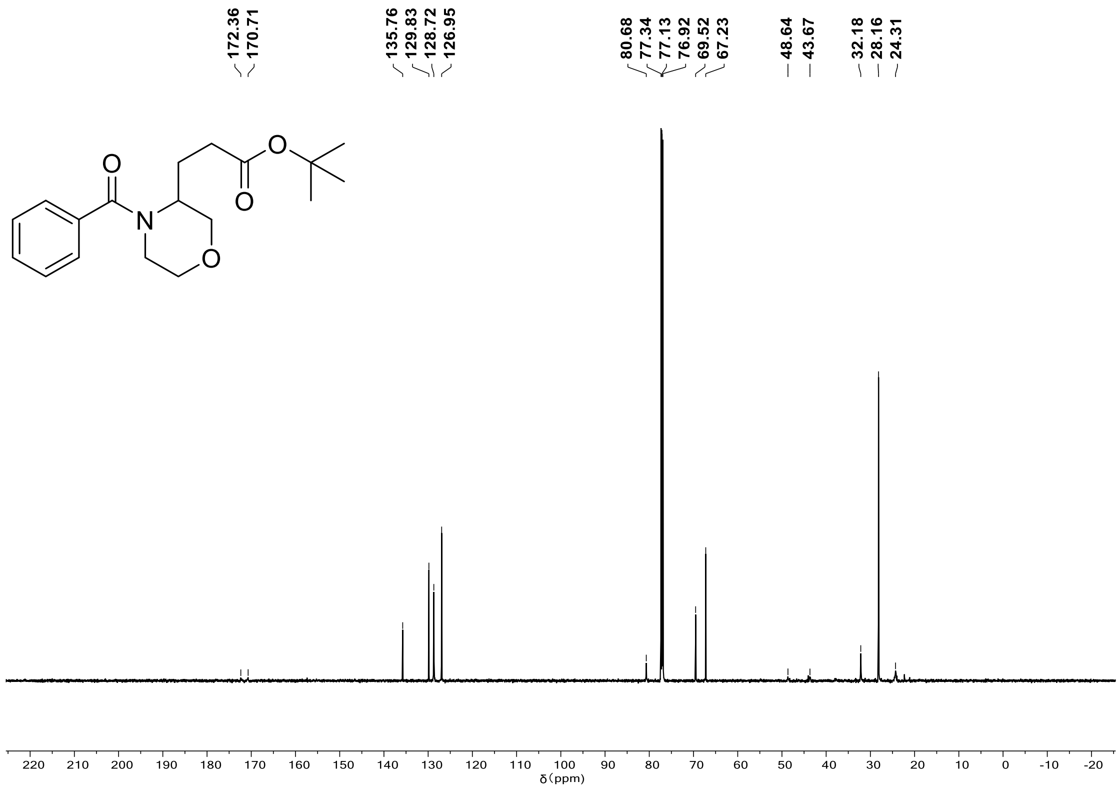

Figure S105. ^{13}C NMR spectrum of **3d** (151 MHz) in $CDCl_3$.

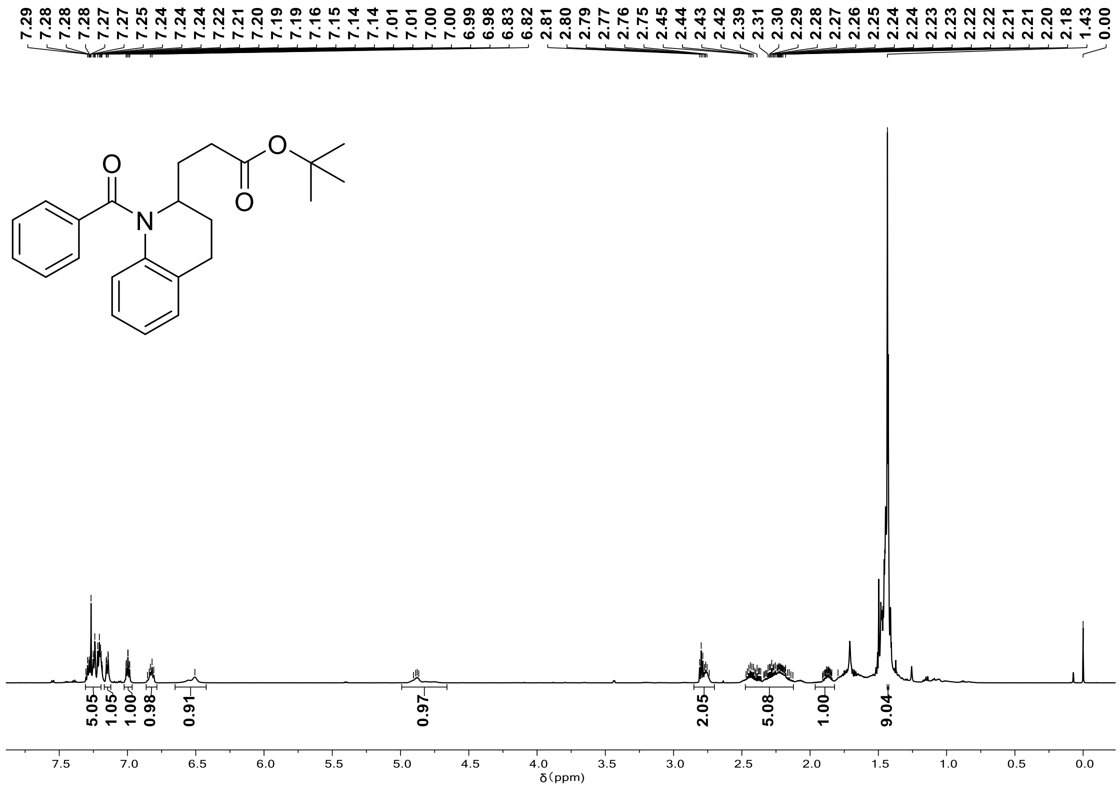

Figure S106. ^{1}H NMR spectrum of **3e** (600 MHz) in $CDCl_3$.

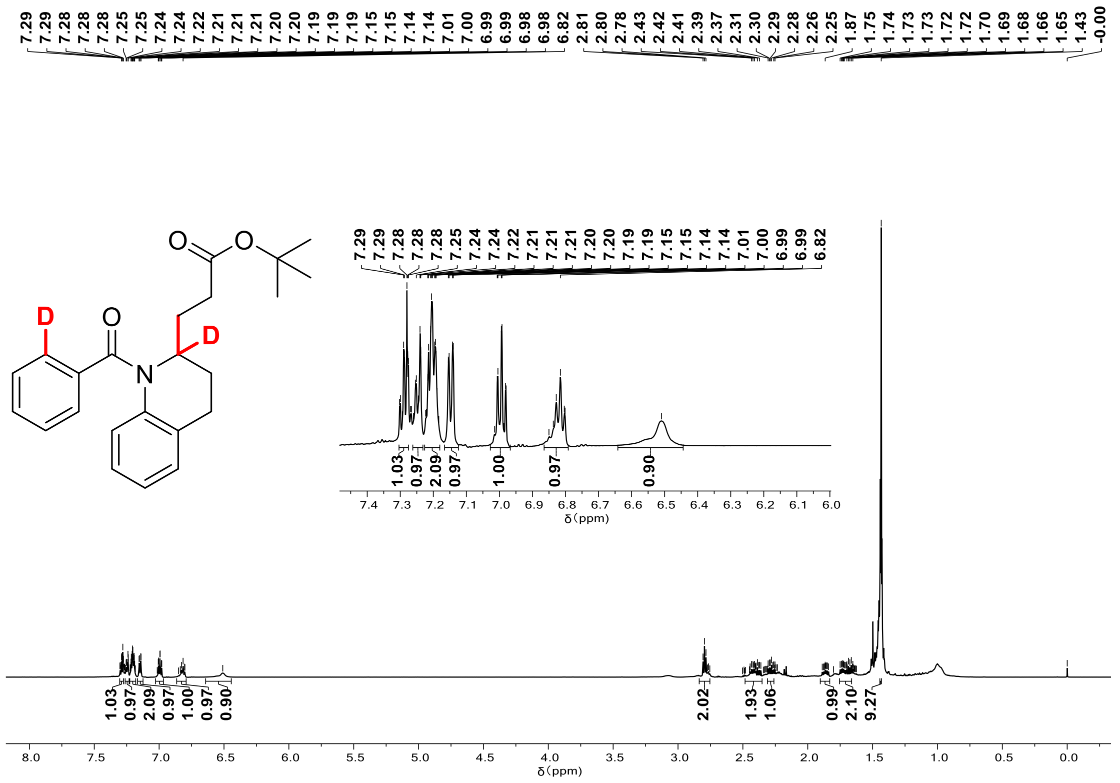

Figure S107. ^{1}H NMR spectrum of **3e-D** (600 MHz) in $CDCl_3$.

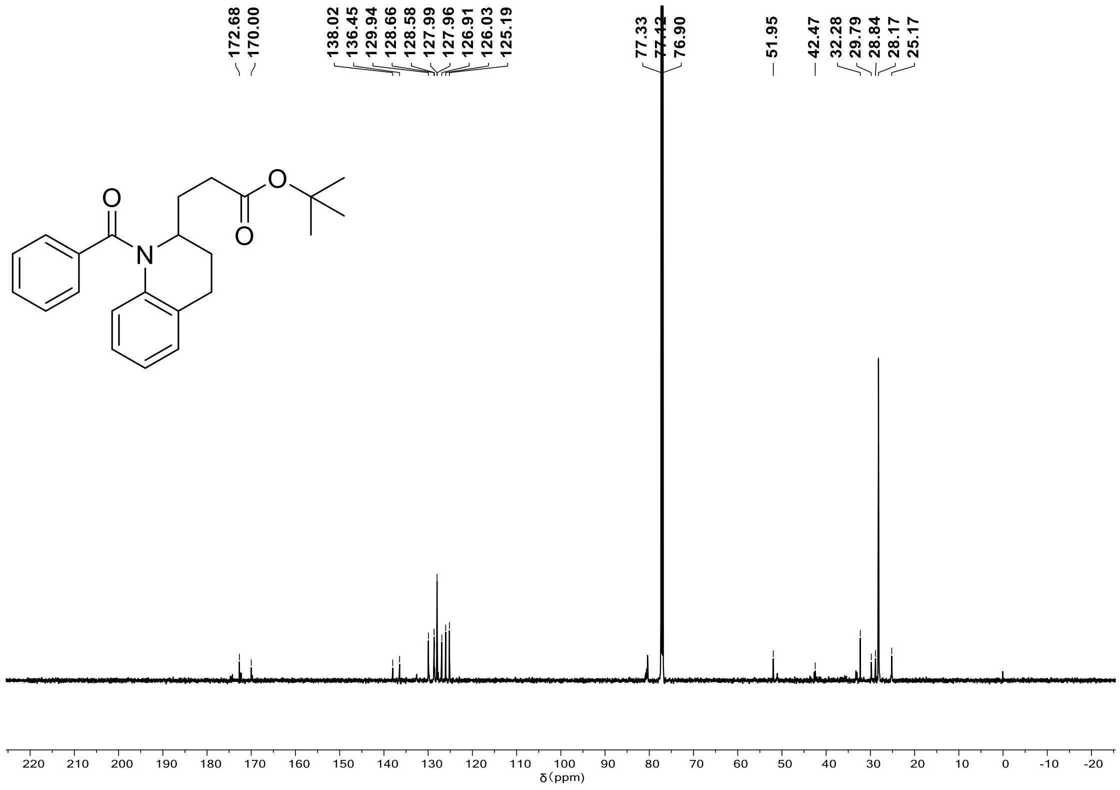

Figure S108. ^{13}C NMR spectrum of **3e** (151 MHz) in $CDCl_3$.

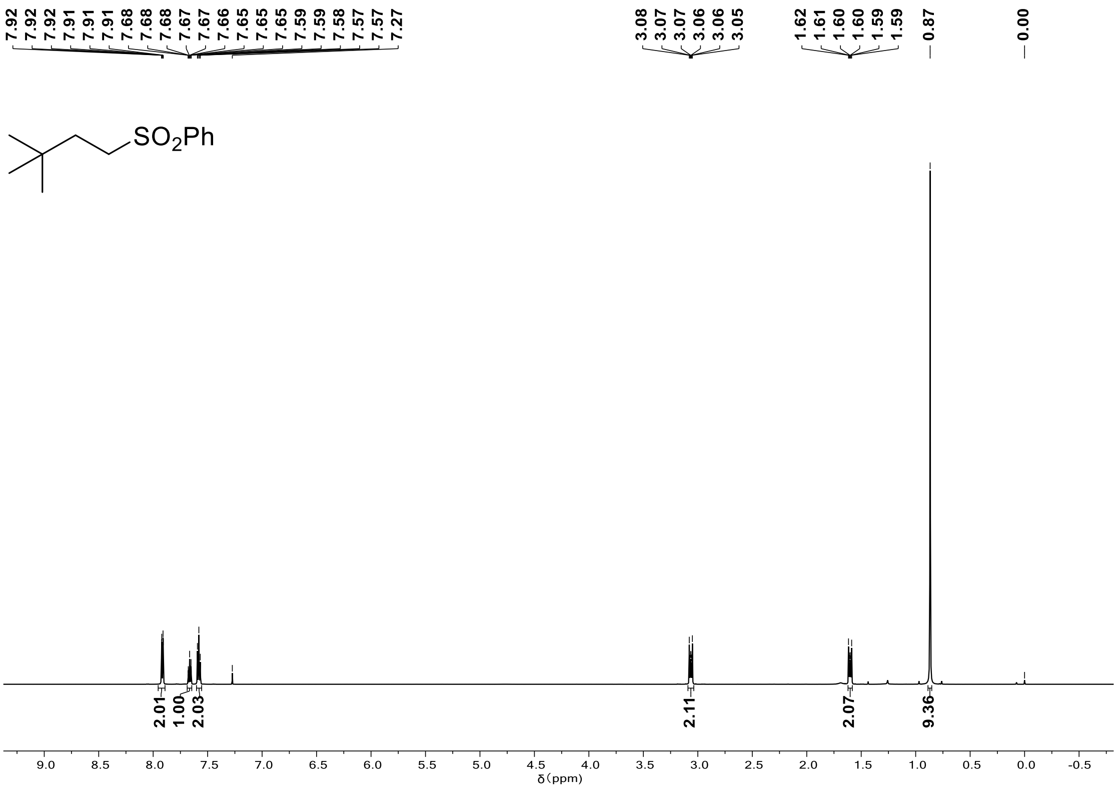

Figure S109. ^{1}H NMR spectrum of **4a** (600 MHz) in $CDCl_3$.

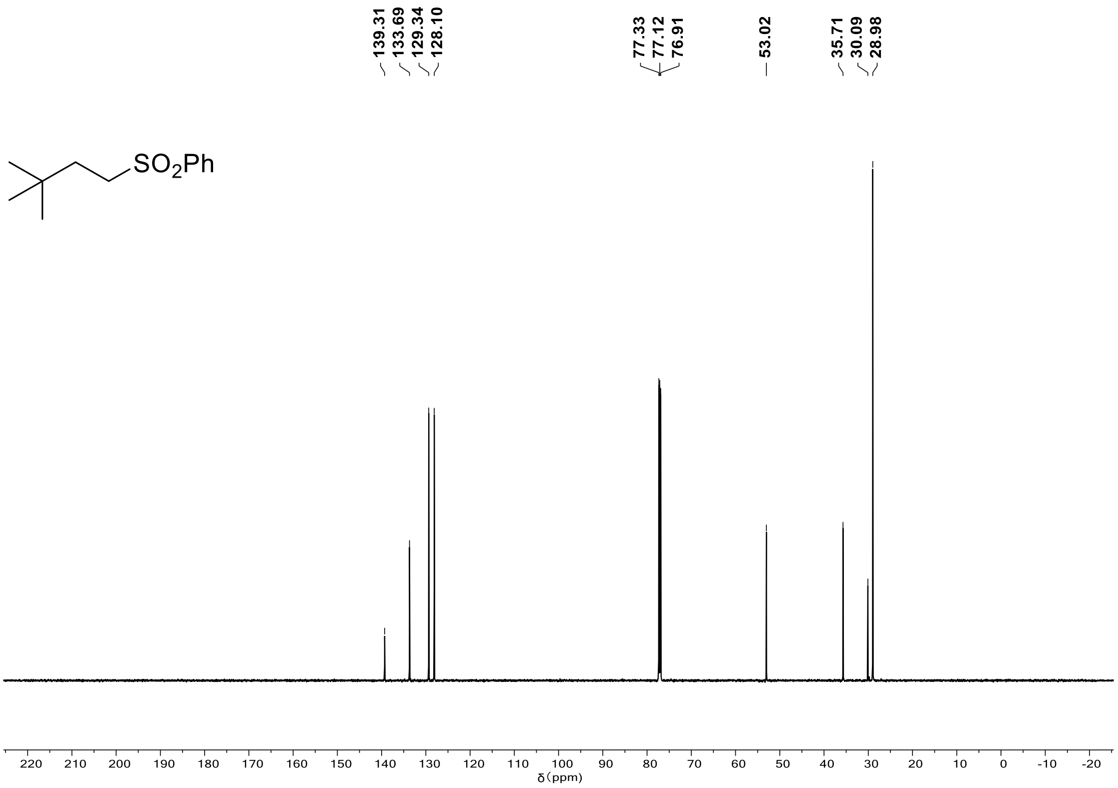

Figure S110. ^{13}C NMR spectrum of **4a** (151 MHz) in $CDCl_3$.

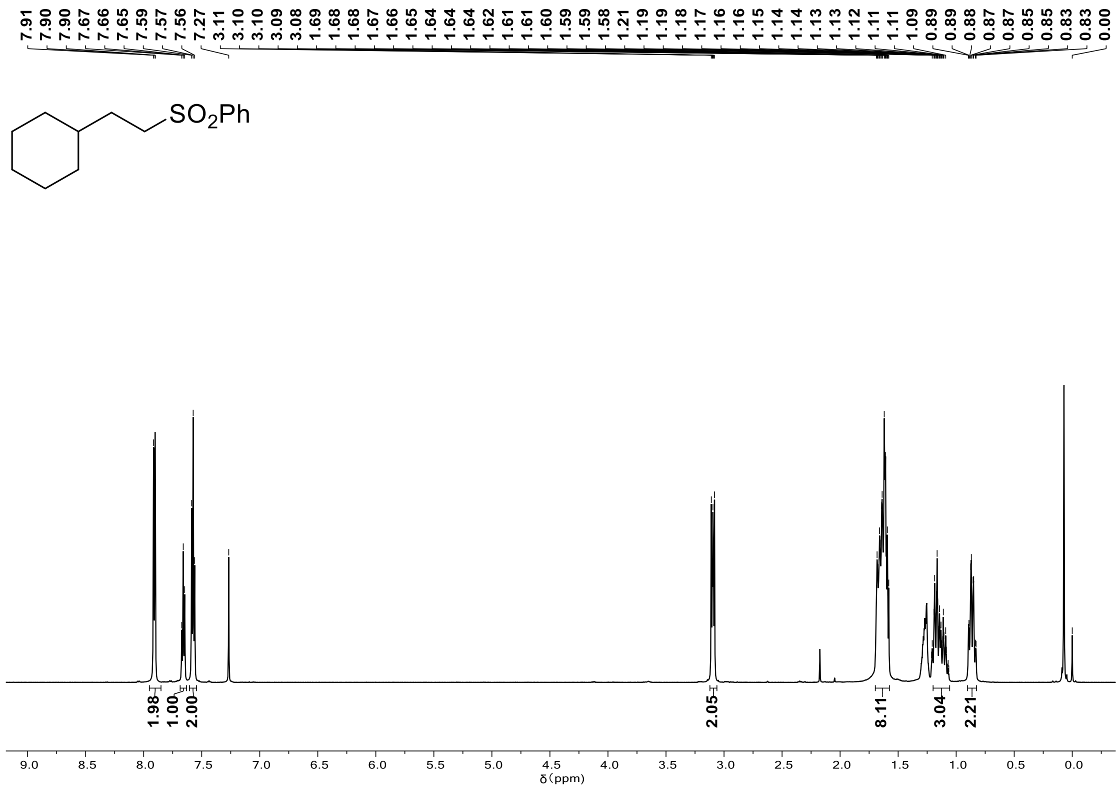

Figure S111. ^{1}H NMR spectrum of **4b** (600 MHz) in $CDCl_3$.

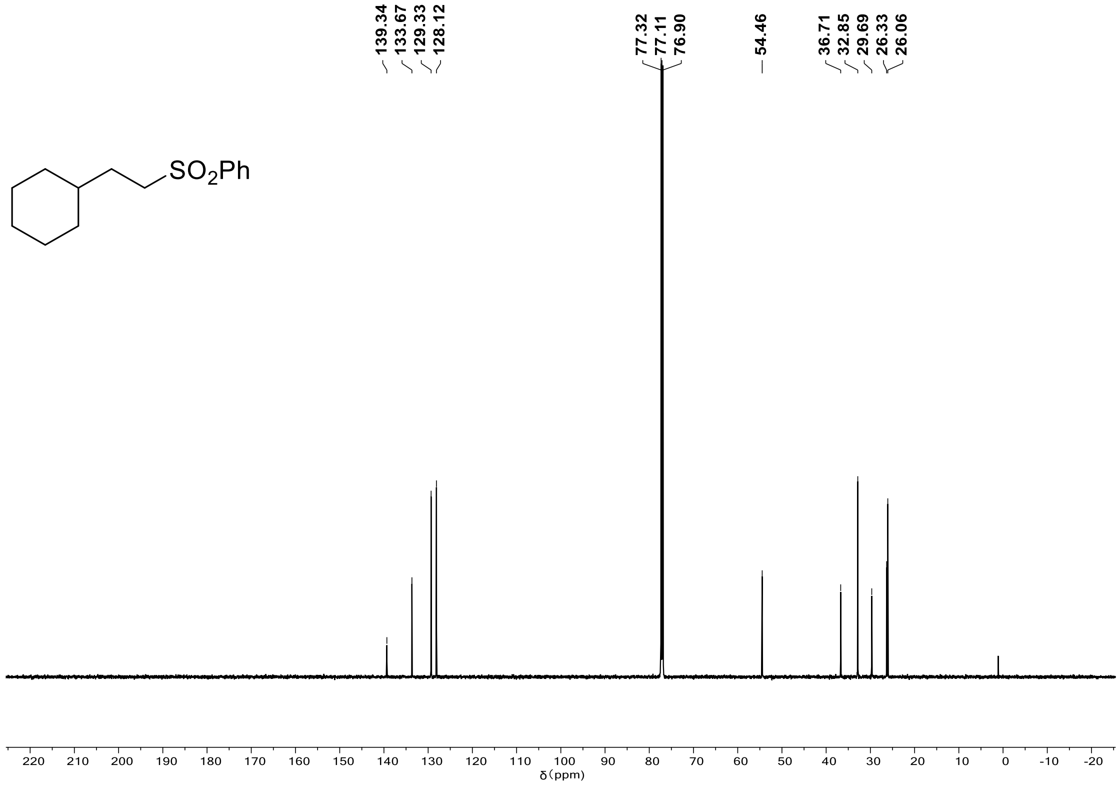

Figure S112. ^{13}C NMR spectrum of **4b** (151 MHz) in $CDCl_3$.

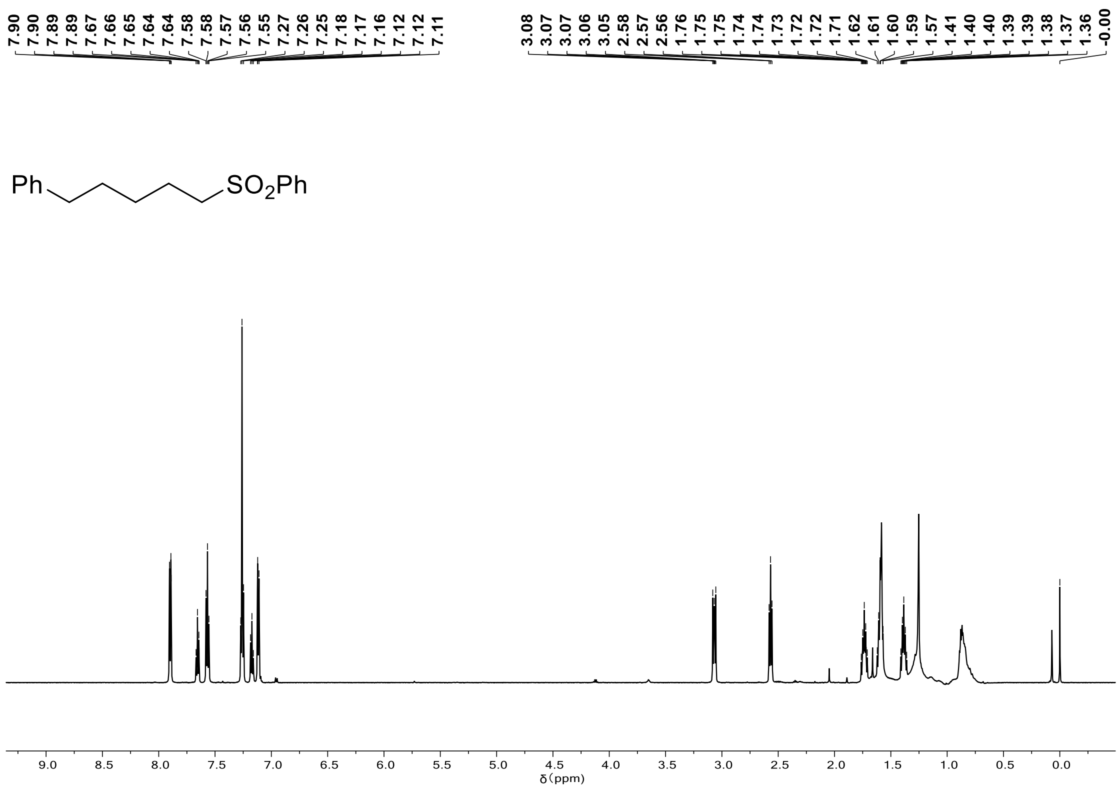

Figure S113. ^{1}H NMR spectrum of **4c** (600 MHz) in $CDCl_3$.

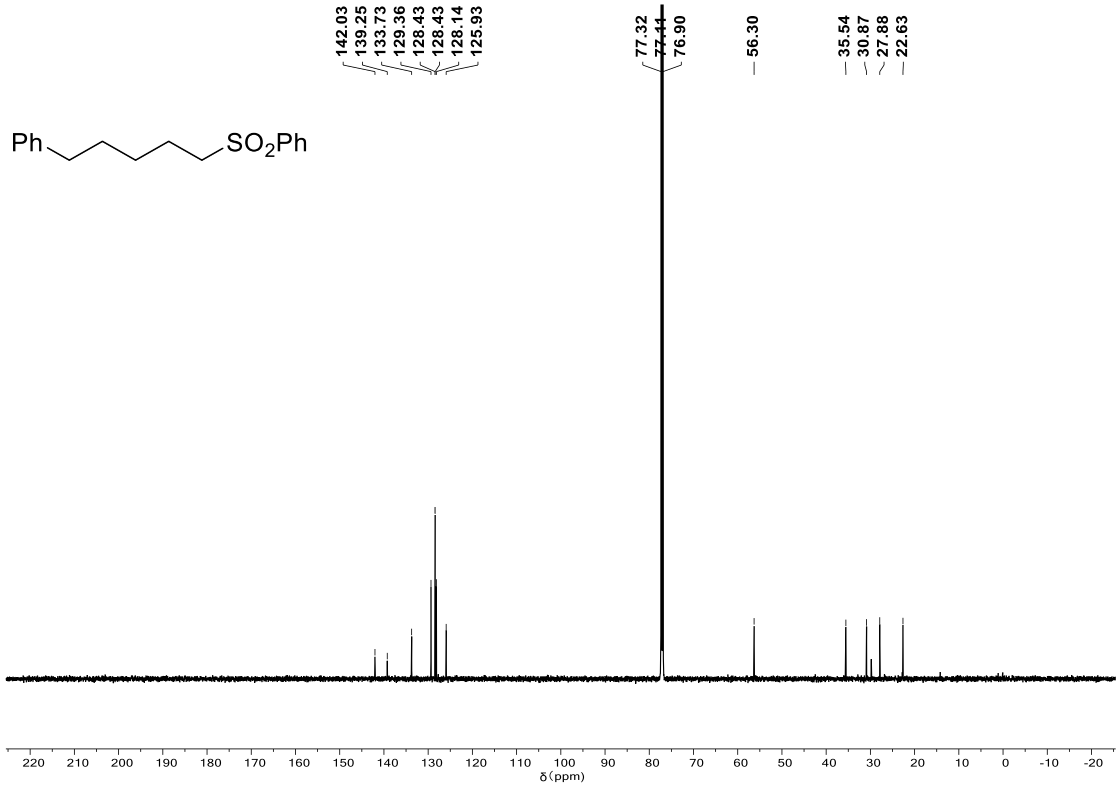

Figure S114. ^{13}C NMR spectrum of **4c** (151 MHz) in $CDCl_3$.

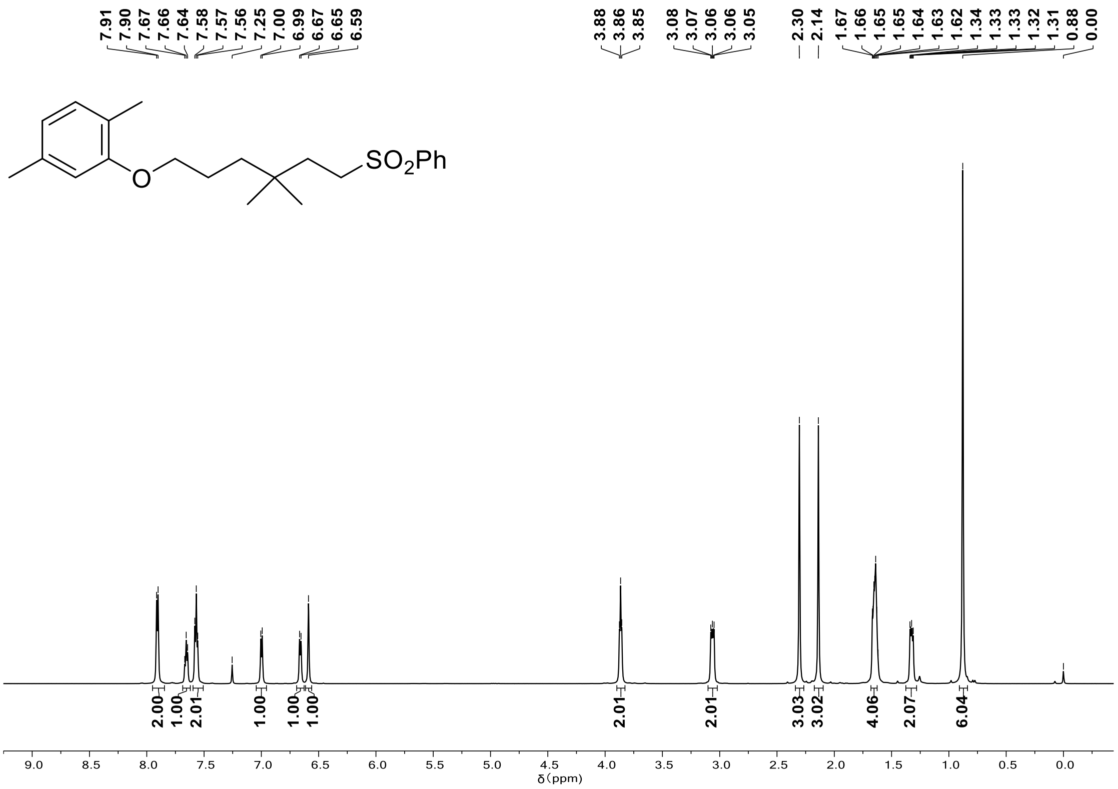

Figure S115. ^{1}H NMR spectrum of **4d** (600 MHz) in $CDCl_3$.

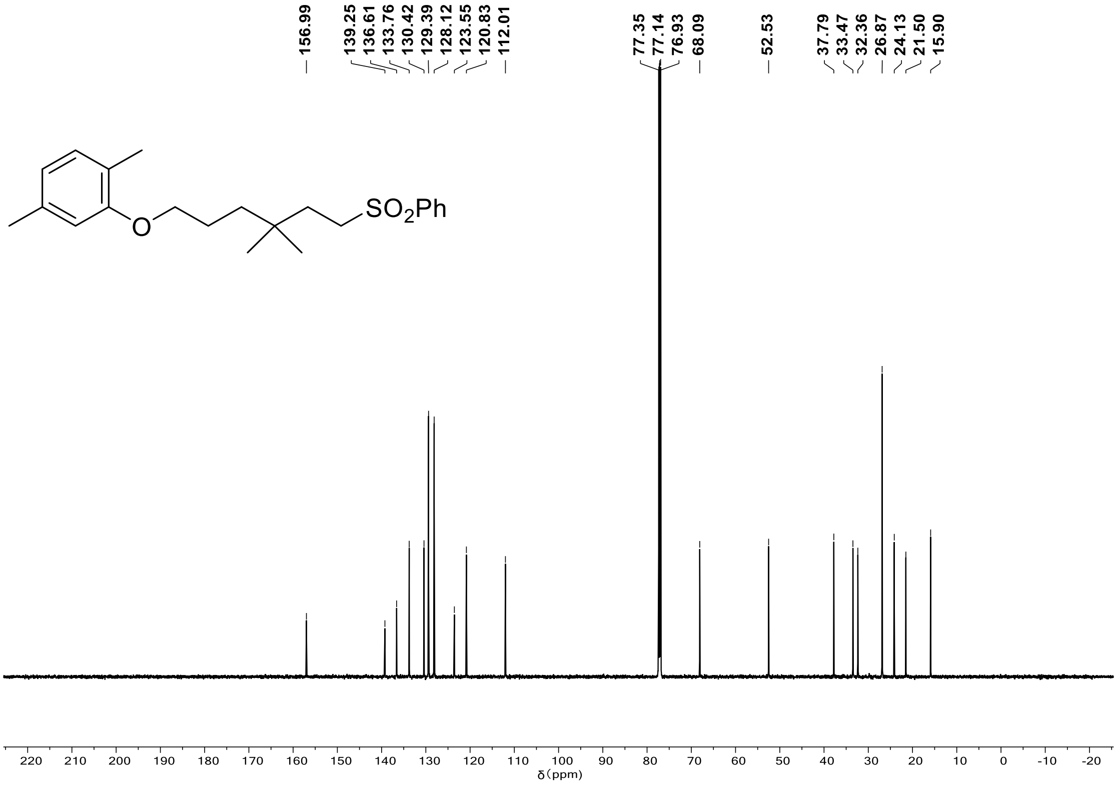

Figure S116. ^{13}C NMR spectrum of **4d** (151 MHz) in $CDCl_3$.

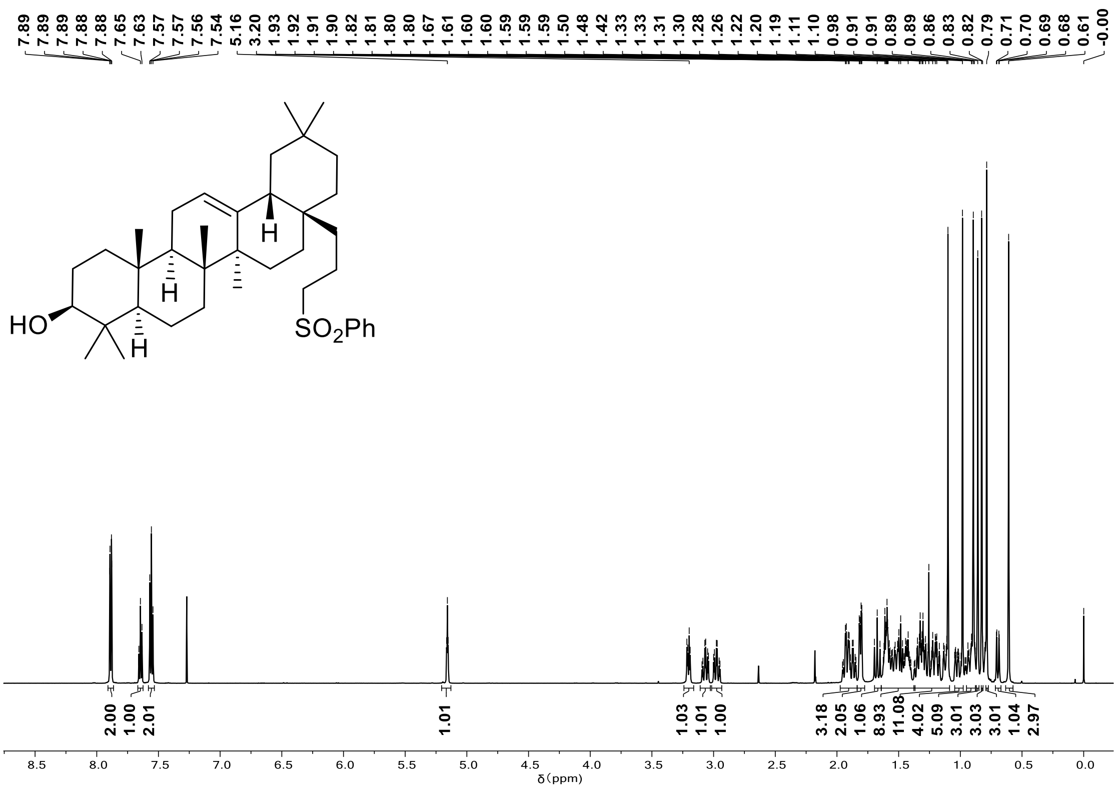

Figure S117. ^{1}H NMR spectrum of **4e** (600 MHz) in $CDCl_3$.

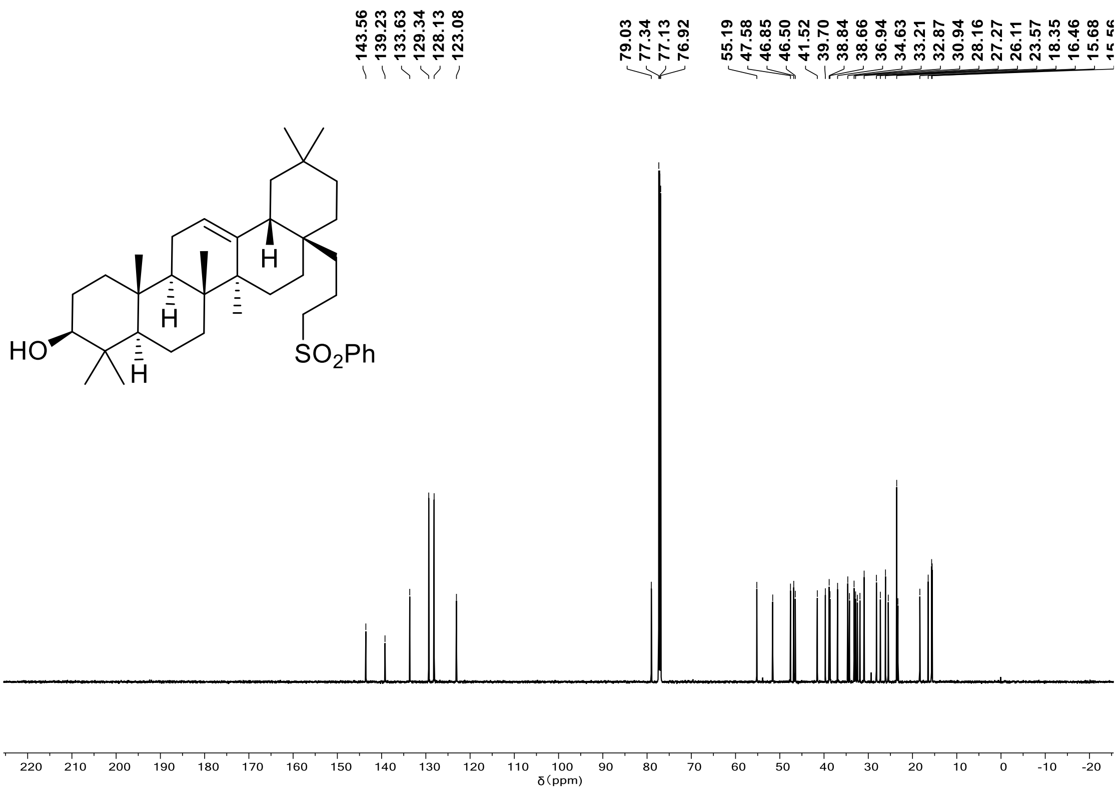

Figure S118. ^{13}C NMR spectrum of **4e** (151 MHz) in $CDCl_3$.

7. Reference

[1] T. Li, M. T. Kozlowski, E. A. Doud, M. N. Blakely, N. L. Rosi, *J. Am. Chem. Soc.* **2013**, *135*, 11688-11691.

[2] S. M. Cohen, Z. J. Zhang, J. A. Boissonnault, *Inorg. Chem.* **2016**, *55*, 7281-7290.

[3] Y. Zhao, D. G. Truhlar, *Theoretical Chemistry Accounts* **2008**, *120*, 215-241.

[4] F. Weigend, R. Ahlrichs, *Physical Chemistry Chemical Physics* **2005**, *7*, 3297-3305.

[5] J. J. P. Stewart, *Journal of Molecular Modeling* **2007**, *13*, 1173-1213.

[6] M. J. F. a. G. W. T. a. H. B. S. a. G. E. S. a. M. A. R. a. J. R. C. a. G., **2016**.

[7] R. D. a. T. A. K. a. J. M. Millam, **2019**.
